# Multiple valence states of Fe boosting SERS activity of Fe_3_O_4_ nanoparticles and enabling effective SERS-MRI bimodal cancer imaging

**DOI:** 10.1016/j.fmre.2022.04.018

**Published:** 2022-05-03

**Authors:** Jie Lin, Xuehua Ma, Anran Li, Ozioma Udochukwu Akakuru, Chunshu Pan, Meng He, Chenyang Yao, Wenzhi Ren, Yanying Li, Dinghu Zhang, Yi Cao, Tianxiang Chen, Aiguo Wu

**Affiliations:** a Cixi Institute of Biomedical Engineering, International Cooperation Base of Biomedical Materials Technology and Application, Chinese Academy of Science Key Laboratory of Magnetic Materials and Devices, Zhejiang Engineering Research Center for Biomedical Materials, Ningbo Institute of Materials Technology and Engineering, CAS, Ningbo 315201, China; b School of Engineering Medicine, Key Laboratory of Big Data-Based Precision Medicine (Beihang University), Ministry of Industry and Information Technology, Beihang University, Beijing 100191, China; c Advanced Energy Science and Technology Guangdong Laboratory, Huizhou 516000, China; d University of Chinese Academy of Sciences, Beijing 100049, China

**Keywords:** Fe_3_O_4_ NPs, SERS–MRI dual-modal nanoprobes, PICT, Multiple valence states, Cancer diagnosis

## Abstract

Developing novel nanoparticle-based bioprobes utilized in clinical settings with imaging resolutions ranging from cell to tissue levels is a major challenge for tumor diagnosis and treatment. Herein, an optimized strategy for designing a Fe_3_O_4_-based bioprobe for dual-modal cancer imaging based on surface-enhanced Raman scattering (SERS) and magnetic resonance imaging (MRI) is introduced. Excellent SERS activity of ultrasmall Fe_3_O_4_ nanoparticles (NPs) was discovered, and a 5 × 10^−9^ M limit of detection for crystal violet molecules was successfully obtained. The high-efficiency interfacial photon-induced charge transfer in Fe_3_O_4_ NPs was promoted by multiple electronic energy levels ascribed to the multiple valence states of Fe, which was observed using ultraviolet–visible diffuse reflectance spectroscopy. Density functional theory calculations were utilized to reveal that the narrow band gap and high electron density of states of ultrasmall Fe_3_O_4_ NPs significantly boosted the vibronic coupling resonances in the SERS system upon illumination. The subtypes of cancer cells were accurately recognized via high-resolution SERS imaging *in vitro* using the prepared Fe_3_O_4_-based bioprobe with high sensitivity and good specificity. Notably, Fe_3_O_4_-based bioprobes simultaneously exhibited *T_1_*-weighted MRI contrast enhancement with an active targeting capability for tumors *in vivo*. To the best of our knowledge, this is the first report on the use of pure semiconductor-based SERS-MRI dual-modal nanoprobes in tumor imaging *in vivo* and *in vitro*, which has been previously realized only using semiconductor–metal complex materials. The non-metallic materials with SERS–MRI dual-modal imaging established in this report are a promising cancer diagnostic platform, which not only showed excellent performance in early tumor diagnosis but also possesses great potential for image-guided tumor treatment.

## Graphical abstract

**Figure:**
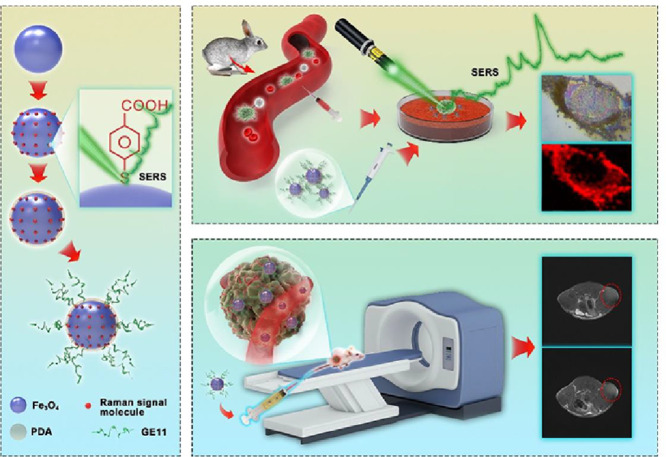

## 1 Introduction

Cancer is currently considered one of the most threatening diseases as nearly 10 million people die of cancer in the world every year [1]. Although the best strategy for cancer treatment is early diagnosis [2], cancer patients are often diagnosed at middle-advanced stages, so the rational treatment time is usually missed [3]. Magnetic resonance imaging (MRI) is a significant technology in disease diagnosis possessing the following advantages: no harmful radiation, detection of flow field *in situ*, and multi-parameter imaging, which are beneficial for tumor treatment and surgery *in vivo* [4], [5], [6]. However, its relatively low detection sensitivity, high technical requirements, and poor imaging resolution seriously impede its use in early clinical tumor diagnosis [7]. Recently, material-based MRI contrast agents have been reported to improve imaging resolution based on their unique physicochemical properties and tumor-targeting features [8], which partly expands the scope of MRI in tumor diagnosis and therapy. Although limited by spatiotemporal resolution in early cancer screening, MRI contrast agents play an irreplaceable role in enhancing signal contrast in tissues of interest [9]. Optical imaging modalities possess the features of micron-scale spatial resolution and non-harmful radiation, providing cellular level imaging resolution for tumors and significantly boosting the success rate of preoperative tumor diagnosis [10,11]. Fluorescence spectroscopy exhibits high sensitivity and good anti-interference ability as an oncological imaging modality. However, tissue autofluorescence and photobleaching hinder its practical application [12]. Recently, surface-enhanced Raman scattering (SERS) has emerged as a promising technique for cancer biosensing and clinical research [13], [14], [15], [16]. SERS imaging offers the advantages of nondestructive detection, ultrahigh sensitivity, selective enhancement, label-free analysis, nanoscale spatial resolution, and provides molecular fingerprint vibrational information [17], [18], [19], [20]. Hence, SERS imaging has attracted great interest for tumor component identification, circulating tumor cell detection, precise delineation of tumor margins, and drug delivery monitoring [11,[21], [22], [23], [24]].

SERS technology can provide detailed structural information through point-to-point spectra and mapping images based on nano–micro material detection schemes [25]. Noble metal materials with ultrahigh SERS enhancement factors (EFs) have been widely utilized in various detection and analysis fields owing to their large interfacial electromagnetic field produced by surface plasmon resonance under laser illumination [17,26]. Semiconductor-based SERS platforms based on the chemical enhancement mechanism are also widely applied in the fields of biological imaging and detection [27], [28], [29]. The semiconductor SERS platform is recognized as a promising analytical tool in cancer diagnosis and precision medicine because of its good biocompatibility, excellent spectral stability, selective SERS enhancement, and fingerprint vibration modes [30,31], which endow it with unique superiority in SERS imaging for tumor detection. Much less attention has been paid to semiconductor SERS substrates because of their relatively weak SERS EF and limitations of SERS optical modality for large-scale tumor tissue imaging. Fortunately, several semiconductor materials with metal-comparable SERS EF have been reported: they were prepared using controllable synthesis strategies, such as surface defect engineering [32,33], construction of an amorphous phase [34], *n-*/p-type element doping [35,36], crystal facet regulation [37], and designing two-dimensional nanomaterials [38]. These results indicate that the photon-induced charge transfer (PICT) efficiency in semiconductor SERS systems can be significantly improved by modifying the surface physicochemical electronic structure, which is the key point for boosting interfacial electron transfer between the SERS substrate and molecules [35]. Hence, exploring novel semiconductor nanomaterials with unique surface electronic structures is beneficial for establishing efficient interfacial electron transport channels, obtaining high-efficiency PICT, and magnifying the target molecular polarization tensor satisfying the highly sensitive SERS detection and imaging modes. SERS imaging of tumor tissues is limited to the nano/micro scale region because of point-by-point acquisition of laser spots, which greatly inhibits its further application in tumor imaging. The shortcomings of the SERS imaging method utilized in tumor tissues can be greatly augmented by the MRI mode because of the development of several semiconductor materials that can be used as MRI contrast agents [39,40], enabling SERS–MRI dual-modal imaging. The dual-modal imaging nanoprobes are therefore expected to exhibit prospective imaging capabilities for cancer diagnosis and treatment, ranging from the cell to tissue level.

Motivated by the above analysis and discussion, novel semiconductor-based SERS–MRI dual-modal imaging nanoprobes were successfully designed. Ultrasmall Fe_3_O_4_ nanoparticles (NPs) exhibit good SERS sensitivity with an EF of 9.06 × 10^3^, and 5 × 10^−9^ M limit of detection (LOD) for crystal violet (CV), possessing stronger SERS activity than Fe_2_O_3_ NPs. Multiple electronic energy levels derived from the multiple valence states of Fe play a vital role in improving the interfacial charge transfer in the Fe_3_O_4_ SERS system, as revealed by ultraviolet–visible (UV–vis) diffuse reflectance spectroscopy. Density functional theory (DFT) calculations indicated that Fe_3_O_4_ NPs possess a narrow band gap and high electron density of states (DOS), which are beneficial for the formation of stable ultrasmall Fe_3_O_4_-molecule SERS systems and generation of strong vibronic coupling resonance. These factors synergistically endow ultrasmall Fe_3_O_4_ NPs with an efficient PICT process, which magnifies the molecular polarization tensor and results in a strongly enhanced Raman signal. Circulating tumor cells were effectively detected using the Fe_3_O_4_-based SERS bioprobe, and subtypes of breast tumor cells could be readily distinguished via high-resolution SERS imaging. Notably, Fe_3_O_4_-based bioprobes can serve as ideal *T_1_*-weighted MRI contrast agents for tumor imaging in mice with active-targeting capability *in vivo*, thus achieving dual-modal SERS–MRI imaging ranging from the cell to tissue level. Fe_3_O_4_-based SERS–MRI bioprobes provide a new pathway for recognizing different subtypes of cancer cells and providing detailed oncological imaging information *in vivo* and *in vitro*, greatly improving the success rate of early cancer diagnosis. The reported MRI–SERS dual-modal bioprobes are prepared using Fe_3_O_4_–metal [41], [42] or Fe_3_O_4_–semiconductor complexes [43], in which Fe_3_O_4_ only offers MRI activity, and the SERS activity of Fe_3_O_4_ NPs is ignored. To the best of our knowledge, this is the first report on pure Fe_3_O_4_ material with SERS–MRI dual-modal activity for tumor imaging. Designing SERS–MRI dual-modal bioprobes can be regarded as an innovative and reliable strategy for highly sensitive and label-free cancer imaging. Moreover, dual-modal nanoprobes hold huge potential as a new tool for image-guided tumor treatment.

## 2 Materials and methods

### 2.1 Preparation of Fe_3_O_4_ nanoparticles

Ultrasmall Fe_3_O_4_ nanoparticles were synthesized via coprecipitation method. Briefly, 12 mmol of citric acid was dissolved in 80 mL deionized water, then the solution was heated up to 65 °C. Meanwhile, 80 mL of iron precursor solution (8 mmol of FeCl_3_ and 5 mmol of FeCl_2_) were quickly injected under magnetically stirred in a nitrogen atmosphere, followed by drip addition of 240 mL ammonia solution (5%). After 24 h, the ultrasmall Fe_3_O_4_ nanoparticles were synthesized. Furthermore, the products could be purified by dialyzing in deionized water for 6 times, by which the unreacted and residual ions were removed from the colloids.

### 2.2 Preparation of Fe_2_O_3_ nanoparticles

Ultrasmall Fe_2_O_3_ NPs were prepared by high temperature calcination of above Fe_3_O_4_ NPs at 650 °C in a tube furnace for 3 h. Furthermore, the product was rinsed with de-ionized water and ethanol several times, and dried in a vacuum oven at 80 °C for 10 h.

### 2.3 *Preparation of Fe_3_O_4_*–*AR*–*PDA*–*GE11 SERS bioprobe*

50 mL purified Fe_3_O_4_ NPs were mixed with 50 mL alizarin red (AR) solution (1 × 10^−4^ M) under mechanical stirring. After 16 h, Fe_3_O_4_–AR NPs was separated by centrifugation (10,000 rpm, 10 min), and washed twice to remove excess AR. Then, a simplified method was used to coat the surface of Fe_3_O_4_–AR NPs with polydopamine (PDA). In a facile procedure, 50 mL Fe_3_O_4_-AR (1 mg/mL in tris-HCl buffer solution, pH 8.5) was transferred into a 250 mL beaker, and 50 mL PDA (1 mg/mL in trimethylolamine-HCl buffer solution, pH 8.5) was added dropwise into it. The mixture was stirred for 4 h, separated by centrifugation and washed thrice with deionized water. The purified Fe_3_O_4_-AR-PDA bioprobes were re-immersed in PBS buffer solution for storage and future use. GE11 was conjugated to the surface of Fe_3_O_4_–AR–PDA through the reaction between GE11 (-COOH) and PDA (-NH_2_). Typically, GE11 (1.0 mg/mL in PBS, pH 7.4) and Fe_3_O_4_-AR-PDA (1.0 mg/mL in PBS, pH 7.4) solutions were mixed at equal volumes and stirred overnight at room temperature. After the reaction, the prepared Fe_3_O_4_–AR–PDA–GE11 dispersion was centrifuged and dispersed in ultrapure water.

### 2.4 Materials characterization

Transmission electron microscopy (TEM) and high-resolution TEM (HRTEM) images were obtained by Talos F200x. UV–vis diffuse reflectance/absorption spectra were collected on a UV-3600 UV–Vis-NIR spectrophotometer made by Shimadzu, Japan. X-ray photoelectron spectroscopy (XPS) was acquired by a Kratos Axis Ultra DLD instrument equipped with an Al anode (Al-Kα = 1,486.7 eV). Inductively coupled plasma optical emission spectrometry (ICP-OES) was measured an Optima 2100 instrument from Perkin Elmer. Photoluminescence spectra were collected by a He-Cd laser (325 nm) as excitation illumination. Infrared spectroscopy was acquired by intelligent Fourier infrared spectrometer (FTIR) (NICOLET 6700). Raman spectra and SERS images were obtained by Renishaw inVia Reflex instrument, England. X-ray diffraction (XRD) of the powder samples were characterized by the Rigaku Rotaflex Dmax2200 diffractometer (Japan) with Cu Kα radiation (λ = 1.54056 Å). MRI *in vivo* is obtained by 1.5 T human clinical scanner (Ingenia, Philips, Netherlands). MRI *in vitro* is acquired by MesoMR23 0.47 T scanner (Shanghai Niumag Corporation). Laser scanning confocal microscope (LSCM) images were obtained by Leica DMi8 (Germany).

### 2.5 SERS mapping

High-resolution SERS mapping imaging was acquired based on spot-to-spot Raman spectra collection on a 50 µm × 50 µm area. In this area, MCF-7 and MDA-MB-231 TNB tumor cells targeted by Fe_3_O_4_–AR–PDA–GE11 bioprobes were on Si platform. Plenty of SERS spectra was acquired by the acquisition platform with 1 µm scan step upon 532 nm laser illumination, and with acquisition time of 0.5 s. SERS mapping image was obtained by analyzing the Raman vibration peak (1,255 cm^−1^) of alizarin red (AR) molecule.

### 2.6 SERS spectrum

SERS experiment was conducted in water. Typically, Fe_3_O_4_ NPs water suspension was mixed with probe molecules to obtain a final solution concentration, and the Fe_3_O_4_ NPs-probe molecules were kept for 5 h. Then, highly diluted Fe_3_O_4_ NPs-probe molecule solutions with different concentrations were dropped onto a clean Si platform and thoroughly rinsed with water several times to remove the unabsorbed probe molecules. Fe_3_O_4_ NPs-probe molecules were then subjected to SERS analysis. SERS signal were collected 90 min later as the water was completely volatile.

### 2.7 Processing of rabbit blood sample

Animal experiments were conducted with the approved protocol of institutional animal care and use committee (IACUC). Rabbit blood samples were obtained from the heart of healthy rabbit. Cancer cells were added into rabbit blood samples to simulate CTCs environment. 1 mL PBS was added to 2 mL rabbit blood samples to dilute the blood sample, followed by the addition of 2 mL lymphocyte separation solution. The blood sample was centrifuged at 1,500 rpm for 20 min, and the obtained white layer was transferred to a centrifuge tube with 4 mL PBS (10 mmol). Then, the acquired sample was centrifuged at 1,000 rpm for 5 min, removing the supernatant using a pipette and leaving bottom cells (∼ 0.5 mL). 4 mL PBS solution (10 mmol) was added to the sample with 1,000 rpm centrifugation (5 min), followed by removing 3 mL supernatant. Then, the SERS bioprobe (200 µL) was dropped into the sediment solution, which was incubated at 37 °C for 30 min. Subsequently, precipitation was collected by centrifugation at 1,000 rpm for 5 min. Finally, the precipitate was evenly dispersed in the PBS solution (200 µl), and signal of SERS bioprobe targeted to cancer cell was acquired.

### 2.8 Cell culture

Human breast cancer cell lines MCF-7 and MDB-MA-231 were cultured in the DMEM medium supplemented with 10 wt% fetal bovine serum (FBS), 100 units mL^−1^ of penicillin, and 100 mg mL^−1^ of streptomycin. The cells were incubated at 37 °C in a humidified atmosphere containing 5% of CO_2_.

### 2.9 MRI *in vitro* and *in vivo*

MR imaging and relaxivity of the Fe_3_O_4_-based bioprobes were tested by MR analyzing system (MesoMR23–060H-I, Niumag, Shanghai) with the magnetic field of 0.47 T. Briefly, different concentrations (0.075, 0.15, 0.3, 0.6, 1.2 mM) of Fe_3_O_4_-based bioprobes in deionized water were used to measure their longitudinal relaxivity (r_1_) and transverse relaxivity (r_2_). *T_1_*-weighted MRI was performed with spin echo sequence (TR = 600 ms, TE = 18.2 ms). For MRI *in vivo*, MDB-MA-231 tumor bearing Balb/C nude mice (4–6 weeks) were purchased from Nanjing Cavins Biotechnology Co., Ltd (Nanjing, China). The mice were anesthetized by intraperitoneal injection of chloral hydrate solution (8 wt%), and then injected with 75 µL nanoprobe (1 mg/mL) through tail veins. The *T_1_*-weighted images were acquired using a 3.0 T human clinical scanner (Siemens, Germany) of HwaMei Hospital, University of Chinese Academy of Sciences, China.

### 2.10 Immunofluorescence staining experiment

For LSCM, 2.0 mL of MCF-7 or MDB-MA-231 cells in growth medium were seeded into each glass bottom dish with the size of ø15 at a density of 5 × 10^4^ cells/mL and allowed to adhere at 37 °C for 24 h. The growth medium was then replaced with a fresh one containing 0.15 mg/mL of Fe_3_O_4_-based bioprobes. After 4 h incubation, the cells were washed thrice with PBS. The cells were then fixed with 4% formaldehyde for 30 min, permeabilized with 0.1% triton for 5 min, blocked with 1.0% BSA for 30 min and treated with the mixture of Hoechst (5 µg/mL) and FITC phalloidine (5 U/mL) for 30 min at room temperature. The samples were simultaneously excited at 405, 488, and 543 nm and the fluorescent images at emission wavelengths of 420–480, 500–540, and 600–660 nm were observed by a LSCM (Leica, Germany), respectively.

### 2.11 ICP-OES experiment

2.0 mL of MCF-7 or MDB-MA-231 cells in growth medium were seeded into each well at a density of 400,000 cells/mL and allowed to adhere at 37 °C for 24 h. The growth medium was then replaced with a fresh one containing Fe_3_O_4_-based bioprobe (150 µg/mL). After further 3 h incubation, the cells were washed thrice with PBS to remove unabsorbed bioprobe. Afterward, the cells were detached with trypsin and collected into a tube. The collected cells were digested with aqua regia and the content of iron ion was determined by ICP-OES.

### 2.12 In vivo toxicity experiments

For *in vivo* toxicity, female Balb/C nude mice (4–6 weeks) purchased from Nanjing Cavins Biotechnology Co., Ltd (Nanjing, China) were divided into four groups (3 each group), and were intravenously injected with different concentration of bioprobes (1, 12.5, 25 mg/kg) and PBS as control. After 14 days, the mice were sacrificed and their major organs were subjected to the Haematoxylin and Eosin (H&E) staining and histopathological assessment.

### 2.13 Simulation methods

The spin-polarized density functional theory (DFT) computations were carried out using the Vienna ab initio simulation package (VASP v.5.4.1). During all calculations, the generalized gradient approximation (GGA) and the projector augments wave (PAW) pseudopotentials with the exchange and correlation in the Perdew-Burke-Ernzerhof (PBE) were employed to describe the ion-electro interaction. A kinetic-energy cut-off of 550 eV was used for the plane wave basis set. The convergence threshold was set as 10^−5^ eV in energy and 0.02 eV/Å in force, respectively. The DFT+*U* technique was applied to the Fe atoms to depict the strong on-site coulomb repulsion between the Fe d-shell electrons, where the *U-J* parameters was set to 4.3 eV. In this work, the bulk α-Fe_2_O_3_ crystallizes in the hexagonal structure with antiferromagnetic order and the bulk Fe_3_O_4_ crystallizes in the inverted cubic spinel structure with the magnetic moments of the Fe ions exist on tetrahedral sites antiparallel to the Fe ions exist on octahedral sites were constructed, respectively. For bulk α-Fe_2_O_3_, the Monkhorst-Pack Gamma-centered grids with a 5 × 5 × 2 mesh for relaxations and a 15 × 15 × 6 mesh for the calculation of DOS were used. For bulk Fe_3_O_4_, the Monkhorst-Pack Gamma-centered grids with a 3 × 3 × 3 mesh for relaxations and a 9 × 9 × 9 mesh for the calculation of DOS were used. To model the interaction between Fe_3_O_4_ (α-Fe_2_O_3_) and adsorbed molecule, the (001) surface of Fe_3_O_4_ (α-Fe_2_O_3_) was constructed using the 1 × 1 (2 × 2) slab models with 20 Å thick vacuum layer added along the z direction. For all slab models, the Monkhorst-Pack Gamma-centered grids with a 3 × 3 × 1 mesh for structure optimizations and a 6 × 6 × 1 mesh for the calculation of charge distributions were used. All structures were visualized using the program VESTA.

## 3 Results and discussion

### 3.1 Characterization of ultrasmall Fe_3_O_4_ NPs

**Fig. 1:**
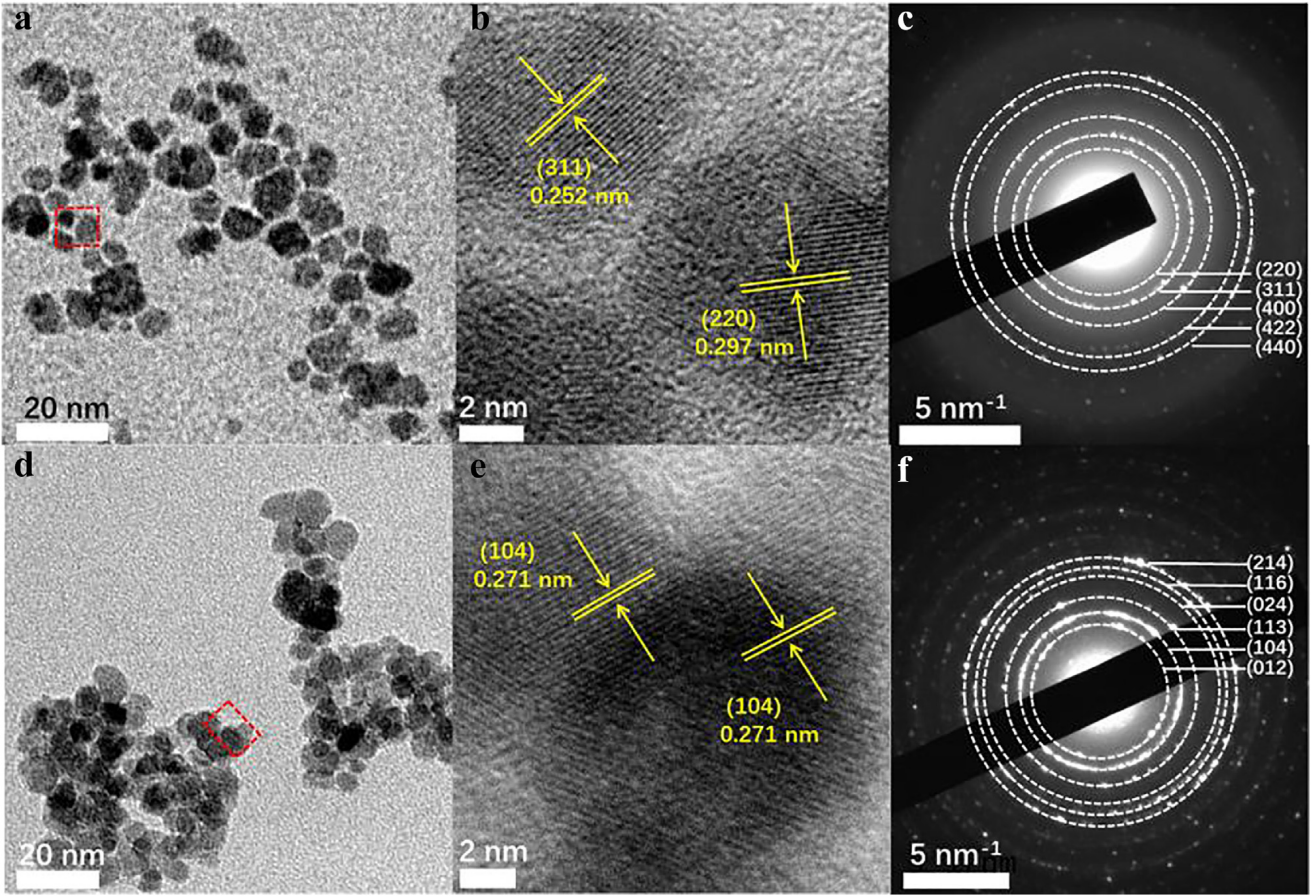
**TEM (a, d), HRTEM (b, e), and SEAD (c, f) images of ultrasmall Fe**_**3**_**O**_**4**_**and Fe**_**2**_**O**_**3**_**NPs, respectively.**

The efficiency of the PICT process in semiconductor SERS systems is strongly dependent on the electron transition paths from the substrate to the molecule [20,28]. Therefore, an ultrahigh SERS EF may be obtained by developing a novel semiconductor substrate with multiple electronic energy levels, which serve as extra springboards to assist PICT transitions. Hence, Fe_3_O_4_ nanomaterial was selected as a potential candidate with remarkable SERS activity owing to the multiple valence states of Fe. The synthesis protocol for ultrasmall Fe_3_O_4_ NPs was based on our previous report [44], with some modifications. Ultrasmall Fe_2_O_3_ NPs were successfully prepared via a high-temperature calcination synthetic route and served as a control SERS substrate. Images of Fe_3_O_4_ and Fe_2_O_3_ NPs obtained using transmission electron microscopy (TEM) are displayed in Fig. 1, showing that the sizes of the two acquired ultrasmall samples were approximately 5–8 nm. The broad-view TEM image (Fig. S1) demonstrates the relatively uniform size and homogeneous dispersity of the ultrasmall Fe_3_O_4_ NPs. High-resolution TEM (HRTEM) images (Fig. 1b, e) selected from the square region (Fig. 1a, d) illustrate that the observed lattice fringes (0.252, 0.297, and 0.271 nm) correspond to the (311) and (220) crystal planes of Fe_3_O_4_ and the (104) crystal plane of Fe_2_O_3_ NPs, respectively. The concentric rings in the selected-area electron diffraction patterns (Fig. 1c, f) were assigned to the (220), (311), (400), (422), and (440) lattice planes, and (214), (116), (024), (113), (104), and (012) lattice planes, which match well with the inverse spinel phase of Fe_3_O_4_ [45,46] and the hematite phase of Fe_2_O_3_ NPs [47,48], respectively. The crystal structures of ultrasmall Fe_3_O_4_ and Fe_2_O_3_ NPs were further verified using X-ray diffraction spectroscopy (Fig. S2) (JCPDS No. 82–1533; No. 33–0664).

### 3.2 SERS activity of ultrasmall Fe_3_O_4_ NPs

**Fig. 2:**
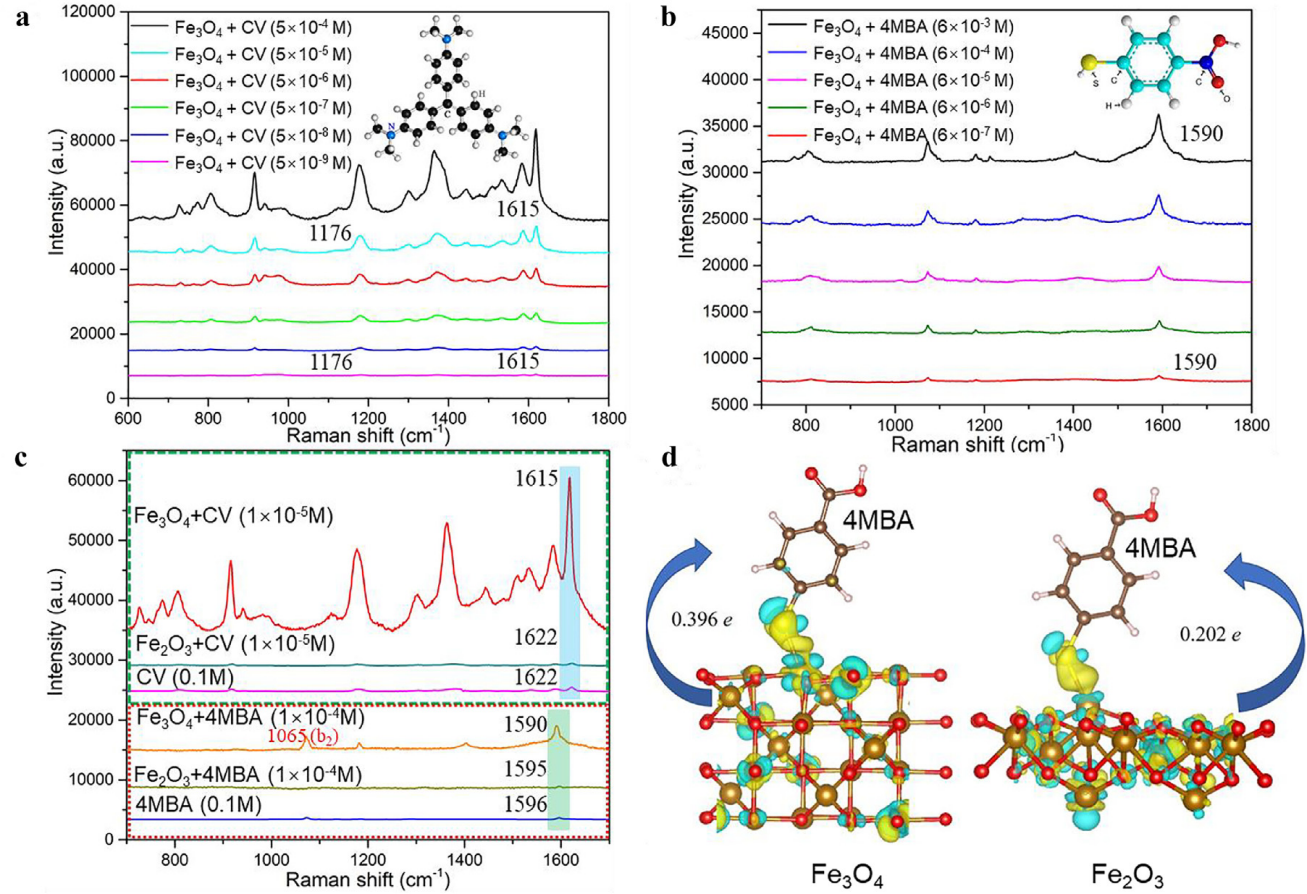
SERS spectra of (a) CV and (b) 4MBA molecules absorbed on Fe_3_O_4_ NPs at different concentrations, respectively. (c) Raman, SERS spectra comparison of CV and 4MBA molecules adsorbed on Fe_3_O_4_ and Fe_2_O_3_ NPs, respectively. Laser wavelength: 532 nm; laser power: 0.5 mW; lens: 50 × objective; and acquisition time: 1 s. (d) Charge difference redistributions of 4MBA absorbed on Fe_3_O_4_ and Fe_2_O_3_ NPs. The yellow and blue colors stand for the electron accumulation and depletion regions, respectively, and the charge transfer direction and values are also indicated. (For interpretation of the references to colour in this figure legend, the reader is referred to the web version of this article.)

The prepared ultrasmall Fe_3_O_4_ NPs with good dispersity are believed to be a potential substrate for studying the SERS activity of metal oxides, leveraging the multiple valence states of Fe. SERS measurements of Fe_3_O_4_ NPs were performed with CV, 4-mercaptobenzoic acid (4MBA), rhodamine 6 G (R6G), and 4-aminothiophenol (4ATP) probe molecules (Figs. 2a–b, S3). Based on the above SERS spectra, Fe_3_O_4_ substrate exhibited remarkable SERS activity: the molecular LOD of Fe_3_O_4_ NPs SERS substrate reached 5 × 10^−9^ M. Even as the CV concentration was decreased to 10^−9^ M, the Raman vibration peaks (1176 cm^−1^ vibration mode: C–C stretching, 1,615 cm^−1^ vibration mode: ring stretching) [49] of CV were still observable. As the concentrations of 4MBA and 4ATP probe (non-resonance) molecules were reduced to 6 × 10^−7^ M, the ring stretching vibration modes (4MBA: 1,590 cm^−1^, 4ATP: 1,580 cm^−1^) [34] were still intense. The EF of the CV molecule on Fe_3_O_4_ NPs was calculated to be approximately 9.06 × 10^3^, and the selected CV molecular concentration for EF calculation was 5 × 10^−5^ M, avoiding false EF values caused by the supersaturation adsorption effect (Fig. S4). The EF for CV molecules adsorbed on Fe_3_O_4_ NPs was calculated according to the following equation [38,50], and the non-SERS signal intensity was directly acquired in solution.

$$EF=\left(I_{SERS}/N_{ads}\right)/\left(I_{Raman}/N_{Raman}\right)$$

where *N_ads_* and *N_Raman_* are the numbers of CV molecules adsorbed on the Fe_3_O_4_ NPs and non-SERS solution samples under the same laser illumination, respectively; *I_SERS_* and *I_Raman_* are the SERS peak (1615 cm^−1^) intensity of CV molecules on Fe_3_O_4_ NPs and the non-SERS solution sample signal of CV molecules, respectively. The laser spot size (532 nm) was approximately 1.3 µm, The laser spot size is determined by the illumination laser wavelength (λ) and numerical aperture of the objective (50 ×; *N. A*: 0.5), *Spot size =* 1.22 *λ / (N. A)*. For non-SERS solution spectra measurement, the 0.02 M CV in ethanol was used, the focal plane of the 532 nm laser was 1.32 µm^2^, the depth of field penetration was *(h) = n λ / (N. A)^2^*, where *n* is the refractive index of the surrounding media, *h* = 2.89 µm. *N_Raman_* = 0.02 mol/*L* × 1.32 µm^2^ × 2.89 µm × 6.02 × 10^23^ mol^−1^. *N_Raman_* was estimated to be approximately 4.60 × 10^7^. The number of molecules adsorbed on Fe_3_O_4_ NPs is co-determined by laser spot size (532 nm: ∼1.3 µm), and the density of CV molecules adsorbed on the Fe_3_O_4_ NPs (∼0.5 nM cm^−2^)[38, 50]. To avoid supersaturated adsorption, the concentration of 5 × 10^−5^ M was selected (Fig. S4a). The effective adsorption coverage under laser illumination was evaluated based on the TEM images of the Fe_3_O_4_ NPs (Fig. S1), the SERS NPs in the total laser exposure area were estimated to be approximately 1/7, and the remaining 6/7 was the blank region. Therefore, *N_ads_* = 0.5 nM cm^−2^ × 6.02 × 10^23^ mol/*L* × π × (0.65 µm)^2^ × 1/7. The number of molecules adsorbed on the Fe_3_O_4_ NPs was determined to be approximately 5.71 × 10^5^. *I_SERS_* and *I_Raman_* are the SERS peaks, and normal Raman peak intensities at 1,615 cm^−1^ of CV molecules, which are based on 10 different laser spot acquisitions, *I_SERS_* = ∼22,500 and *I_Raman_* = ∼400 (Fig. S4b). The integration times for the SERS and Raman measurements were 1 and 2 s, respectively. Taking the above values into equation (1), EF was estimated to be approximately 9.06 × 10^3^.

The SERS activities of the Fe_3_O_4_ and Fe_2_O_3_ NPs were compared (Fig. 2c). The Fe_3_O_4_ NPs exhibited a much higher SERS enhancement than Fe_2_O_3_ NPs. The ring stretching vibration modes of CV (1,615 cm^−1^) and 4MBA (1590 cm^−1^) absorbed on the Fe_3_O_4_ NPs were significantly enhanced. The surface potentials of Fe_3_O_4_ and Fe_2_O_3_ NPs were measured as zeta-potentials, and the results were approximately 15.950 ± 3.61 and –0.175 ± 3.59 mV, respectively, which demonstrates that Fe_3_O_4_ NPs had positively charged surface, and Fe_2_O_3_ NPs were almost electroneutral. CV and R6G are both positively charged [33], and the electrostatic adsorption effect between the SERS substrate and molecule induces the adsorption of more positively charged molecules (CV, R6G) on Fe_2_O_3_ NPs. However, the observed SERS signal of the two positively charged molecules (CV and R6G) was enhanced on the Fe_3_O_4_ NPs (Fig. 2c, S5a), which illustrates that surface potential differences are not the primary factor for SERS EF in Fe_3_O_4_–molecule, and Fe_2_O_3_ NP–molecule surface complexes. For the Fe_3_O_4_@CV SERS system, a significant Raman intrinsic peak shift of the CV molecule was observed from 1,622 to 1,615 cm^−1^, whereas this Raman peak shift in the Fe_2_O_3_@CV SERS system was indistinguishable. Similarly, a noticeable 4MBA Raman peak shift was observed in the Fe_3_O_4_@4MBA SERS system. These results indicate that stronger interactions occur in the Fe_3_O_4_ substrate-molecule SERS system than in Fe_2_O_3_ NPs.

### 3.3 PICT promoted by multiple electronic energy levels

The mentioned Raman peak shift is an indicator of PICT of chemical enhancement [37]. The selective enhancement of non-totally symmetric vibration modes (b_2_ modes) was also observed in the control SERS measurements of Fe_3_O_4_ and Fe_2_O_3_ NPs (Fig. 2c). The C–H vibration peak (1065 cm^−1^) was assigned to the b_2_ mode of the 4MBA molecule [38], which was more enhanced than the other Raman peaks in the Fe_3_O_4_ SERS system. In addition, similar enhancements of the b_2_ modes at 1140, 1,385, and 1,445 cm^−1^ [49] were observed in the Fe_3_O_4_@4ATP SERS system (Fig. S5b), exhibiting noticeable and selective SERS enhancement compared to other Raman vibration modes. The intense SERS enhancement in Fe_3_O_4_ NPs ascribed to the PICT mechanism was verified via the selectively enhanced b_2_ modes, which matched well with the Herzberg–Teller selection rule [51]. To further investigate the interfacial charge transfer between the NPs and probe molecules, the charge density redistributions of the Fe_3_O_4_@4MBA and Fe_2_O_3_@4MBA SERS systems were quantitatively calculated using DFT. As shown in Fig. 2d, the 4MBA molecules were bonded to the surfaces of Fe_3_O_4_ and Fe_2_O_3_ via S–Fe bonds [20,37], which served as the interfacial charge transfer channel and facilitated the redistribution of the electron cloud around the 4MBA molecules and SERS substrate. The results show that charge density deformation mainly occurred around the S atom of 4MBA, where the charge accumulation region was concentrated on S atoms (yellow region), and the charge depletion region was mainly around Fe atoms (blue region) in the SERS platforms. Bader charge analysis indicated that the electron transfer direction was from the SERS substrate to the probe molecule, and the charge transferred from Fe_3_O_4_ to the 4MBA molecule was 0.396 *e*, which was greater than that for Fe_2_O_3_ (0.202 *e*). The results directly confirmed the interfacial PICT enhancement mechanism and demonstrated that the Fe_3_O_4_ substrate enabled more electrons to be transferred to the probe molecule, suggesting a higher-efficiency PICT and stronger SERS activity.

**Fig. 3:**
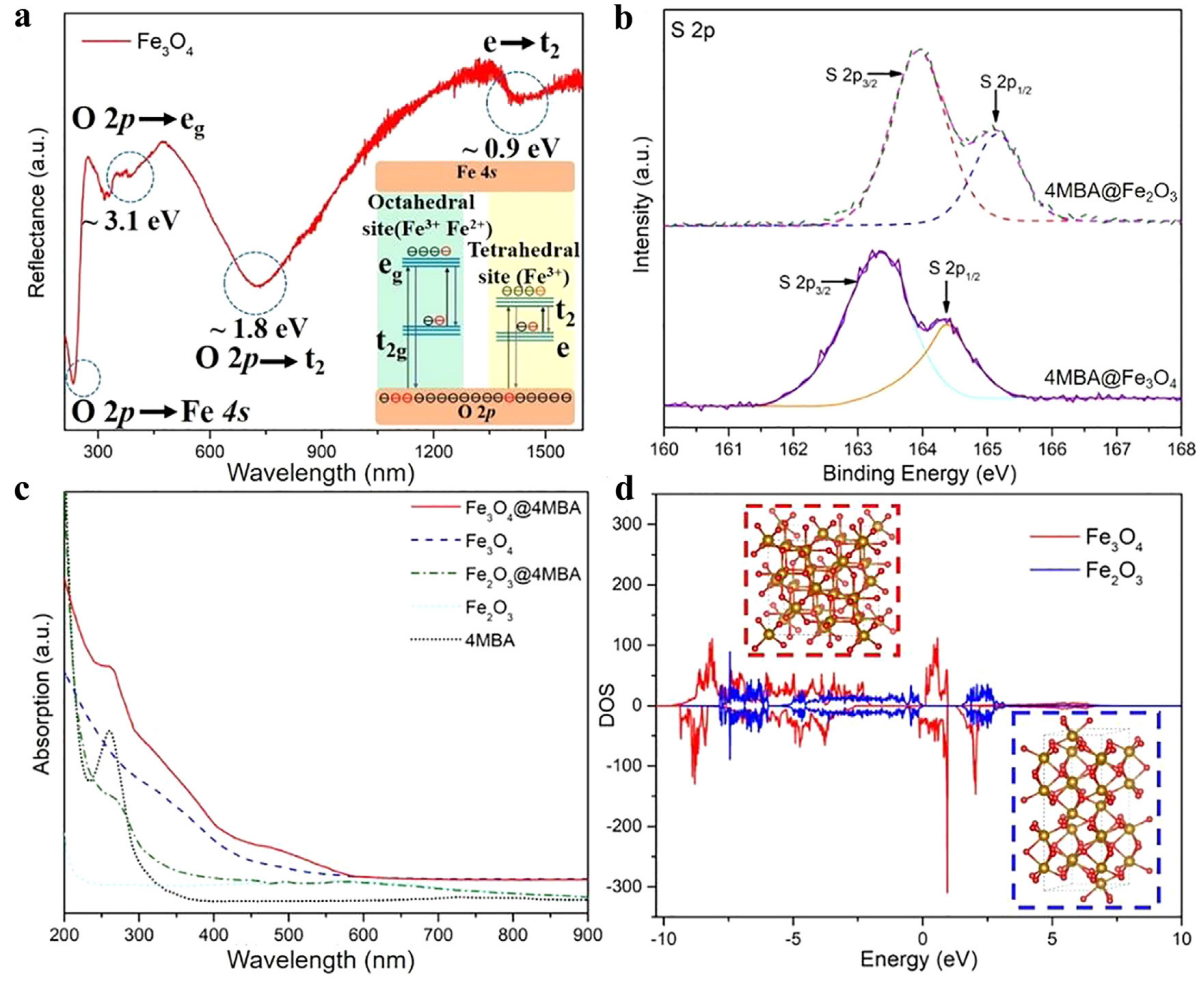
(a) UV–vis diffuse reflectance spectrum of Fe_3_O_4_ NPs, and the energy level structure in forbidden band of Fe_3_O_4_ NPs. (b) XPS measurements of 4MBA@Fe_2_O_3_ and 4MBA@Fe_3_O_4_ SERS system. (c) UV–vis absorption spectra of Fe_3_O_4_@4MBA, Fe_2_O_3_@4MBA, Fe_3_O_4_, Fe_2_O_3_, and 4MBA; Fe_3_O_4_, Fe_2_O_3_: 100 µg/mL, 4MBA: 1 × 10^−4^ M. (d) Electronic DOS of Fe_3_O_4_ and Fe_2_O_3_ NPs on the basis of DFT calculation, and the corresponding slab model of Fe_3_O_4_ and Fe_2_O_3_ NPs.

To perform an in-depth investigation of the SERS effect of Fe_3_O_4_ NPs originating from the specific surface physicochemical electronic structure, a high-efficiency PICT for ultrasmall Fe_3_O_4_ NPs was systematically studied via UV–vis diffuse reflectance/absorption spectroscopy, X-ray photoelectron spectroscopy (XPS), and first-principles DFT simulations (Fig. 3). Ferric and divalent Fe-induced multiple electronic energy levels exist between the O *2p* valence band (VB) and the empty Fe *4s* conduction band [52], [53], [54]. The crystal field bands of Fe_3_O_4_ NPs are mainly composed of multiple electronic energy levels derived from the multiple valence states of Fe occupying octahedral (Fe^3+^, Fe^2+^) and tetrahedral sites (Fe^3+^), which are composed of 3d metal atomic orbitals in Fe_3_O_4_ NPs [52]. The energy level structure of Fe_3_O_4_ NPs was explored using UV–vis diffuse reflectance and photoluminescence (PL) measurements. The electronic transitions from VB (O *2p*) to energy level e_g_ (∼3.1 eV), VB (O *2p*) to energy level t_2_ (∼1.8 eV), and energy level e to t_2_ (∼0.9 eV) were clearly observed in the UV–vis diffuse reflectance spectra shown in Fig. 3a. A clear PL peak (∼590 nm, ∼2.1 eV) of the Fe_3_O_4_ NPs was attributed to the radiative recombination of excitons transferred from energy levels e_g_ to t_2g_ (Fig. S6). These results confirm the existence of e_g_, t_2g_, e, and t_2_ crystal field bands in the forbidden energy gap of the Fe_3_O_4_ NPs, which provide more electron transfer routes and enable high-efficiency interfacial PICT from the SERS substrate to the probe molecule owing to multiple electronic energy levels.

Efficient PICT promoted by multiple electronic energy levels was further verified using XPS and UV–vis absorption spectroscopy. The binding energy of S *2p* electrons in the 4MBA@Fe_3_O_4_ surface complex was more negatively shifted than that of 4MBA@Fe_2_O_3_, demonstrating that a larger amount of electron transfer-induced nuclear potential offset occurred in the 4MBA@Fe_3_O_4_ SERS system (Fig. 3b) [30,55]. In addition, the XPS results suggested the occurrence of an efficient charge transfer between Fe_3_O_4_ NPs and 4MBA, which greatly increased the molecular polarizability and molecular Raman scattering cross-section. Moreover, 4MBA adsorbed on Fe_3_O_4_ NPs exhibited an intense absorption peak than on Fe_2_O_3_ in the visible region (∼530 nm), as shown in Fig. 3c, which was ascribed to the high-efficiency PICT resonance with light illumination. Wavelength-dependent SERS measurements of 4MBA on Fe_3_O_4_ NPs were also performed (Fig. S7). An extremely high SERS spectral signal was acquired under 532 nm laser illumination, which is consistent with the UV–vis absorption spectroscopy results. In brief, multiple valence states of Fe-induced electronic energy levels in Fe_3_O_4_ materials, such as crystal field bands of octahedral (Fe^3+^, Fe^2+^) and tetrahedral sites (Fe^3+^), could significantly promote interfacial electron escape from the Fe_3_O_4_ substrate and its subsequent electron transfer to the probe molecule, which enabled a more facile and efficient PICT. Notably, there was almost no surface plasmon resonance absorption peak around 532 nm (Fig. 3c); hence, the electromagnetic enhancement mechanism was ruled out. To further explore the remarkable SERS activity of Fe_3_O_4_, the electronic DOS of ultrasmall Fe_3_O_4_ and Fe_2_O_3_ were calculated using DFT simulations (Fig. 3d). The results demonstrate that Fe_3_O_4_ NPs exhibit a significantly larger electronic DOS compared to the Fe_2_O_3_ NPs, especially for the DOS near the Fermi energy level. The projected DOS indicates that the total DOS near the Fermi energy level for Fe_3_O_4_ NPs was contributed by the Fe ions existing in the octahedral and tetrahedral sites (Fig. S8), which is consistent with the UV–vis diffuse reflectance spectroscopy results. The abundant electronic DOS in the vicinity of the Fermi energy level for ultrasmall Fe_3_O_4_ NPs affords sufficient electronic states for photonic resonance, greatly boosting the matter-light interaction and facilitating more electrons participating in the Raman enhancement activity. Moreover, the DFT results indicate that the band gap of Fe_3_O_4_ NPs is narrower than that of Fe_2_O_3_ NPs because of the upshifted VB caused by the multiple crystal field energy levels (Fig. 3d). The narrow band gap of ultrasmall Fe_3_O_4_ NPs can significantly promote vibronic coupling in PICT and exciton resonance systems, increasing the charge transfer possibilities via borrowing intensity from neighboring exciton resonance, further contributing to the ultrasensitive SERS activity.

### 3.4 SERS bioprobe utilized in circulating tumor cell (CTC) detection

**Fig. 4:**
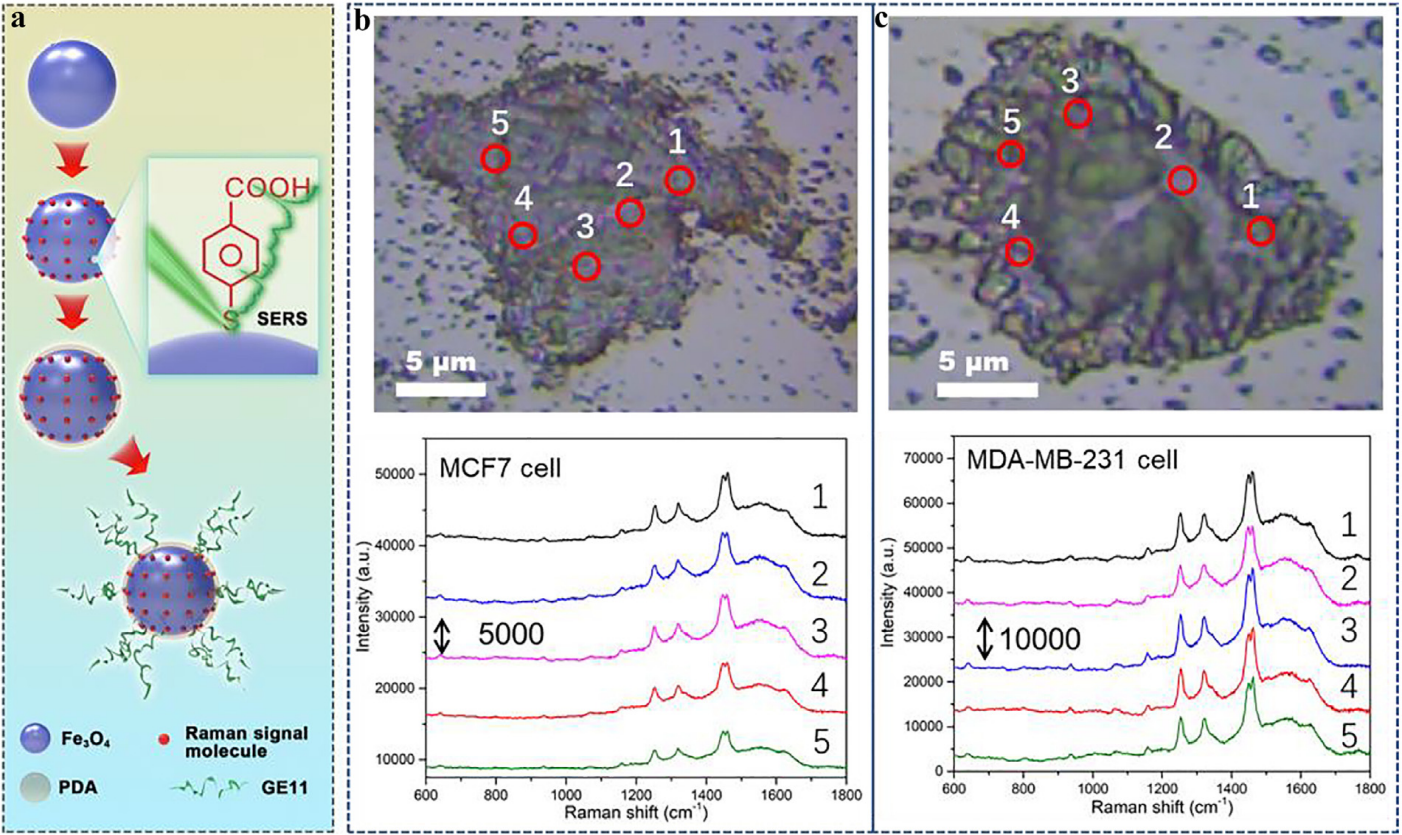
(a) Schematic of the synthetic preparation process for Fe_3_O_4_–AR–PDA–GE11 SERS bioprobe. SERS spectra of Fe_3_O_4_–AR–PDA–GE11 bioprobe collected from 5 different laser spots in rabbit blood samples with (b) MCF7 and (c) MDA-MB-231 TNB cancer cell, respectively. Laser wavelength: 532 nm; laser power: 0.2 mW; lens: 50 × objective; acquisition time: 1 s.

Ultrahigh SERS activity, excellent biocompatibility, good anti-interference ability, and selective SERS enhancement are expected to give ultrasmall Fe_3_O_4_ NPs special advantages in cancer detection and oncological imaging, especially for CTC detection and cancer cell imaging based on nanoscale spatial resolution. CTCs serve as an important indicator in early cancer screening and play a significant role in postoperative evaluation [56]. However, rapid and accurate detection of rare CTCs in peripheral blood remains a huge challenge. Fortunately, ultrasmall Fe_3_O_4_ NPs SERS platform with adsorbed Raman signal molecules may yield a highly sensitive and specific fingerprint spectrum, satisfying the detection requirements of extremely rare CTCs. To improve the specificity of Fe_3_O_4_ SERS spectra for CTC detection, two key adjustments that need to be considered are optimizing the water solubility and targeting ability of Fe_3_O_4_ NP-based SERS bioprobe. Alizarin red (AR) Raman signal molecule, polydopamine (PDA), and polypeptide GE11 (amino acid sequence YHWYGYTPQNVI) were successively adsorbed onto the surface of Fe_3_O_4_ NPs; thus, the Fe_3_O_4_–AR–PDA–GE11 SERS bioprobe was successfully designed, as shown in Fig. 4a. AR was chosen as the Raman signal molecule because it adsorbs on Fe_3_O_4_ NPs through an efficient and strong chemical bond [57], which is beneficial for the stability of SERS bioprobes; and the LOD of the AR molecule on the Fe_3_O_4_ SERS substrate reached 6 × 10^−8^ M (Fig. S9). The PDA layer was coated onto Fe_3_O_4_–AR surface complex (Fig. S10), which is favorable for boosting water solubility and improving the cell enrichment capacity. Polypeptide GE11 was conjugated to the Fe_3_O_4_–AR–PDA SERS bioprobe via amide bond linkage between the carboxyl (GE11) and amino (PDA) [58] groups, which was verified by Fourier transform infrared spectroscopy (Fig. S11). Fe_3_O_4_–AR–PDA–GE11 SERS bioprobe can effectively target tumor cells with evident epidermal growth factor receptor (EGFR) expression owing to the high binding efficiency between GE11 and EGFR [59], demonstrating that this SERS bioprobe has high detection specificity for cancerous cells.

To investigate the detection capability of Fe_3_O_4_–AR–PDA–GE11 for cancer cells, MCF7 and MDA-MB-231 triple negative breast (TNB) cancer cells were added to rabbit blood, which was used for simulating the CTC environment of peripheral blood samples. Cancer cells with EGFR expression can be traced by the Fe_3_O_4_–AR–PDA–GE11 SERS signal based on the strong anti-interference capacity of the Raman signal in the biological environment. SERS spectra measurements were carried out to identify MCF7 and MDA-MB-231 TNB cancer cells in peripheral blood samples. A significant Raman signature of the AR molecule was obtained from the SERS bioprobe targeted at CTCs. Raman vibration modes (1255 cm^−1^: C = O stretching, 1,325 cm^−1^: CC group stretching, 1,450/1,465 cm^−1^: combinations of CC, CH, and CO stretching) [60] were observed even with single MCF7 and MDA-MB-231 TNB cancer cells in 2 mL rabbit blood (Fig. 4b–c). The SERS spectra of the Fe_3_O_4_–AR–PDA–GE11 bioprobe exhibited high homogeneity and uniformity for CTC detection, whereas no Raman signal was detected in the blood sample without cancer cells (Fig. S12). The Fe_3_O_4_–AR–PDA–GE11 SERS bioprobe exhibited outstanding detection specificity and sensitivity for cancer cells with EGFR expression and semiconductor-based SERS bioprobe detection of CTC, which can be successfully deployed as an optimized and efficient bioprobe for early tumor diagnosis and postoperative monitoring.

### 3.5 High-resolution SERS imaging for cancer cells

**Fig. 5:**
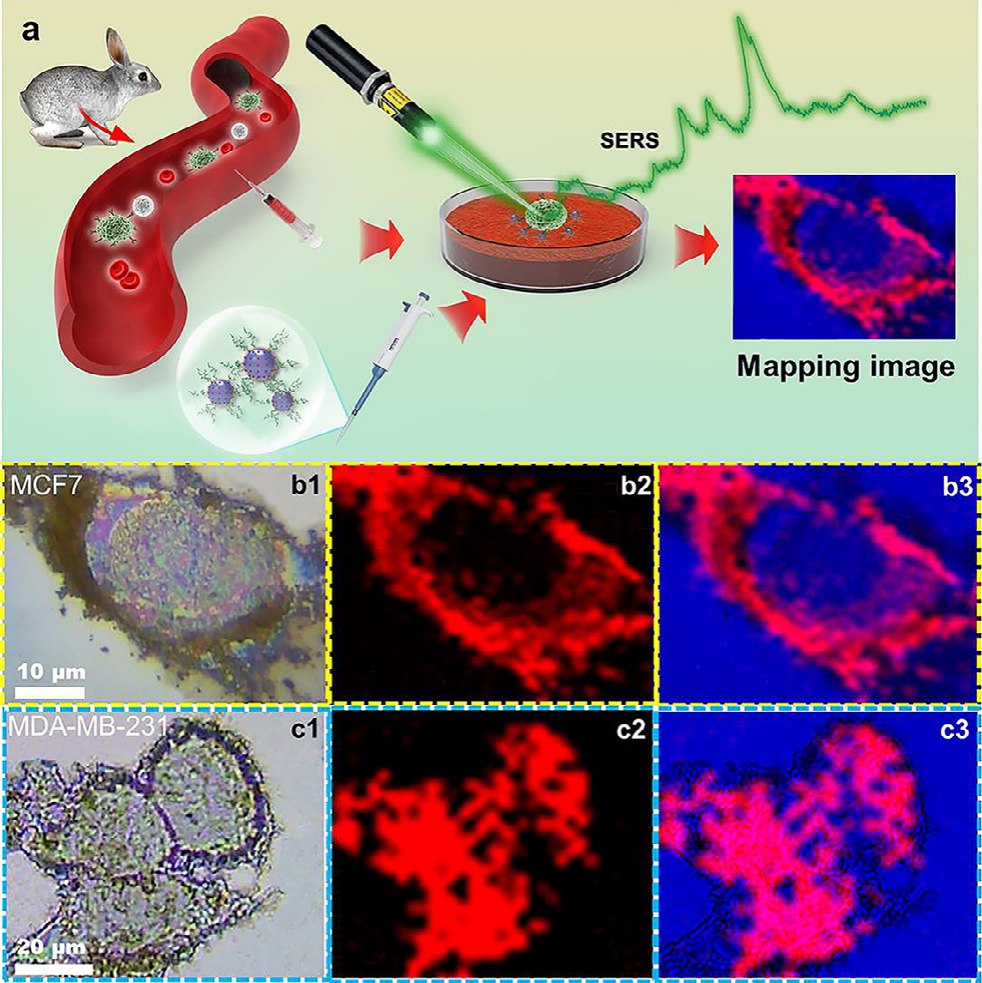
(a) Schematic diagram of SERS mapping image differentiating cancer cells in rabbit blood sample. (b1, c1) Optical microscope images, (b2, c2) SERS images, and (b3, c3) overlapped optical and SERS images of Fe_3_O_4_–AR–PDA–GE11 SERS bioprobes distributed in MCF7 and MDA-MB-231 TNB cancer cells, respectively. Laser wavelength: 532 nm; laser power: 0.1 mW; and lens: 50 × objective.

Encouraged by the excellent SERS sensitivity, high detection specificity, and good spectral uniformity of the Fe_3_O_4_-AR-PDA-GE11 SERS bioprobe, the SERS imaging abilities for distinguishing subtypes of breast cancer cells were explored. To evaluate the superiority of the Fe_3_O_4_-based optical SERS mapping images, oncological cell imaging experiments were performed on MCF7 and MDA-MB-231 TNB cancer cells. Although these two breast cancer cell types in rabbit blood were successfully detected via Raman signals, the subtypes of cancerous cells could not be directly distinguished. Classifying the subtypes of tumors plays a vital role in the accurate diagnosis and guidance of cancer treatment, thus providing a direct method to quickly differentiate subtypes of breast cancer cells, which is of great clinical value. Fe_3_O_4_–AR–PDA–GE11 SERS bioprobes were incubated with the two cancer cell lines in processed rabbit blood samples. The two cancer cells were quickly and clearly distinguished using high-resolution SERS mapping images, as described in the schematic (Fig. 5a). MDA-MB-231 TNB cancer cells have high EGFR expression, while that of MCF7 cancer cells is relatively low [61], [62], which predominantly affected the cellular uptake of the Fe_3_O_4_–AR–PDA–GE11 SERS bioprobe. This cellular uptake feature can be quickly and intuitively identified by tracing the distribution of the Fe_3_O_4_–AR–PDA–GE11 bioprobes on SERS optical mapping images. SERS imaging results demonstrated that significantly more EGFR expression-related uptake of Fe_3_O_4_-based bioprobes occurred in MDA-MB-231 TNB cancer cells than in MCF7 cells, as shown in Fig. 5b, c. High-resolution SERS imaging was performed by analyzing 1255 cm^−1^ (C=O vibration stretching) Raman modes of AR molecules, and the results exhibited a high degree of agreement with the optical image of cancer cells.

A larger amount of SERS bioprobe uptake by MDA-MB-231 TNB cancer cells induced a more intense SERS signal than in MCF7 cancer cells (Fig. 4b–c). SERS optical images (Fig. 5b–c) of the two subtypes of breast tumor cells were highly consistent with the SERS signal intensity results. SERS mapping images clearly show that a large number of Fe_3_O_4_–AR–PDA–GE11 SERS bioprobes is distributed in different regions of MDA-MB-231 TNB cancer cells owing to the higher EGFR expression, while SERS bioprobes mainly accumulated around MCF7 cancer cells through nonspecific adsorption. The targeting ability of SERS bioprobes co-incubated with MCF7 and MDA-MB-231 TNB cancer cells was further confirmed by immunofluorescence staining, as shown in Fig. S13. Significantly more SERS bioprobes were present in MDA-MB-231 TNB cancer cells, as observed by laser scanning confocal microscopy, which is in good agreement with the SERS imaging results, and offers sufficient evidence to support the SERS imaging capability of the bioprobe to quickly differentiate the two breast cancer cell lines. As shown by inductively coupled plasma optical emission spectrometry (Fig. S14), the quantity of Fe_3_O_4_–AR–PDA–GE11 SERS bioprobes taken up by MDA-MB-231 TNB cancer cells was approximately 2.3 times greater than that by MCF7 cancer cells after 3 h of co-incubation, verifying that Fe_3_O_4_-based SERS bioprobes can accurately identify tumor subtypes with EGFR expression. In addition, high-resolution SERS images enable direct observation of the quantity distribution and behavior of Fe_3_O_4_-based bioprobes at the cellular level and offer a novel approach for studying the interactions between NPs and cells. SERS microimaging derived from Raman vibration modes showed excellent performance in distinguishing different subtypes of cancer cells *in vitro*, satisfying the requirements of accurately diagnosing cancer cells via liquid biopsy.

### 3.6 SERS–MRI dual-modal cancer imaging

**Fig. 6:**
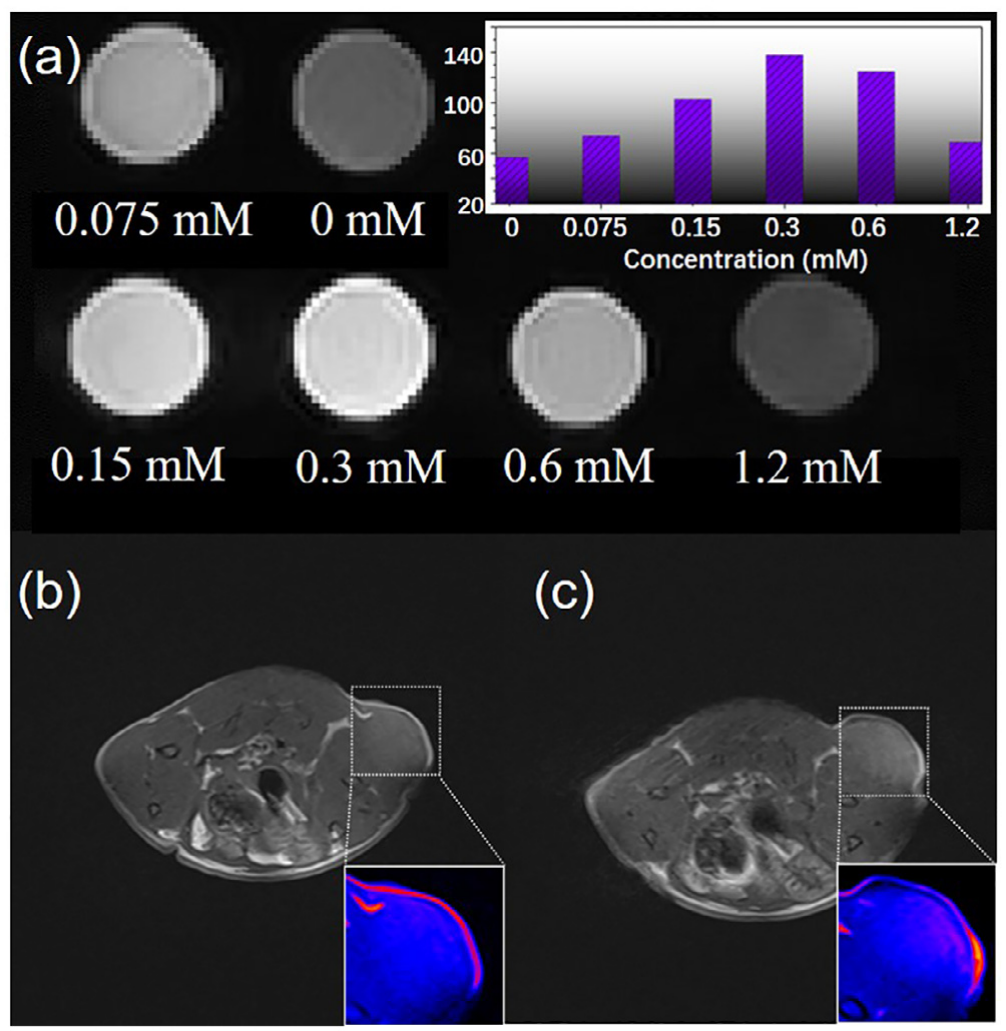
(a) *T_1_*-weighted MR images of Fe_3_O_4_–AR–PDA–GE11 bioprobes at different concentrations *in vitro*. (b, c) *T_1_*-weighted MR images and the color-coded images of MDA-MB-231 TNB tumor-bearing nude mice before and 50 min after intravenous injection of Fe_3_O_4_–AR–PDA–GE11 bioprobes, respectively.

The development of a novel bioprobe with dual-modal imaging capability is of great significance in the early screening and treatment of tumors. The Fe_3_O_4_-based bioprobes proposed in this study exhibited excellent SERS activity in oncological imaging at the cellular level *in vitro* and can also serve as a high-potential contrast agent for *T*_1_-weighted magnetic resonance imaging (MRI), realizing active-targeted imaging of tumor tissues *in vivo*. Benefiting from the abundant unpaired electrons of Fe^3+^ and the decreased spin-canted proportion derived from the reduced diameter of Fe_3_O_4_ NPs, ultrasmall Fe_3_O_4_-based bioprobes exhibited remarkable MRI contrast agent activity. The superior *T_1_*-weighted MRI contrast enhancement of ultrasmall Fe_3_O_4_-based bioprobes can also be explained by the theory of inner spheres and outer spheres [6]. Concentration-dependent *T*_1_-weighted MRI measurements were carried out *in vitro* using a 0.47 T MRI instrument (*r*_2_/*r*_1_ = 3.26), as shown in Fig. 6a. The Fe_3_O_4_-based bioprobes exhibited the best contrast enhancement at 0.3 mM. To investigate the applicability of the Fe_3_O_4_–AR–PDA–GE11 bioprobes in MRI *in vivo*, a subcutaneous MDA-MB-231 TNB tumor model was established in Balb/C nude mice. A significant *T*_1_-weighted MRI signal was observed when the tumor-bearing mice were injected with Fe_3_O_4_-based bioprobes (75 µL, 1 mg/mL) via tail vein injection. MRI of the tumor *in vivo* was performed using a 3.0 T human clinical scanner after 50 min intravenous injection (Fig. 6b–c). In addition, damage to the main organs was not detected in the test mice 14 days post-injection of Fe_3_O_4_-based bioprobes, as shown in the H&E staining images (Fig. S15), indicating that the bioprobes possess appreciable histocompatibility and minimal toxicity to normal organs. Therefore, the rational design of SERS–MRI dual-modal imaging bioprobes provided complementary cancer imaging information from the cell to the tissue level, which could hold great promise for image-guided tumor diagnosis *in vivo* and *in vitro*.

## 4 Conclusion

A novel semiconductor nanomaterial, that is, ultrasmall Fe_3_O_4_ NPs, showing remarkable SERS activity of 9.06 × 10^3^ EF and 5 × 10^−9^ M LOD for CV molecule was designed and prepared. In the fabricated material, high-efficiency interfacial PICT was promoted by multiple electronic energy levels derived from the multiple valence states of Fe, as observed by UV–vis diffuse reflectance spectroscopy. Multiple electronic energy levels offer sufficient electron transition routes for interfacial charge transfer in ultra-small Fe_3_O_4_ SERS systems. DFT calculations indicate that the narrow band gap and high electronic DOS favor the establishment of a stable molecule@Fe_3_O_4_ SERS system with a strong vibronic coupling resonance. The above-mentioned factors greatly increased the molecular polarizability tensor and amplified the molecular Raman cross-section. CTCs were readily recognized using high-sensitivity SERS spectra, and subtypes of breast cancer cells were accurately distinguished through high-resolution SERS imaging, which is of great significance for tumor classification *in vitro*. Moreover, pure Fe_3_O_4_-based bioprobes were used as a *T_1_* MRI contrast agent for tumor imaging *in vivo*, enabling highly efficient tumor diagnosis based on SERS–MRI dual-modal imaging ranging from cell to tissue levels. This optimized SERS–MRI dual-modal nanoprobes can be extensively utilized for early tumor diagnosis *in vivo* and *in vitro* and have great application potential for image-guided tumor treatment.

## Declaration of competing interest

The authors declare that they have no conflicts of interest in this work.

## Appendix Supplementary materials

Image, application 1

Image, application 2
