# Supplementary material for: Multiple valence states of Fe boosting SERS activity of Fe3O4 nanoparticles and enabling effective SERS-MRI bimodal cancer imaging
Source: Fundam Res. 2022 May 3;4(4):858–67. doi: 10.1016/j.fmre.2022.04.018 (PMC11330100; doi:10.1016/j.fmre.2022.04.018)
Supplement: Supplementary file 1 [file mmc1.docx]

**Supporting Information**

**Multiple Valence States of Fe Boosting SERS Activity of Fe_3_O_4_ Nanoparticles and Enabling Effective SERS-MRI** **Bimodal Cancer Imaging**

Jie Lin ^a,^*^,1^, Xuehua Ma ^a,c,1^, Anran Li ^b,1^, Ozioma Udochukwu Akakuru^a^, Chunshu Pan^a^, Meng He^a^, Chenyang Yao^a^, Wenzhi Ren^a^, Yanying Li^a^, Dinghu Zhang^a^, Yi Cao^a^, Tianxiang Chen ^a,^* and Aiguo Wu ^a,^*

^a^Cixi Institute of Biomedical Engineering, International Cooperation Base of Biomedical Materials Technology and Application, Chinese Academy of Science (CAS) Key Laboratory of Magnetic Materials and Devices, Zhejiang Engineering Research Center for Biomedical Materials, Ningbo Institute of Materials Technology and Engineering, CAS, 1219 Zhongguan West Road, Ningbo 315201, P. R. China. Advanced Energy Science and Technology Guangdong Laboratory, Huizhou 516000, P.R. China

^b^School of Engineering Medicine, Beihang University, Beijing, 100191, China; and Key Laboratory of Big Data-Based Precision Medicine (Beihang University), Ministry of Industry and Information Technology.

^c^University of Chinese Academy of Sciences, Beijing 100049, P. R. China.

*Corresponding authors.

E-mail addresses: linjie@nimte.ac.cn (J. Lin), chentx@nimte.ac.cn (T. Chen) aiguo@nimte.ac.cn (A. Wu)

^1^These authors contributed equally to this work.





Figure S1. TEM image of ultrasmall Fe_3_O_4_ NPs.


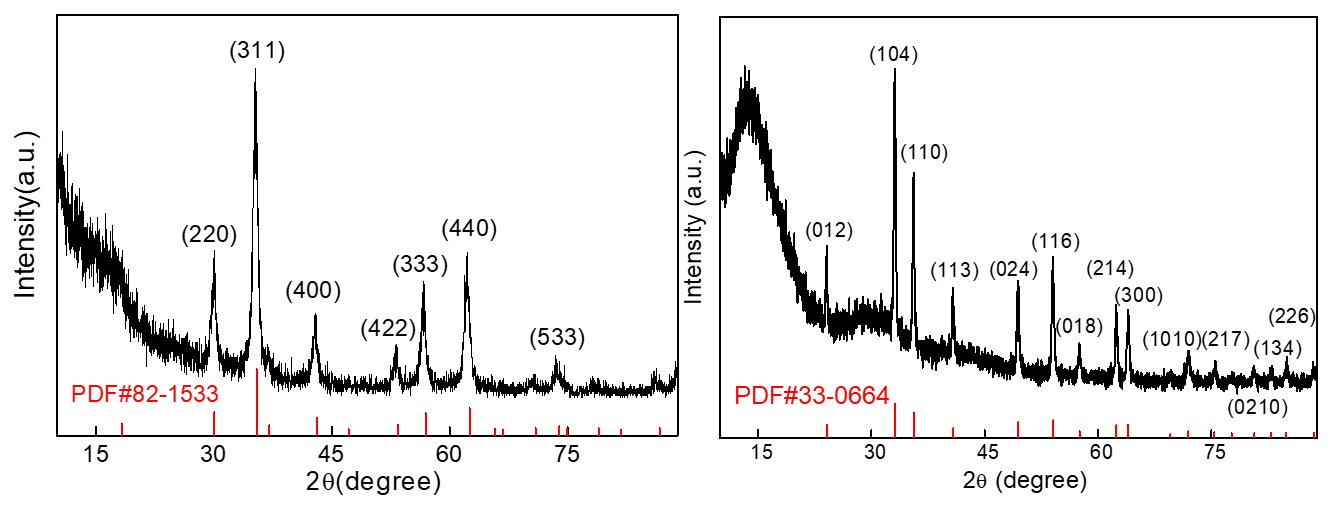


Figure S2. XRD characterizations of ultrasmall Fe_3_O_4_ and α-Fe_2_O_3_ NPs.


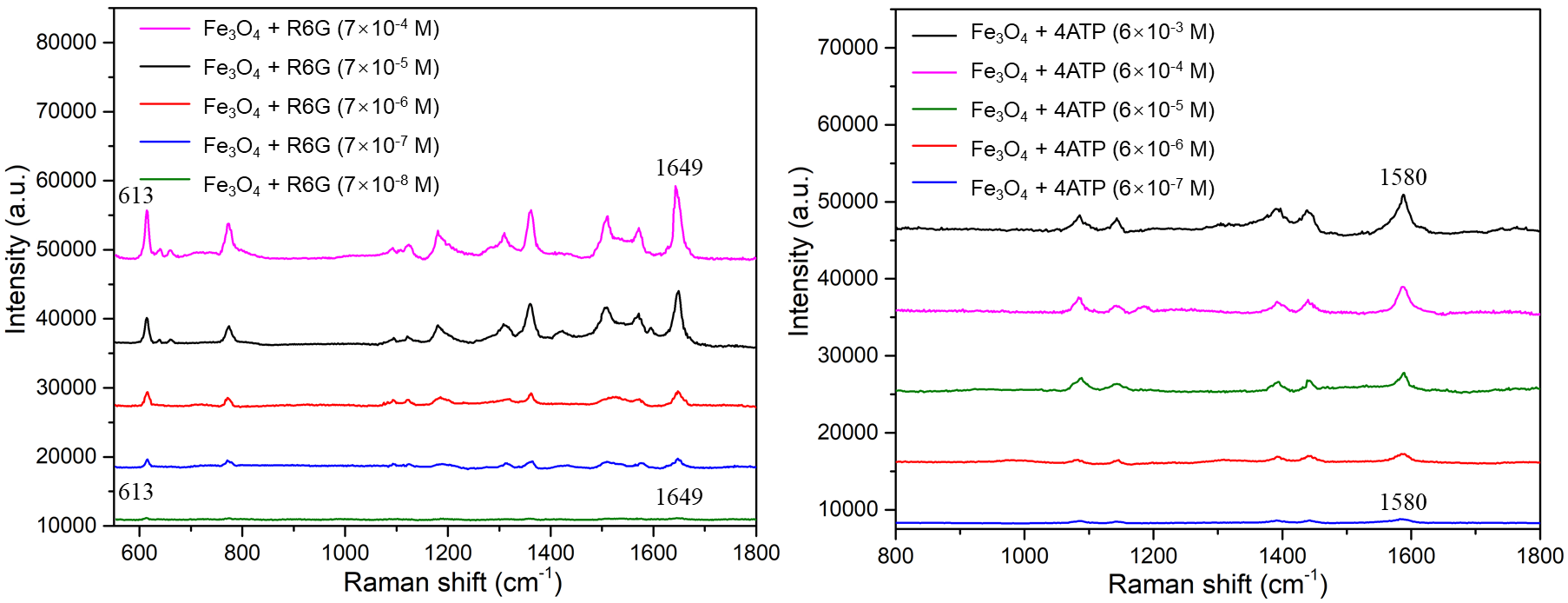


Figure S3. SERS spectra of different concentrations of R6G and 4ATP probe molecules adsorbed on Fe_3_O_4_ NPs. Laser wavelength: 532 nm; laser power: 0.5 mW; lens: 50 × objective; and acquisition time: 1 s.


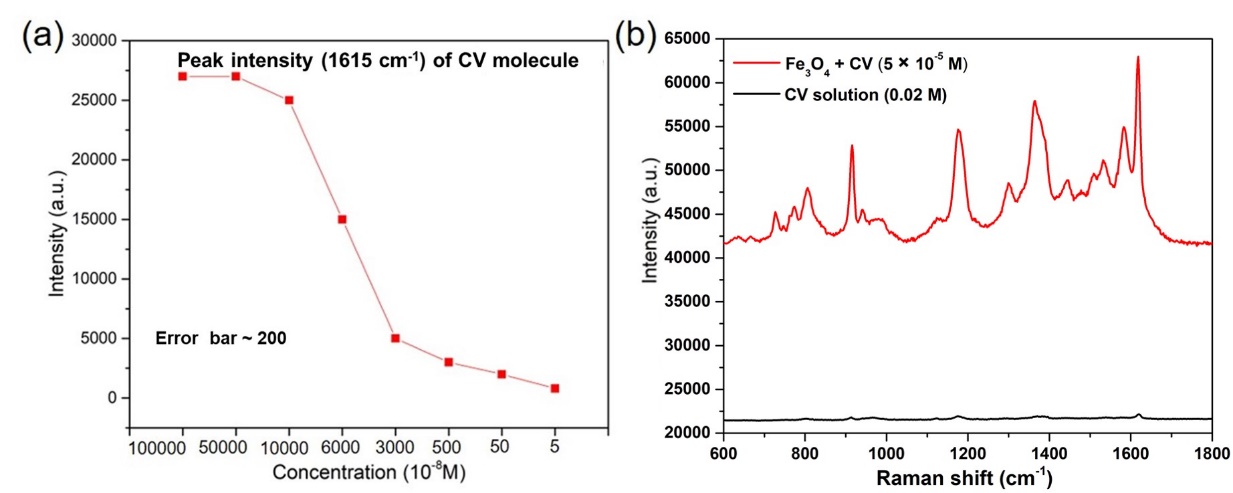


Figure S4. (a) SERS peak intensity (1615 cm^-1^) of CV molecule adsorbed on Fe_3_O_4_ NPs at different concentrations. (b) The vibration peak intensity comparison between SERS spectra (Integration time: 1 s) and non-SERS solution spectra (Integration time: 2 s) of CV molecule.


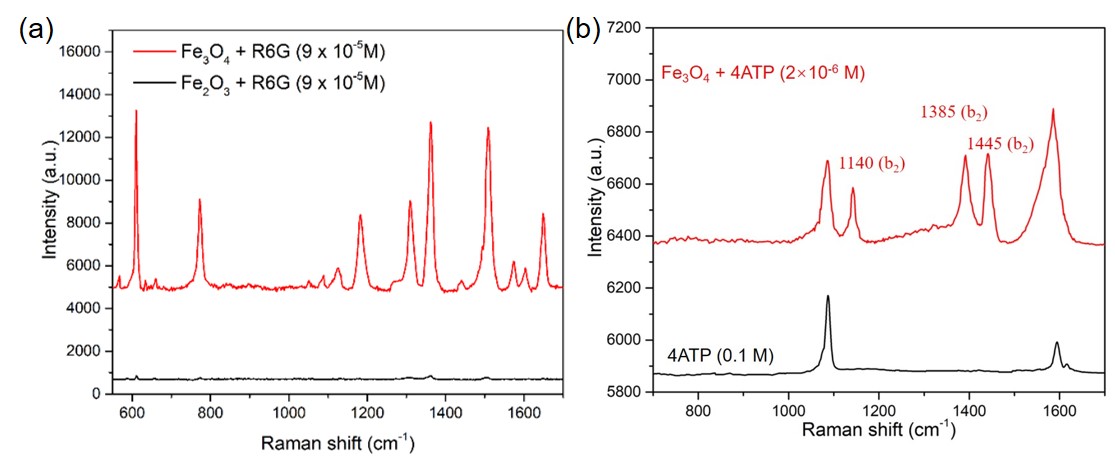


Figure S5. (a). SERS spectra comparison of R6G molecules adsorbed on Fe_3_O_4_ and Fe_2_O_3_ NPs, respectively. (b) SERS spectra of 4ATP molecules adsorbed on Fe_3_O_4_ NPs, and Raman spectra of pure 4ATP molecules. Laser wavelength: 532 nm; laser power: 0.5 mW; lens: 50 × objective; and acquisition time: 2 s.


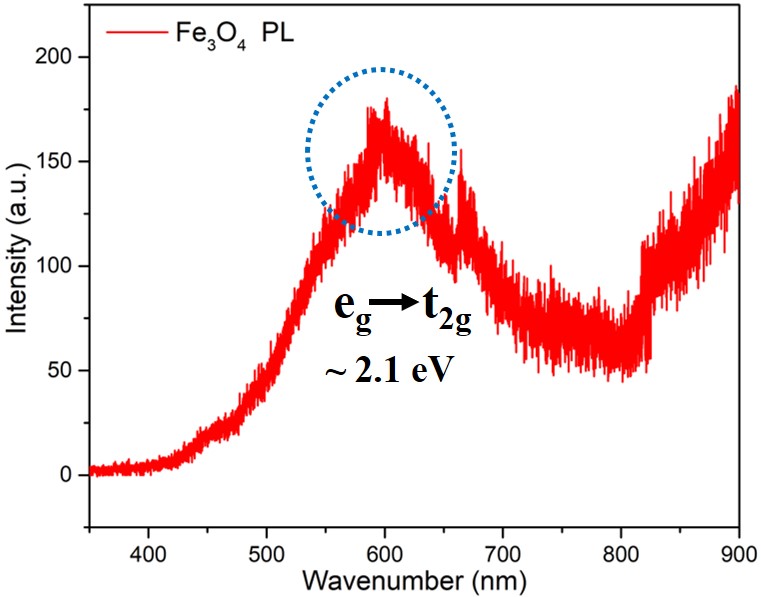


Figure S6. Photoluminescence (PL) spectroscopy of Fe_3_O_4_ NPs, ~ 590 nm peak is produced by radiative recombination of electrons transferred from e_g_ to t_2g_ crystal field band of octahedral site (Fe^3+^ and Fe^2+^).


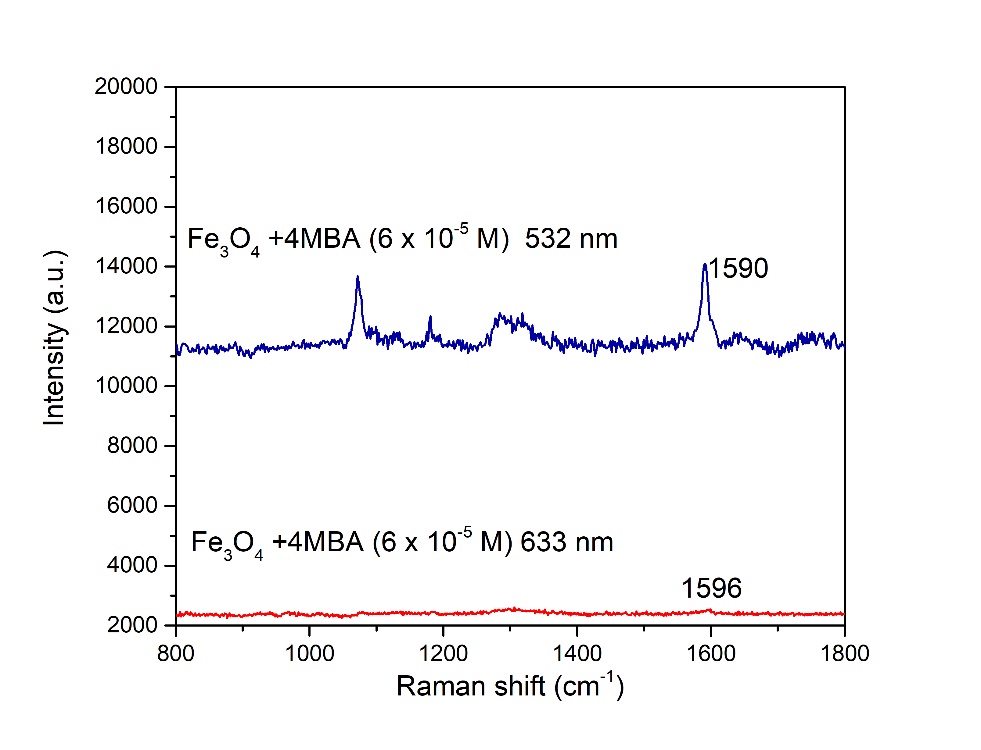


Figure S7. SERS spectra of 4MBA molecule (6 × 10^-5^ M) absorbed on Fe_3_O_4_ SERS substrate under different laser illuminations. Laser wavelength: 532 nm, 633nm; laser power: 0.5 mW; lens: 50 × objective; and acquisition time: 1 s.


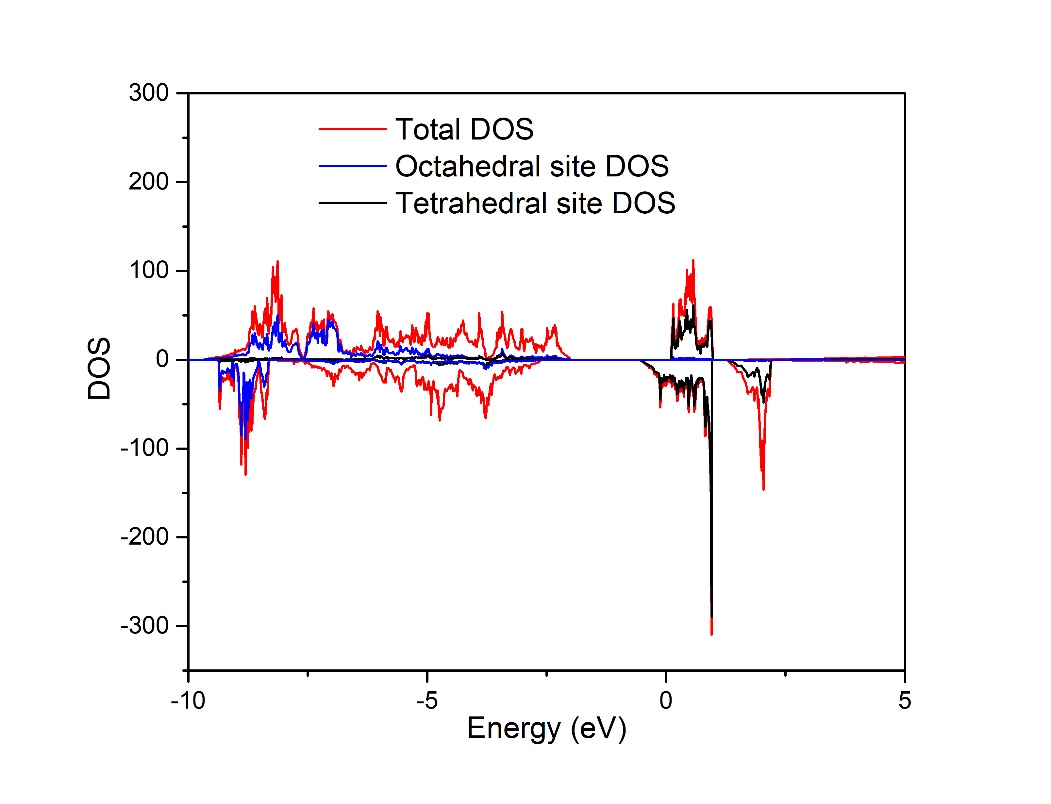


Figure S8. Total DOS, Fe ions exist on the octahedral, and tetrahedral sites DOS of ultrasmall Fe_3_O_4_ NPs.


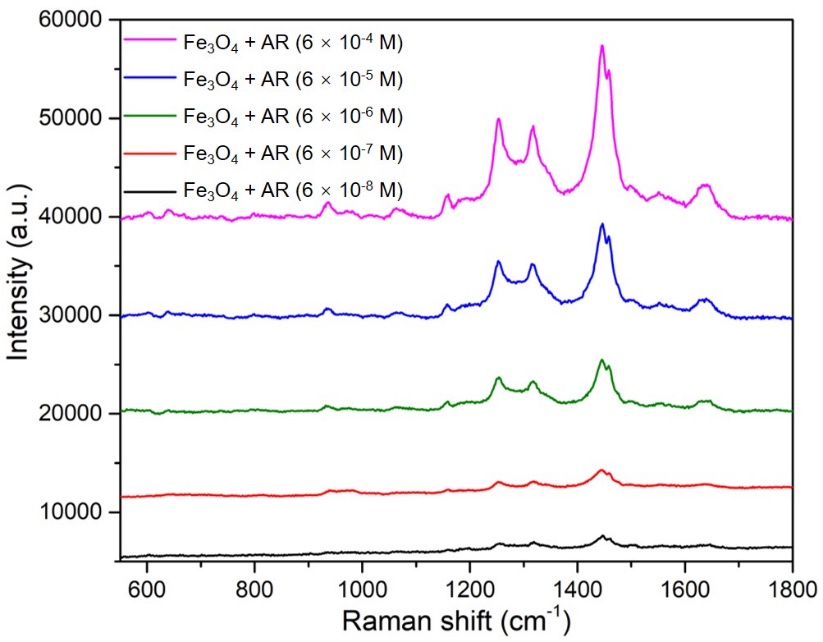


Figure S9. SERS spectra of AR molecule adsorbed on Fe_3_O_4_ NPs at different concentrations. Laser wavelength: 532 nm; laser power: 0.5 mW; lens: 50 × objective; and acquisition time: 1 s.


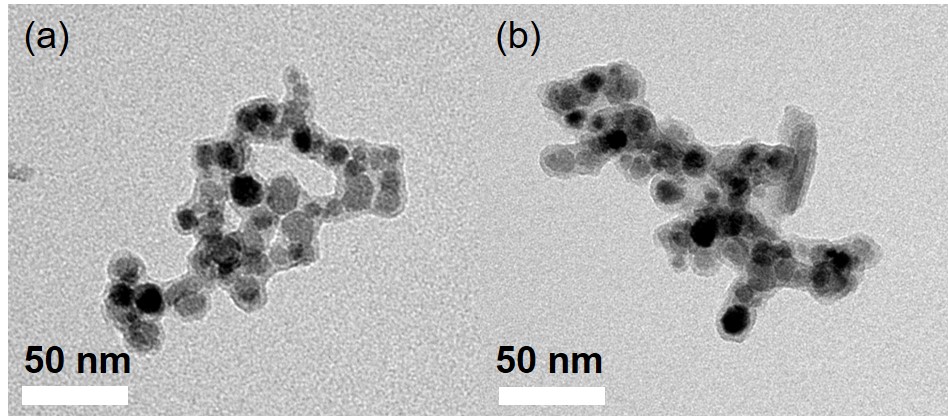


Figure S10. TEM images of Fe_3_O_4_-AR-PDA SERS bioprobes.


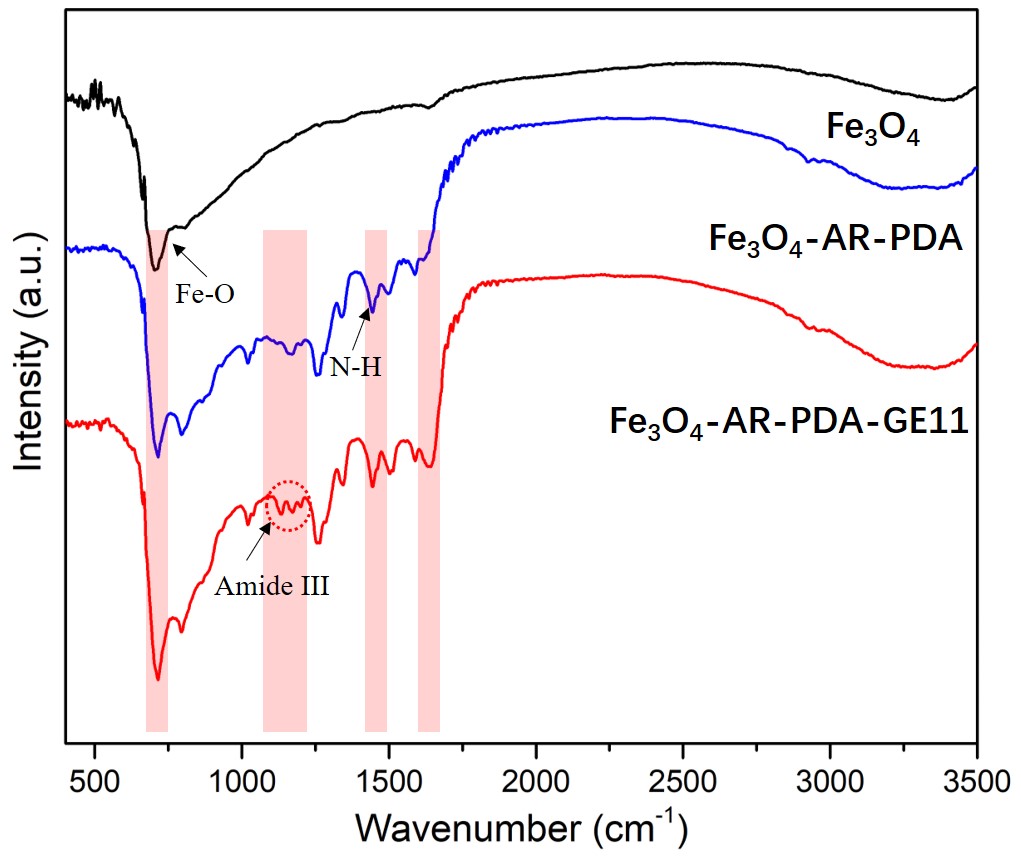


Figure S11. Fourier transform infrared spectra of Fe_3_O_4_, Fe_3_O_4_-AR-PDA, and Fe_3_O_4_-AR-PDA -GE11 SERS bioprobe.


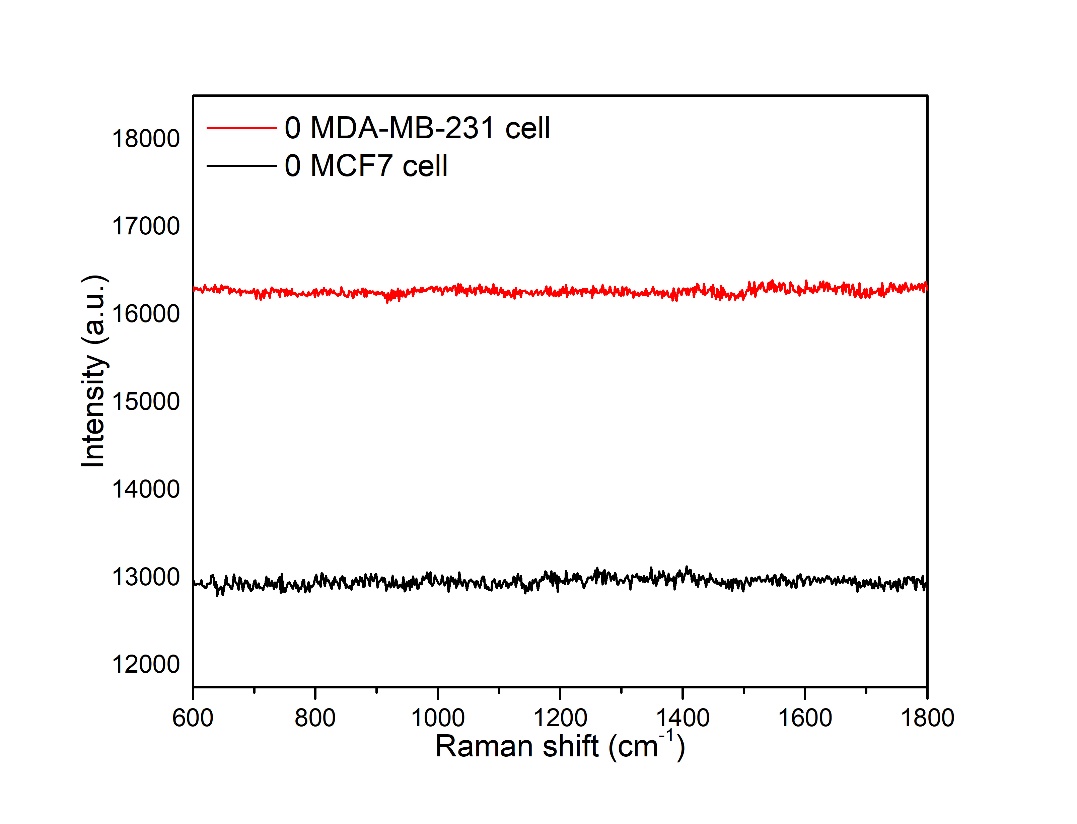


Figure S12. SERS signals of Fe_3_O_4_-AR-PDA-GE11 bioprobes without MCF7 and MDA-MB-231 cancer cell in rabbit blood. Laser wavelength: 532 nm; laser power: 0.2 mW; lens: 50 × objective; and acquisition time: 1 s.


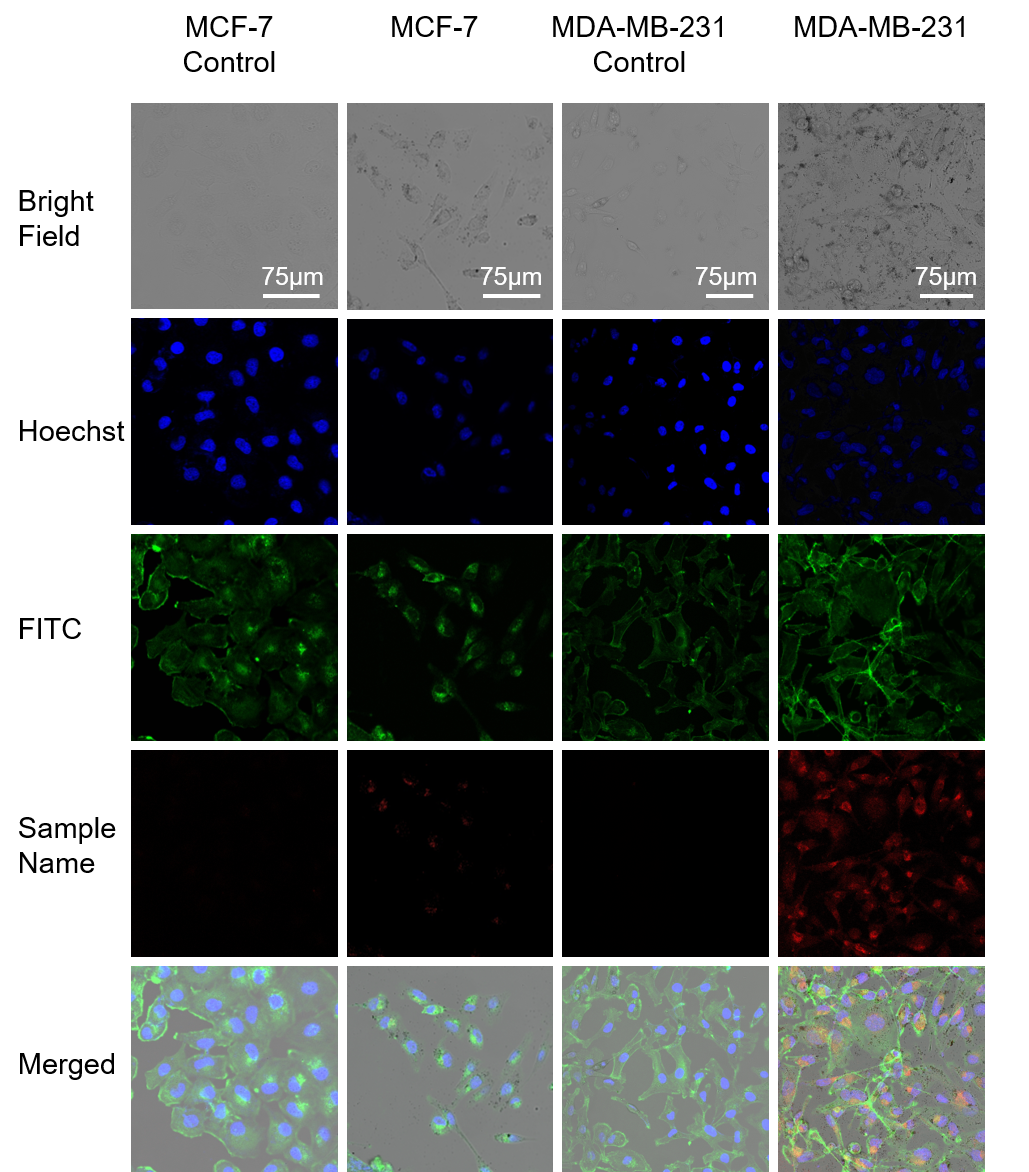


Figure S13. Laser scanning confocal microscope (LSCM) images of MCF-7 and MDA-MB-231 cancer cells co-incubated with Fe3O4-AR-PDA-GE11 SERS bioprobe for 4 h at 37 °C. The samples were simultaneously excited at 405, 488, and 552 nm. The cytoskeletons stained with FITC phalloidin are green (EM 600-680 nm) at an excitation of 552 nm, and the nuclei stained with Hoechst (EM 415-485 nm) are blue at an excitation of 405 nm. The AR are red (EM 506-576 nm) at an excitation of 488nm.


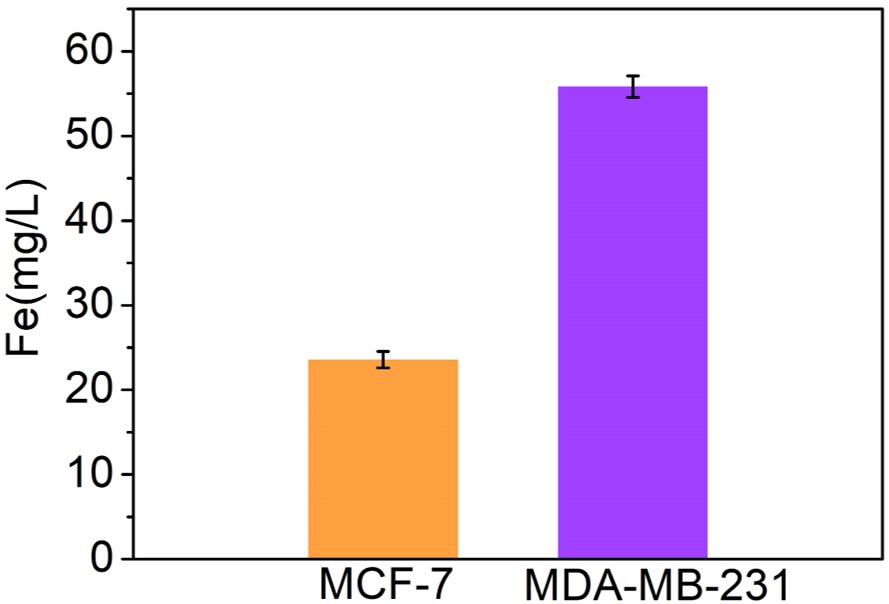


Figure S14. ICP-OES measurements of 1 mL Fe_3_O_4_-AR-PDA-GE11 SERS bioprobes (150 μg/mL) incubated with 400000 MCF-7, and MDA-MB-231 cancerous cells for 3 h.


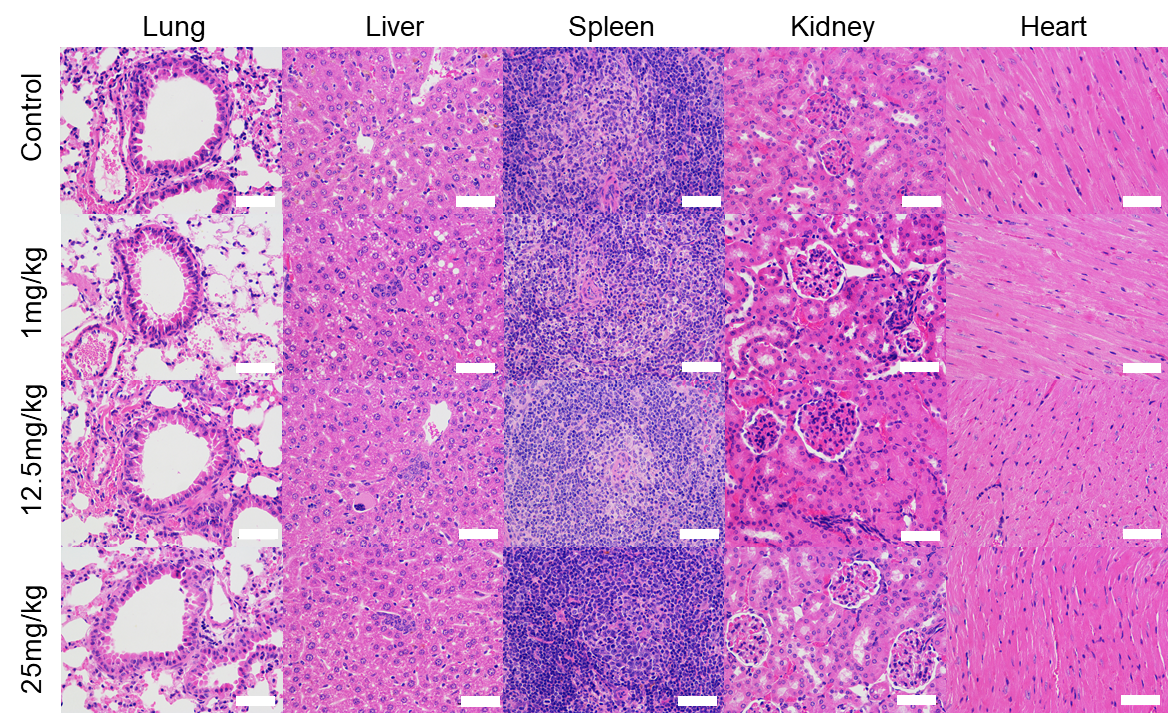


Figure S15. H&E staining of major organs collected from MDA-MB-231 TNB tumor-bearing mice after bioprobes intravenously injection for 14 days. (Scale bar = 50 μm)
