# Supplementary material for: Multiple valence states of Fe boosting SERS activity of Fe3O4 nanoparticles and enabling effective SERS-MRI bimodal cancer imaging
Source: Fundam Res. 2022 May 3;4(4):858–67. doi: 10.1016/j.fmre.2022.04.018 (PMC11330100; doi:10.1016/j.fmre.2022.04.018)
Supplement: Supplementary file 2 [file mmc2.pdf]

Multiple Valence States of Fe Boosting SERS Activity of Fe_3O_4 Nanoparticles and Enabling Effective SERS-MRI Bimodal Cancer Imaging

Jie Lin [a,*,1], Xuehua Ma [a,c,1], Anran Li [b,1], Ozioma Udochukwu Akakuru[a], Chunshu Pan[a], Meng He[a], Chenyang Yao[a], Wenzhi Ren[a], Yanying Li[a], Dinghu Zhang[a], Yi Cao[a], Tianxiang Chen [a,*] and Aiguo Wu [a,*]

[a]Cixi Institute of Biomedical Engineering, International Cooperation Base of Biomedical Materials Technology and Application, Chinese Academy of Science (CAS) Key Laboratory of Magnetic Materials and Devices, Zhejiang Engineering Research Center for Biomedical Materials, Ningbo Institute of Materials Technology and Engineering, CAS, 1219 Zhongguan West Road, Ningbo 315201, P. R. China. Advanced Energy Science and Technology Guangdong Laboratory, Huizhou 516000, P.R. China

[b]School of Engineering Medicine, Beihang University, Beijing, 100191, China; and Key Laboratory of Big Data-Based Precision Medicine (Beihang University), Ministry of Industry and Information Technology.

[c]University of Chinese Academy of Sciences, Beijing 100049, P. R. China.

*Corresponding authors.

E-mail addresses: linjie@nimte.ac.cn (J. Lin), chentx@nimte.ac.cn (T. Chen) aiguo@nimte.ac.cn (A. Wu)

[1]These authors contributed equally to this work.

ABSTRACT

Developing novel nanoparticle-based bioprobe utilized in clinical setting with imaging resolution ranging from cell to tissue levels, is a major challenge for tumor diagnosis and treatment. Herein, an optimized strategy by designing Fe_3O_4-based bioprobe for dual-modal cancer imaging based on surface-enhanced Raman scattering (SERS) and magnetic resonance imaging (MRI) is introduced. Remarkable SERS activity of ultrasmall Fe_3O_4 nanoparticles (NPs) is firstly discovered, and 5×10^{-9} M limit of detection for CV molecule is successfully obtained. High-efficiency interfacial photon-induced charge transfer (PICT) process of Fe_3O_4 NPs is promoted by multiple electronic energy levels ascribed to the multiple valence states of Fe, which is carefully observed by UV-vis diffuse reflectance spectroscopy. Density functional theory calculation is utilized to further reveal that the narrow band gap and high electronic density of states of ultrasmall Fe_3O_4 NPs notably boost the vibronic coupling resonances in the SERS system upon illumination. Subtypes of cancer cells can be accurately recognized by high-resolution SERS imaging *in vitro* via constructing Fe_3O_4-based bioprobe with high sensitivity and good specificity. Significantly, Fe_3O_4-based bioprobe simultaneously exhibits T_1-weighted MRI contrast enhancement with active-targeting capability for tumors *in vivo*. As far as we know, this is the first time that pure semiconductor-based SERS-MRI bimodal nanoprobes are utilized in

tumor imaging *in vivo* and *in vitro*, which is only achieved by semiconductor-metal complex materials previously. The non-metal materials with SERS-MRI dual-modal imaging modalities established in this report serve as a promising cancer diagnostic platform, which not only show obvious superiority in early tumor diagnosis, but also possess great potential for image-guided tumor treatment.

Keywords: Fe_3O_4 NPs, SERS-MRI bimodal nanoprobes, PICT, multiple valence states, cancer diagnosis.

1. Introduction

Cancer has been considered as one of the greatest threatening diseases to human health based on the fact that nearly 10 million people die of cancer in the world every year [1]. Although the best strategies for cancer treatment is early diagnosis [2], cancer patients are often diagnosed at middle-advanced stages and usually miss the rational treatment time [3]. Magnetic resonance imaging (MRI) serves as a significant technology in disease diagnosis with the particular advantages of no harmful radiation, detecting flow field *in situ*, and multi-parameter imaging, which are beneficial to tumor treatment and surgery *in vivo* [4-6]. However, its relatively low detection sensitivity, high technical demand, and poor imaging resolution seriously impede MRI utilized in early clinical tumor diagnosis [7]. Currently, materials-based MRI contrast agents have been reported with the capability of improving imaging resolution based on their unique physicochemical properties and tumor-targeting features [8], which partly expand the scope of MRI in tumor diagnostic and therapeutic fields. Although limited by spatiotemporal resolution in early cancer screening, MRI contrast agents play an irreplaceable role in enhancing signal contrasts in tissues of interest [9]. Optical imaging modalities possess the feature of micron-scale spatial resolution and non-harmful radiation, providing cellular level imaging resolution for tumor, and largely boosting successful rate of tumor preoperative diagnosis [10,11]. Fluorescence

spectroscopy exhibits high sensitivity and good anti-interference ability as oncological imaging modality, however, tissue autofluorescence and photobleaching hinder its practical application [12]. Recently, surface-enhanced Raman scattering (SERS) has emerged as a promising technique utilized in cancer biosensing and clinically relevant research area [13-16]. SERS imaging offers the advantages of nondestructive detection, ultrahigh sensitivity, selective enhancement, label-free analysis, nanoscale spatial resolution, and provides molecular fingerprint vibrational information [17-20]. Hence, SERS imaging has attracted great interest in tumor components identification, circulating tumor cells detection, precise delineation of tumor margins, and drug delivery monitoring [11,21-24].

SERS technology can offer deep insights with detailed structural information through point-to-point spectra and mapping image based on the nano-micro material detection schemes [25]. Noble metal materials with ultrahigh SERS enhancement factors (EFs) have been widely utilized in various detection and analysis fields due to their huge interfacial electromagnetic field produced by surface plasmon resonance (SPR) effect under laser illumination [17,26]. Semiconductor materials-based SERS platforms derived from chemical enhancement mechanism (CM) are also widely applied in the field of biological imaging and detection [27-29]. Semiconductor SERS platform is recognized as a promising analytical tool in cancer diagnosis and precision medicine due to their good

biocompatibility, excellent spectral stability, selective SERS enhancement, and fingerprint vibration modes [30,31], which endow them with unique superiority in SERS imaging for tumor detection. Much less attention has been paid to semiconductor SERS substrates due to their relatively weak SERS EF and limited SERS optical modality for large-scale tumor tissue imaging. Fortunately, several semiconductor materials have been reported with metal-comparable SERS EF based on the controllable synthetic strategy, for instance, surface defect engineering [32,33], constructing amorphous phase [34], n-/p- type element doping [35,36], crystal facet regulation [37], and designing two-dimensional (2D) nanomaterials [38]. These results indicate photon-induced charge transfer (PICT) efficiency in semiconductor SERS system can be greatly improved via modifying surface physicochemical electronic structure, which is the key point for boosting interfacial electron transfer between SERS substrate and molecules [35]. Hence, exploring novel semiconductor nanomaterials with unique surface electronic structures is beneficial to establishing efficient interfacial electron transport channel, obtaining high-efficiency PICT, and magnifying target molecular polarization tensor, which satisfies high-sensitive SERS detection and imaging modes. SERS imaging for tumor tissues is limited in the nano/micro scale region due to point-by-point acquisition of laser spot, which greatly inhibits its further application in tumor imaging. The shortness of SERS imaging method utilized in tumor

tissues can be greatly augmented by MRI mode due to the exciting discoveries that several semiconductor materials have been developed as MRI contrast agents [39,40], presenting the exciting possibilities of SERS-MRI bimodal imaging modalities. The dual-modal imaging nanoprobes will therefore be expected to exhibit prospective imaging capability for cancer diagnosis and treatment ranging from cell to tissue level.

Motivated by the above analysis and discussion, novel semiconductor-based nanoprobes with SERS-MRI dual-modal imaging modalities are successfully designed. Ultrasmall Fe_3O_4 nanoparticles (NPs) exhibit good SERS sensitivity with 9.06×10^3 EF, and 5×10^{-9} M limit of detection for CV molecule, possessing stronger SERS activity than that of Fe_2O_3 NPs counterparts. Multiple electronic energy levels derived from multiple valence states of Fe play a vital role for improving interfacial charge transfer process in the Fe_3O_4 SERS system, which is revealed by UV-vis diffuse reflectance spectroscopy. The DFT calculation indicates Fe_3O_4 NPs possess narrow band gap and high electronic density of states (DOS), which are beneficial to forming stable ultrasmall Fe_3O_4-molecule SERS system and generating strong vibronic coupling resonance. The above-mentioned factors synergistically endow ultrasmall Fe_3O_4 NPs with an efficient PICT process, which magnifies molecular polarization tensor and results in strongly enhanced Raman signal. Circulating tumor cell is effectively detected by the Fe_3O_4-based SERS bioprobe, and subtypes of

breast tumor cell can be quickly distinguished via high-resolution SERS imaging. Significantly, the Fe_3O_4-based bioprobe can serve as an ideal T_1-weighted MRI contrast agent for tumor imaging in mice with active-targeting capability *in vivo*, thus achieving dual-modal SERS-MRI imaging modalities ranging from cell to tissue level. The Fe_3O_4-based SERS-MRI bioprobe provides a new pathway for accurately recognizing different subtypes of cancer cells, and providing detailed oncological imaging information *in vivo* and *in vitro*, greatly improves the success rate of early cancer diagnosis. The reported MRI-SERS bimodal bioprobe is accomplished by Fe_3O_4-metal[41-42], and Fe_3O_4-semiconductor complex[43] previously, in which Fe_3O_4 only offer MRI activity, and SERS activity of Fe_3O_4NPs are both ignored. To our best knowledge, this is the first report on pure Fe_3O_4 material with SERS-MRI bimodal activity for tumor imaging. Designing SERS-MRI dual-modal bioprobes can be regarded as an innovatory and reliable strategy for high-sensitive and label-free cancer imaging. Moreover, the bimodal nanoprobe holds huge potential of becoming a new tool for image-guided tumor treatment.

2. Results and discussion

2.1 Characterization of ultrasmall Fe_3O_4 NPs.

The efficiency of PICT process in semiconductor SERS system is strongly dependent on the electron transition paths from substrate to molecule [20,28]. Therefore, ultrahigh SERS EF may be obtained via

developing novel semiconductor substrate with multiple electronic energy levels, which serve as extra springboards to assist the PICT transitions. Hence, Fe_3O_4 nanomaterial is selected as a potential candidate to exhibit remarkable SERS activity due to the multiple valence states of Fe. Synthesis protocol of ultrasmall Fe_3O_4 NPs is based on our previous report [44] with some modifications. Ultrasmall Fe_2O_3 NPs are successfully prepared through high temperature calcination synthetic route, and serve as a control SERS substrate. Images of Fe_3O_4 and Fe_2O_3 NPs obtained by transmission electron microscopy (TEM) are shown in Fig. 1, revealing sizes of the acquired two ultrasmall samples as ~ 5 to 8 nm. A broad-view TEM image (Fig. S1) demonstrates the relatively uniform sizes and homogeneous dispersity of ultrasmall Fe_3O_4 NPs. High-resolution TEM (HRTEM) images (Fig. 1b, e) selected from the squared region (Fig. 1a, d) illustrate that the observed lattice fringes (0.252 nm, 0.297 nm, and 0.271 nm) correspond to (311), (220) crystal planes of Fe_3O_4, and (104) crystal plane of Fe_2O_3 NPs, respectively. The concentric rings in selected-area electron diffraction (SAED) patterns (Fig. 1c, f) are assigned to the (220), (311), (400), (422), and (440) lattice planes, and (214), (116), (024), (113), (104) and (012) lattice planes, matching well with the inverse spinel phase of Fe_3O_4 [45,46], and hematite phase of Fe_2O_3 NPs [47,48], respectively. The crystal structures of ultrasmall Fe_3O_4 and Fe_2O_3 NPs are further verified by X-ray diffraction (XRD) spectroscopy (Fig. S2) (JCPDS No.

82-1533; No. 33-0664).

2.2 SERS activity of ultrasmall Fe_3O_4 NPs.

The prepared ultrasmall Fe_3O_4 NPs with good dispersity are believed to be a potential substrate for studying metal oxide material SERS activity, leveraging the multiple valence states of Fe. SERS measurements of Fe_3O_4 NPs are performed with crystal violet (CV), 4-Mercaptobenzoic acid (4MBA), Rhodamine 6G (R6G), and 4-aminothiophenol (4ATP) probe molecules (Fig. 2a-b, and Fig. S3). Based on the above SERS spectra results, Fe_3O_4 substrate exhibits remarkable SERS activity; the molecular limit of detection (LOD) of Fe_3O_4 NPs SERS substrate can reach 5×10^{-9} M. Even as the CV concentration is diluted to 10^{-9} M, the Raman vibration peaks (1176 cm^{-1} vibration mode: C-C stretching, 1615 cm^{-1} vibration mode: ring stretching) [49] of CV molecule are still observable. For non-resonance molecule, as the concentrations of 4MBA and 4ATP probe molecules are reduced to 6×10^{-7} M, ring stretching vibration modes (4MBA: 1590 cm^{-1}, 4ATP: 1580 cm^{-1}) [34] are still intense. The EF of CV molecule on Fe_3O_4 NPs is calculated to be $\sim 9.06 \times 10^3$, and the selected CV molecular concentration for EF calculation is 5×10^{-5} M, avoiding false EF value caused by supersaturation adsorption effect (Fig. S4). EF for CV molecule adsorbed on Fe_3O_4 NPs are detailed calculated according to the following equation [38, 50], and the non-SERS signal intensity is acquired in solution directly.

$$EF = (I_{SERS} / N_{ads}) / (I_{Raman} / N_{Raman})$$

N_{ads} and N_{Raman} are the number of CV molecules adsorbed on Fe_3O_4 NPs, and non-SERS solution sample under the same laser illumination, respectively. I_{SERS} and I_{Raman} are the SERS peak (1615 cm^{-1}) intensity of CV molecules on Fe_3O_4 NPs, and the non-SERS solution sample signal of CV molecules, respectively. Laser spot size (532 nm) is ~ 1.3 μm, The laser spot size is determined by illumination laser (λ) and numerical aperture of the objective (50 ×; N.A: 0.5), Spot size = 1.22 λ / (N.A). For non-SERS solution sprctra measurment, CV ethanol solution is 0.02 M, focal plane of 532 nm laser is 1.32 μm^2, the depth of field penetration (h) =n λ / $(N.A)^2$, n is the refractive index of the surrounding media, h = 2.89 μm. N_{Raman} = 0.02 mol/L × 1.32μm^2 × 2.89 μm × 6.02 × 10^{23} mol^{-1}. N_{Raman} is estimated as ~ 4.60 × 10^7. The number of molecules adsorbed on Fe_3O_4 NPs is co-determined by laser spot size (532 nm: ~ 1.3 μm), and the density of CV molecules adsorbed on the Fe_3O_4 NPs (~ 0.5 nM cm^{-2})[38, 50]. In order to avoid supersaturated adsorption, 5 × 10^{-5} M molecular concentration is selected (Fig. S4a). The effective adsorption coverage under laser illumination is evaluated based on the TEM image of Fe_3O_4 NPs (Fig. S1), SERS NPs in the total laser exposure area is estimated to be ~ 1/7, and the remaining 6/7 is blank region. Therefore, N_{ads} = 0.5 nM cm^{-2} × 6.02 × 10^{23} mol / L × π × $(0.65\ \mu m)^2$ × 1/7. The number of molecules adsorbed on the Fe_3O_4 NPs is concluded as ~ 5.71 × 10^5. I_{SERS} and I_{Raman} are the SERS peak,

and normal Raman peak intensities at 1615 cm^{-1} of CV molecules, the spectra comparison is based on 10 different laser spots acquirements, I_{SERS} = ~ 22500 and I_{Raman} = ~ 400 (Fig S4b). The integration time for SERS and Raman measurements are 1 s and 2 s, respectively. Taking above values into equation (1), EF is estimated to be ~ 9.06 × 10^3.

The SERS activity comparison between Fe_3O_4 and Fe_2O_3 NPs was evaluated (Fig. 2c). Fe_3O_4 NPs exhibit much higher SERS enhancement than their Fe_2O_3 counterparts. The feature of ring stretching vibration modes of CV (1615 cm^{-1}) and 4MBA molecules (1590 cm^{-1}) absorbed on Fe_3O_4 NPs are significantly enhanced. The surface potential of Fe_3O_4 and Fe_2O_3 NPs are measured by the zeta-potential experiment, and the results are ~ 15.950 ± 3.61 mV, and -0.175 ± 3.59 mV, respectively, which demonstrating that Fe_3O_4 NPs are positively surface charge, and Fe_2O_3 NPs are almost electroneutral. CV and R6G molecules are two positively charged molecules [33], the electrostatic adsorption effect between SERS substrate and molecule will induce more positively charged molecules (CV, R6G) adsorbed on Fe_2O_3 NPs. However, the observed SERS signal of the two positively charged molecules (CV, R6G) were more enhanced on Fe_3O_4 NPs (Fig. 2c and Fig. S5a), which illustrating surface potential differences are not the primary factor for SERS EF in Fe_3O_4-moecule, and Fe_2O_3 NPs-molecule surface complex. For Fe_3O_4@CV SERS system, an obvious Raman intrinsic peak shift of CV molecule is observed from 1622

to 1615 cm^{-1}, whereas this Raman peak shift in Fe_2O_3@CV SERS system is indistinguishable. Similarly, legible 4MBA Raman peak shift is observed in Fe_3O_4@4MBA SERS system. All of these results indicate that stronger interaction occurs in Fe_3O_4 substrate-molecule SERS system than in Fe_2O_3 NPs.

2.3 PICT process promoted by multiple electronic energy levels.

The mentioned Raman peak shift is an intriguing symbol of PICT process for chemical enhancement mechanism [37]. Selective enhancement feature of non-totally symmetric vibration modes (b_2 modes) is also observed in the control SERS measurements of Fe_3O_4 and Fe_2O_3 NPs (Fig. 2c). C-H vibration peak (1065 cm^{-1}) is assigned as a b_2 mode of 4MBA molecule [38], which is more enhanced than other Raman peaks in Fe_3O_4 SERS system. Besides, similar enhancement of b_2 modes at 1140, 1385, and 1445 cm^{-1} [49] is noticed in Fe_3O_4@4ATP SERS system (Fig. S5b), possessing noticeable and selective SERS enhancement compared to other Raman vibration modes. Intense SERS enhancement of Fe_3O_4 NPs ascribed to PICT process mechanism is clearly verified via the selectively enhanced b_2 modes, which matches well with the Herzberg-Teller selection rule [51]. In order to further investigate the interfacial charge transfer process between the NPs and probe molecules, the charge density redistributions of Fe_3O_4@4MBA and Fe_2O_3@4MBA SERS systems are quantitatively calculated via density functional theory (DFT). As shown in

Fig. 2d, the 4MBA molecules are bonded to the surfaces of Fe_3O_4 and Fe_2O_3 via S-Fe bonding [20,37], which serves as the interfacial charge transfer channel, and facilitates the redistribution of the electron cloud around the 4MBA molecule and SERS substrate. The results show that the charge density deformation mainly occurs around the S atom of 4MBA, where the charge accumulation region is concentrated on S atoms (yellow region), and the charge depletion region is mainly around Fe atoms (blue region) in the SERS platforms. The bader charge analysis indicates that the electron-transfer direction is from SERS substrate to probe molecule, and the amount of charge transfer from Fe_3O_4 to 4MBA molecule is 0.396 *e*, which is definitely larger than that of the Fe_2O_3 counterpart (0.202 *e*). The results directly confirm the interfacial PICT enhancement mechanism and demonstrate Fe_3O_4 substrate enabling more electrons to be transferred to probe molecule, suggesting higher efficiency PICT process and stronger SERS activity.

In order to investigate in-depth SERS effect of Fe_3O_4 NPs originating from specific surface physicochemical electronic structure, high efficiency PICT process of ultrasmall Fe_3O_4 NPs is systematically studied via UV-vis diffuse reflectance/absorption spectroscopy, X-ray photoelectron spectroscopy (XPS), and first-principles DFT simulations (Fig. 3). Ferric and divalent Fe-induced multiple electronic energy levels exist between O *2p* valence band (VB) and empty Fe *4s* conduction band (CB) [52-54].

Crystal field bands of Fe_3O_4 NPs are mainly made up of multiple electronic energy levels derived from multiple valence states of Fe occupying octahedral (Fe^{3+}, Fe^{2+}) and tetrahedral sites (Fe^{3+}), which are composed of 3d metal atomic orbital in Fe_3O_4 NPs [52]. Energy level structure of Fe_3O_4 NPs is carefully explored by UV-vis diffuse reflectance and photoluminescence (PL) spectra measurements. The electronic transition processes from VB (O *2p*) to energy level $\mathbf{e_g}$ (~ 3.1 eV), VB (O *2p*) to energy level $\mathbf{t_2}$ (~ 1.8 eV), and energy level **e** to $\mathbf{t_2}$ (~ 0.9 eV) are clearly observed in UV-vis diffuse reflectance spectra shown in Fig. 3a. The obvious PL peak (~ 590 nm, ~ 2.1 eV) of Fe_3O_4 NPs is attributed to radiative recombination of excitons transferred from energy levels $\mathbf{e_g}$ to $\mathbf{t_{2g}}$ (Fig. S6). These results confirm the existence of $\mathbf{e_g}$, $\mathbf{t_{2g}}$, **e,** and $\mathbf{t_2}$ crystal field bands in forbidden energy gap of Fe_3O_4 NPs, which provide more electron transfer routes and endow high-efficiency interfacial PICT process from SERS substrate to probe molecule due to multiple electronic energy levels.

The efficient PICT process promoted by multiple electronic energy levels is further verified by XPS measurement and UV-vis absorption spectroscopy. Binding energy of S *2p* electrons in 4MBA@Fe_3O_4 surface complex is more negatively shifted compared to that of 4MBA@Fe_2O_3, demonstrating a larger amount of electron transfer-induced nuclear potential offset occurs in 4MBA@Fe_3O_4 SERS system (Fig. 3b) [30,55].

Besides, XPS results suggest an efficient charge transfer process between Fe_3O_4 NPs and 4MBA molecule, which greatly increases molecular polarizability and molecular Raman scattering cross section. Moreover, 4MBA adsorbed on Fe_3O_4 NPs exhibits an intense absorption peak in the visible region (~ 530 nm) compared to Fe_2O_3 shown in Fig. 3c, which is ascribed to high-efficiency PICT resonance with light illumination. Wavelength-dependent SERS measurement of 4MBA on Fe_3O_4 NPs was also carried out (Fig. S7). An extremely high SERS spectra signal is acquired under 532 nm laser illumination, which is consistent with the UV-vis absorption spectroscopy result. In brief, multiple valence states of Fe-induced electronic energy levels in Fe_3O_4 materials such as crystal field bands of octahedral (Fe^{3+}, Fe^{2+}) and tetrahedral sites (Fe^{3+}), could significantly promote interfacial electron escape from Fe_3O_4 substrate, and its electron subsequent transfer to probe molecule, which enable a more facile and high-efficiency PICT process. It is noticed that there is almost no surface plasmon resonance (SPR) absorption peak around 532 nm (Fig. 3c), hence, the electromagnetic enhancement mechanism is ruled out here. To deeply delve into the remarkable SERS activity of Fe_3O_4, the electronic DOS of ultrasmall Fe_3O_4 and Fe_2O_3 are further calculated by DFT simulations (Fig. 3d). The results demonstrate that Fe_3O_4 NPs exhibit obviously larger electronic DOS compared to the Fe_2O_3 counterpart, especially for the DOS near Fermi energy level. The projected DOS

indicates that the total DOS near Fermi energy level for Fe_3O_4 NPs is contributed by the Fe ions existing in the octahedral and tetrahedral sites together (Fig. S8), which is consistent with UV-vis diffuse reflectance spectroscopy result. The abundant electronic DOS in the vicinity of the Fermi energy level for ultrasmall Fe_3O_4 NPs affords sufficient electronic states for photonic resonance, greatly boosting the matter-light interaction, and facilitating more electrons participating in Raman enhancement activity. Moreover, the DFT results indicate that the band gap of Fe_3O_4 NPs is narrower than that of Fe_2O_3 NPs due to the upshifted VB band caused by the multiple crystal field energy levels (Fig. 3d). The narrow band gap of ultrasmall Fe_3O_4 NPs can significantly promote the vibronic coupling in PICT and exciton resonance system, increasing the charge transfer possibilities via borrowing intensity from neighboring exciton resonance, and further contributing to the ultrasensitive SERS activity.

2.4 SERS bioprobe utilized in CTCs detection.

The features of ultrahigh SERS activity, excellent biocompatibility, good anti-interference ability, and selective SERS enhancement are expected to make ultrasmall Fe_3O_4 NPs exhibit special advantages in cancer detection and oncological imaging, especially for circulating tumor cell (CTC) detection and cancer cell imaging based on the nanoscale spatial resolution. CTC serves as an important indicator in early cancer screening, and plays a significant role in postoperative evaluation [56]. However, there still

exists huge challenge to rapidly and accurately detect the rare CTCs existed in peripheral blood. Fortunately, ultrasmall Fe_3O_4 NPs SERS platform adsorbed with Raman signal molecule presents high-sensitive and specific fingerprint spectrum, satisfying extremely rare CTC detection requirement. In order to improve the specificity of Fe_3O_4 SERS spectra for CTC detection, optimizing water solubility and targeting ability of Fe_3O_4 NPs-based SERS bioprobe are two key factors for high-efficiency cancer cell detection. Alizarin red (AR) Raman signal molecule, polydopamine (PDA), and polypeptide GE11 (amino acid sequence YHWYGYTPQNVI) are successively modified to the surface of Fe_3O_4 NPs, thus Fe_3O_4-AR-PDA-GE11 SERS bioprobe is successfully designed as shown in Fig. 4a. The reason for choosing Alizarin red (AR) as Raman signal molecule is due to the fact that AR molecule adsorbed on Fe_3O_4 NPs through an efficient and strong chemical bond [57], which is benefit for establishing SERS bioprobe with high stability, and the LOD of AR molecule on Fe_3O_4 SERS substrate can reach 6×10^{-8} M (Fig. S9). PDA layer is coated on Fe_3O_4-AR surface complex (Fig. S10), which is in favor of boosting water solubility, and improving cell enrichment capacity. Polypeptide GE11 is conjugated to Fe_3O_4-AR-PDA SERS bioprobe via amide bond linkage between carboxyl (GE11) and amino (PDA) [58] groups, which is clearly verified by Fourier transform infrared spectroscopy (Fig. S11). Fe_3O_4-AR-PDA-GE11 SERS bioprobe can effectively target tumor cells with evident

epidermal growth factor receptor (EGFR) expression due to the high binding efficiency between GE11 and EGFR [59], demonstrating it as a SERS bioprobe with high detection specificity for cancerous cells.

To investigate the detection capability of Fe_3O_4-AR-PDA-GE11 for cancer cells, MCF7 and MDA-MB-231 triple negative breast (TNB) cancer cells were added to rabbit blood, which was used for simulating the CTCs environment of peripheral blood samples. Cancer cells with EGFR expression are able to be traced by Fe_3O_4-AR-PDA-GE11 SERS signal based on the strong Raman signal anti-interference ability in biological environment. SERS spectra measurements are carried out for recognizing MCF7 and MDA-MB-231 TNB cancer cells in peripheral blood sample, respectively. Significant Raman signature of AR molecule is obtained from SERS bioprobe targeted to the CTC. Raman vibration modes (1255 cm^{-1}: C=O stretching, 1325 cm^{-1}: CC group stretching, 1450/1465 cm^{-1}: combinations of CC, CH, and CO stretching) [60] are unambiguous even with single MCF7 and MDA-MB-231 TNB cancer cells in 2 mL rabbit blood, respectively (Fig. 4 b-c). SERS spectra of Fe_3O_4-AR-PDA-GE11 bioprobe exhibit high homogeneity and uniformity in CTC detection, while no Raman signal is detected in blood sample without cancer cells (Fig. S12). The Fe_3O_4-AR-PDA-GE11 SERS bioprobe exhibits outstanding detection specificity and sensitivity for cancer cells with EGFR expression, and semiconductor-based SERS bioprobe detecting of CTC, which can be

successfully deployed as an optimized and efficient bioprobe for early tumor diagnosis and postoperative monitoring.

2.5 High-resolution SERS imaging for cancer cells.

Encouraged by the excellent SERS sensitivity, high detection specificity, and good spectra uniformity of Fe_3O_4-AR-PDA-GE11 SERS bioprobe, SERS imaging abilities for distinguishing subtypes of breast cancer cells are explored. To evaluate the superiority of Fe_3O_4-based optical SERS mapping image, oncological cell imaging experiments were carried out on MCF7 and MDA-MB-231 TNB cancer cells. Although these two breast cancer cells in rabbit blood can be successfully detected via Raman signal, the subtypes of cancerous cells cannot be directly distinguished. Classifying the subtypes of tumor play a vital role in accurate diagnosis and guidance of cancer treatment, hence, opening up a direct method to quickly differentiate subtypes of breast cancer cells, which is of great clinical value. Fe_3O_4-AR-PDA-GE11 SERS bioprobes are incubated with the two cancer cells in processed rabbit blood sample. Two cancer cells can be quickly and clearly distinguished via high-resolution SERS mapping image, which is described in the schematic diagram (Fig. 5a). MDA-MB-231 TNB cancer cells have high EGFR expression, while that of MCF7 cancer cells is relatively low [61,62], which predominately affects these two cancer cellular uptake of Fe_3O_4-AR-PDA-GE11 SERS bioprobe. This cellular uptake feature can be quickly and intuitively identified by SERS

optical mapping image based on tracing the distribution of Fe_3O_4-AR-PDA-GE11 bioprobes. SERS imaging results demonstrate that significantly more EGFR expression-related uptake of Fe_3O_4-based bioprobes occurs in MDA-MB-231 TNB cancer cells than in MCF7 cells as shown in Fig. 5b, c. High-resolution SERS imaging is acquired by analyzing 1255 cm^{-1} (C=O vibration stretching) Raman modes of AR molecules, and exhibits a high degree of agreement with the optical image of cancer cells.

Larger amount of SERS bioprobes uptake by MDA-MB-231 TNB cancer cells inducing more intense SERS signal compared to MCF7 cancer cell can be seen in Fig. 4b-c. SERS optical images (Fig. 5b-c) of the two subtypes of breast tumor cells are highly consistent with the SERS signal intensity results. SERS mapping images clearly clarify that plentiful Fe_3O_4-AR-PDA-GE11 SERS bioprobes are distributed in different regions of MDA-MB-231 TNB cancer cells owing to the higher EGFR expression, while SERS bioprobes are mainly accumulated around the MCF7 cancer cell by means of nonspecific adsorption. The targeting ability of SERS bioprobes co-incubated with MCF7 and MDA-MB-231 TNB cancer cells are further confirmed by immunofluorescence staining images as shown in Fig. S13. Significantly more SERS bioprobes exist in MDA-MB-231 TNB cancer cells as observed by laser scanning confocal microscopy (LSCM), which is in good agreement with the SERS imaging results, and offers a

sufficient evidence to support the SERS imaging capability of the bioprobe in quickly differentiating above two breast cancer cells. Based on the inductively coupled plasma optical emission spectrometer (ICP-OES) measurements (Fig. S14), the quantity of Fe_3O_4-AR-PDA-GE11 SERS bioprobes taken up by MDA-MB-231 TNB cancer cells are ~ 2.3 times more than MCF7 cancer cells after 3 h coincubation, verifying that Fe_3O_4-based SERS bioprobes can accurately identify tumor subtypes with EGFR expression. Besides, high-resolution SERS image provides an opportunity to directly observe the quantity distribution and behavior of Fe_3O_4-based bioprobes at cellular level, and puts forward a novel approach to study the interactions between NPs and cells. SERS microimaging derived from Raman vibration modes presents unique superiority in distinguishing different subtypes of cancer cells *in vitro*, achieving the requirement of accurately diagnosing cancer cell via liquid biopsy strategy.

2.6 SERS-MRI dual-modal cancer imaging.

Developing a novel bioprobe with bimodal imaging capability is of great significance in the early screening and treatment of tumors. The Fe_3O_4-based bioprobes proposed in this study exhibit exciting SERS activity in oncological imaging at cellular level *in vitro*, and can also serve as a high-potential contrast agent for T_1-weighted magnetic resonance imaging (MRI), realizing active-targeted imaging of tumor tissues *in vivo*. Benefiting from abundant unpaired electrons of Fe^{3+}, and the decreased

spin-canted proportion derived from the reduced diameter of Fe_3O_4 NPs, ultrasmall Fe_3O_4-based bioprobes exhibit obvious MRI contrast agent activity. The superior T_1-weighted MRI contrast enhancement of the ultrasmall Fe_3O_4-based bioprobes can also be explained by the theory of inner-sphere (IS) and outer-sphere (OS) [6]. Concentration-dependent T_1-weighted MRI measurements were carried out *in vitro* with a 0.47 T MRI instrument (r_2/r_1 = 3.26) as shown in Fig. 6a. Fe_3O_4-based bioprobes exhibit the best contrast enhancement at 0.3 mM concentration. In order to investigate the capability of Fe_3O_4-AR-PDA-GE11 bioprobes for MRI *in vivo*, a subcutaneous MDA-MB-231 TNB tumor model in Balb/C nude mice was successfully established. Significant T_1-weighted MRI signal is observed as the tumor-bearing mice is injected with Fe_3O_4-based bioprobes (75 μL, 1 mg/mL) via tail vein injection. MRI of tumor *in vivo* was collected on a 3.0 T human clinical scanner after 50 min intravenous injection (Fig. 6b-c). Besides, damage of main organs is not obviously detected in the test mice after 14 days post-injection of Fe_3O_4-based bioprobes as shown in H&E staining images (Fig. S15), indicating that bioprobes possess appreciable histocompatibility and minimized toxicity to normal organs. Therefore, the rational design of SERS-MRI dual-modal imaging bioprobes will provide complementary cancer imaging information from cell to tissue level, which could provide great promise for image-guided tumor diagnosis *in vivo* and *in vitro*.

3. Conclusions

In summary, through designing a novel semiconductor nanomaterial, i.e., ultrasmall Fe_3O_4 NPs, remarkable SERS activity of 9.06×10^3 EF, and 5×10^{-9} M LOD for CV molecule is achieved. High-efficiency interfacial PICT process is promoted by multiple electronic energy levels derived from multiple valence states of Fe, which is observed by UV-vis diffuse reflectance spectroscopy. Multiple electronic energy levels offer sufficient electron transition routes for interfacial charge transfer in ultrasmall Fe_3O_4 SERS system. DFT calculations indicate that the feature of narrow band gap and high electronic DOS are in favor of establishing stable molecule@Fe_3O_4 SERS system with strong vibronic coupling resonance. The above-mentioned factors greatly increase molecular polarizability tensor and amplify molecular Raman cross section. CTC are quickly recognized via high-sensitivity SERS spectra, and subtypes of breast cancer cells are accurately distinguished through high-resolution SERS imaging, which is of great significance in tumor classification *in vitro*. Moreover, pure Fe_3O_4-based bioprobes are capable of serving as a T_1 MRI contrast agent for tumor imaging *in vivo*, achieving highly efficient tumor diagnostic ability based on the SERS-MRI dual-modal imaging modalities ranging from cell to tissue levels. This optimized SERS-MRI bimodal nanoprobe can be extensively utilized for early tumor diagnosis *in vivo* and *in vitro*, and has great application potential for image-guided tumor

treatment.

Figures

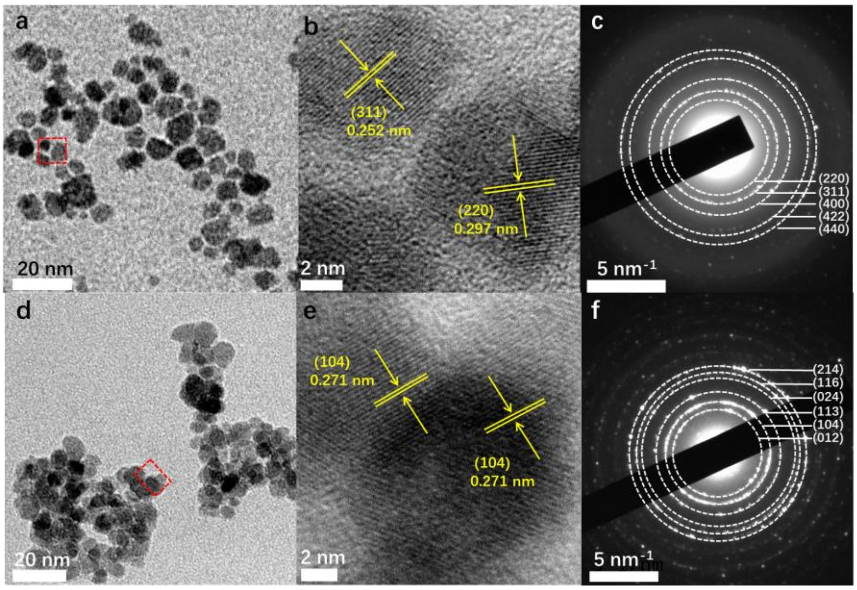

Fig. 1. TEM (a, d), HRTEM (b, e), and SEAD (c, f) images of ultrasmall Fe_3O_4 and Fe_2O_3 NPs, respectively.

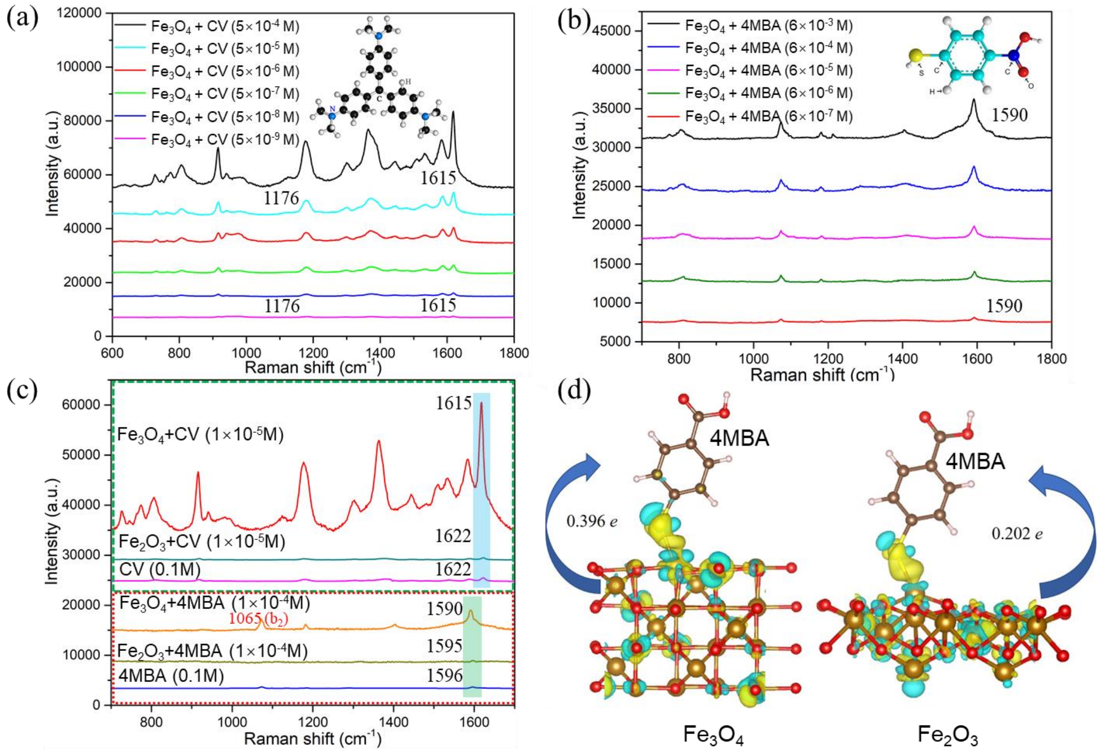

Fig. 2. SERS spectra of (a) CV and (b) 4MBA molecules absorbed on Fe_3O_4 NPs at different concentrations, respectively. (c) Raman, SERS spectra comparison of CV and 4MBA molecules adsorbed on Fe_3O_4 and Fe_2O_3 NPs, respectively. Laser wavelength: 532 nm; laser power: 0.5 mW; lens: 50 × objective; and acquisition time: 1 s. (d) Charge difference redistributions of 4MBA absorbed on Fe_3O_4 and Fe_2O_3 NPs. The yellow and blue colors stand for the electron accumulation and depletion regions, respectively, and the charge transfer direction and values are also indicated.

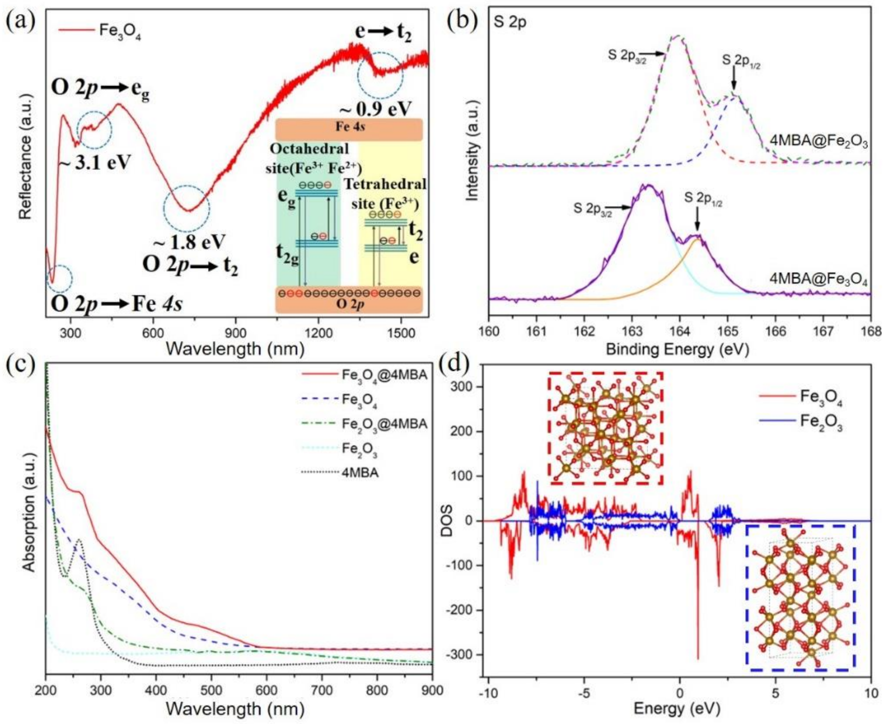

Fig. 3. (a) UV-vis diffuse reflectance spectrum of Fe_3O_4 NPs, and the energy level structure in forbidden band of Fe_3O_4 NPs. (b) XPS measurements of 4MBA@Fe_2O_3 and 4MBA@Fe_3O_4 SERS system. (c) UV-vis absorption spectra of Fe_3O_4@4MBA, Fe_2O_3@4MBA, Fe_3O_4, Fe_2O_3, and 4MBA; Fe_3O_4, Fe_2O_3: 100 μg/mL, 4MBA: 1×10^{-4} M. (d) Electronic DOS of Fe_3O_4 and Fe_2O_3 NPs on the basis of DFT calculation, and the corresponding slab model of Fe_3O_4 and Fe_2O_3 NPs.

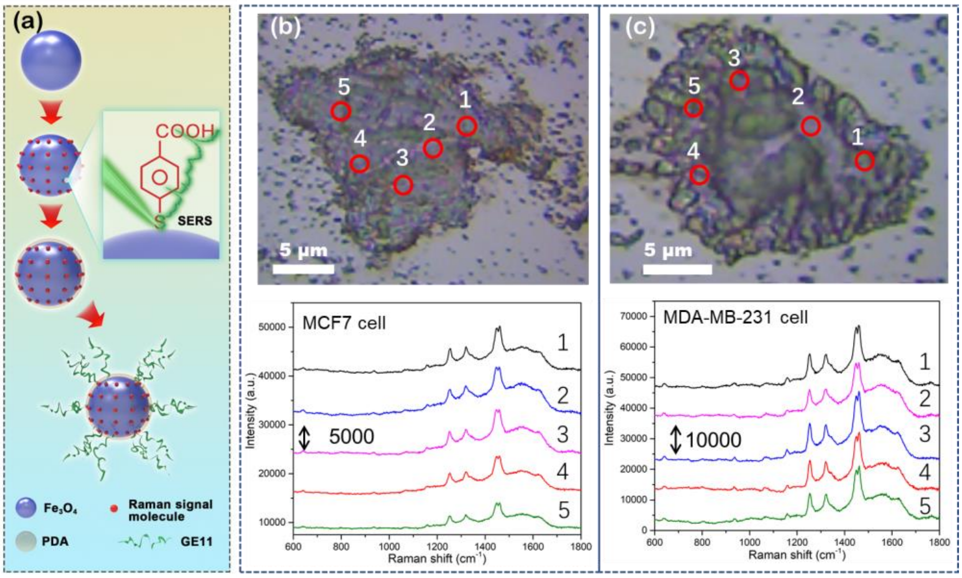

Fig. 4. (a) Schematic diagram of the synthetic preparation process for Fe_3O_4-AR-PDA-GE11 SERS bioprobe. SERS spectra of Fe_3O_4-AR-PDA-GE11 bioprobe collected from 5 different laser spots in rabbit blood samples with (b) MCF7 and (c) MDA-MB-231 TNB cancer cell, respectively. Laser wavelength: 532 nm; laser power: 0.2 mW; lens: 50 × objective; acquisition time: 1 s.

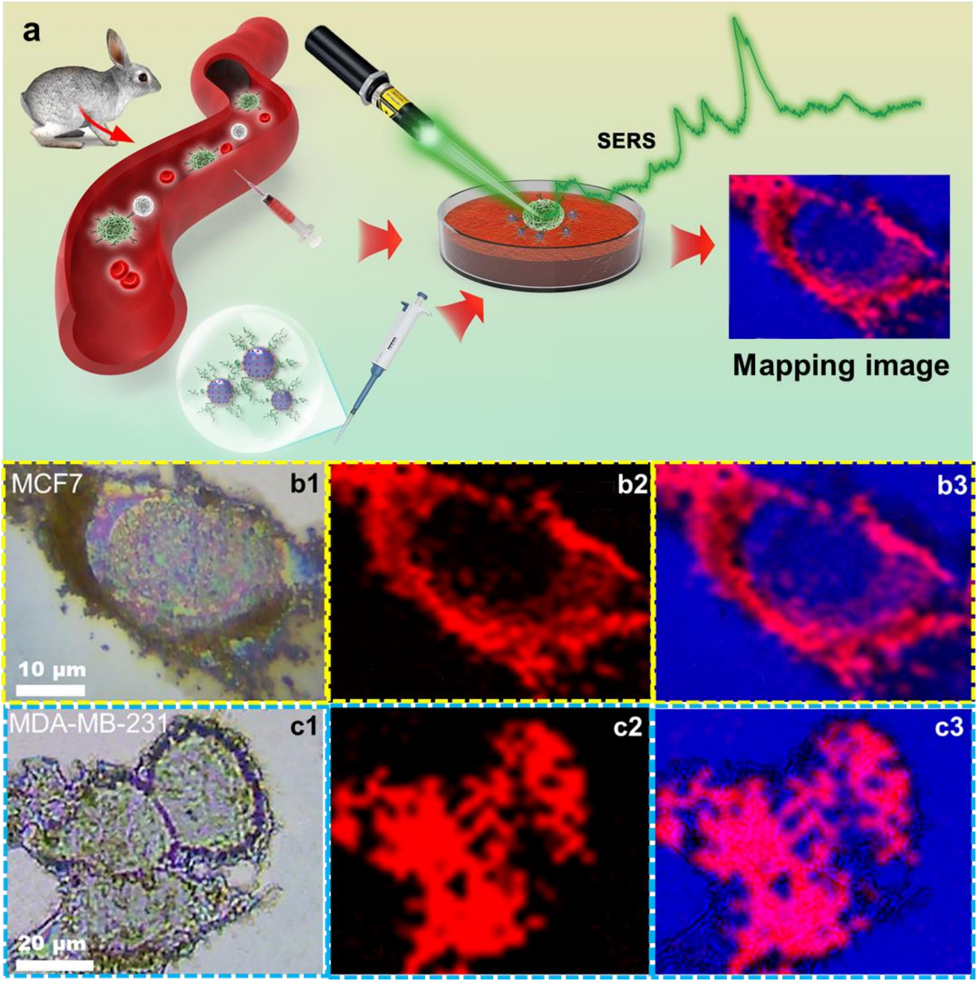

Fig. 5. (a) Schematic diagram of SERS mapping image differentiating cancer cells in rabbit blood sample. (b1, c1) Optical microscope images, (b2, c2) SERS images, and (b3, c3) overlapped optical and SERS images of Fe_3O_4-AR-PDA-GE11 SERS bioprobes distributed in MCF7 and MDA-MB-231 TNB cancer cells, respectively. Laser wavelength: 532 nm; laser power: 0.1 mW; and lens: 50 × objective.

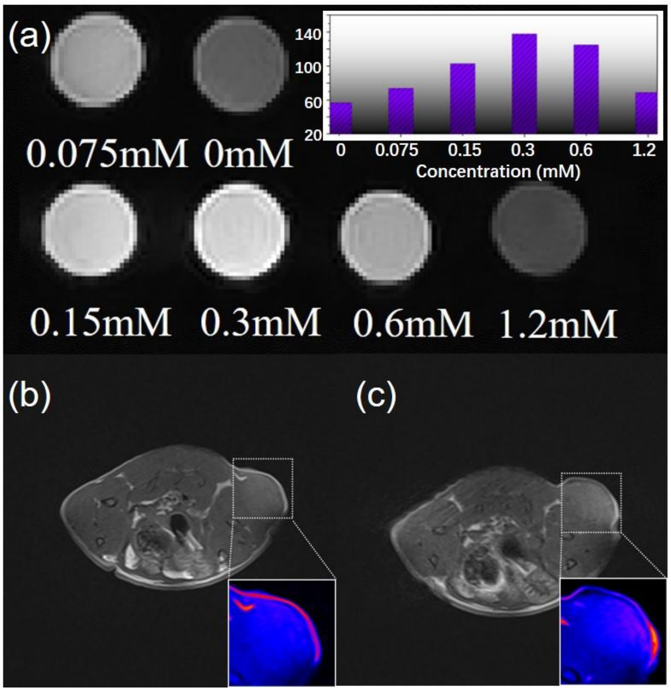

Fig. 6. (a) T_1-weighted MR images of Fe_3O_4-AR-PDA-GE11 bioprobes at different concentrations *in vitro*. (b, c) T_1-weighted MR images and the color-coded images of MDA-MB-231 TNB tumor-bearing nude mice before and 50 min after intravenous injection of Fe_3O_4-AR-PDA-GE11 bioprobes, respectively.

4. Materials and methods

4.1. Preparation of Fe_3O_4 nanoparticles

Ultrasmall Fe_3O_4 nanoparticles were synthesized via coprecipitation method. Briefly, 12 mmol of citric acid was dissolved in 80 mL deionized water, then the solution was heated up to 65 °C. Meanwhile, 80 mL of iron precursor solution (8 mmol of $FeCl_3$ and 5 mmol of $FeCl_2$) were quickly injected under magnetically stirred in a nitrogen atmosphere, followed by drip addition of 240 mL ammonia solution (5%). After 24 h, the ultrasmall Fe_3O_4 nanoparticles were synthesized. Furthermore, the products could be purified by dialyzing in deionized water for 6 times, by which the unreacted and residual ions were removed from the colloids.

4.2. Preparation of Fe_2O_3 nanoparticles

Ultrasmall Fe_2O_3 NPs were prepared by high temperature calcination of above Fe_3O_4 NPs at 650 °C in a tube furnace for 3 h. Furthermore, the product was rinsed with de-ionized water and ethanol several times, and dried in a vacuum oven at 80 °C for 10 h.

4.3. Preparation of Fe_3O_4-AR-PDA-GE11 SERS bioprobe

50 mL purified Fe_3O_4 NPs were mixed with 50 mL alizarin red (AR) solution (1×10^{-4} M) under mechanical stirring. After 16 h, Fe_3O_4-AR NPs was separated by centrifugation (10000 rpm, 10 min), and washed twice to

remove excess AR. Then, a simplified method was used to coat the surface of Fe_3O_4-AR NPs with polydopamine (PDA). In a facile procedure, 50 mL Fe_3O_4-AR (1 mg/mL in tris-HCl buffer solution, pH 8.5) was transferred into a 250 mL beaker, and 50 mL PDA (1 mg/mL in trimethylolamine-HCl buffer solution, pH 8.5) was added dropwise into it. The mixture was stirred for 4 h, separated by centrifugation and washed thrice with deionized water. The purified Fe_3O_4-AR-PDA bioprobes were re-immersed in PBS buffer solution for storage and future use. GE11 was conjugated to the surface of Fe_3O_4-AR-PDA through the reaction between GE11 (-COOH) and PDA (-NH_2). Typically, GE11 (1.0 mg/mL in PBS, pH 7.4) and Fe_3O_4-AR-PDA (1.0 mg/mL in PBS, pH 7.4) solutions were mixed at equal volumes and stirred overnight at room temperature. After the reaction, the prepared Fe_3O_4-AR-PDA-GE11 dispersion was centrifuged and dispersed in ultrapure water.

4.4. Materials characterization

Transmission electron microscopy (TEM) and high-resolution TEM (HRTEM) images were obtained by Talos F200x. UV-vis diffuse reflectance/absorption spectra were collected on a UV-3600 UV-Vis-NIR spectrophotometer made by Shimadzu, Japan. X-ray photoelectron spectroscopy (XPS) was acquired by a Kratos Axis Ultra DLD instrument equipped with an Al anode (Al-Kα = 1486.7 eV). Inductively coupled

plasma optical emission spectrometry (ICP-OES) was measured an Optima 2100 instrument from Perkin Elmer. Photoluminescence spectra were collected by a He-Cd laser (325 nm) as excitation illumination. Infrared spectroscopy was acquired by intelligent Fourier infrared spectrometer (FTIR) (NICOLET 6700). Raman spectra and SERS images were obtained by Renishaw inVia Reflex instrument, England. X-ray diffraction (XRD) of the powder samples were characterized by the Rigaku Rotaflex Dmax2200 diffractometer (Japan) with Cu Kα radiation (λ = 1.54056 Å). MRI in vivo is obtained by 1.5 T human clinical scanner (Ingenia, Philips, Netherlands). MRI in vitro is acquired by MesoMR23 0.47 T scanner (Shanghai Niumag Corporation). Laser scanning confocal microscope (LSCM) images were obtained by Leica DMi8 (Germany).

4.5. SERS mapping

High-resolution SERS mapping imaging was acquired based on spot-to-spot Raman spectra collection on a 50 × 50 μm^2 area. In this area, MCF-7 and MDA-MB-231 TNB tumor cells targeted by Fe_3O_4-AR-PDA-GE11 bioprobes were on Si platform. Plenty of SERS spectra was acquired by the acquisition platform with 1 μm scan step upon 532 nm laser illumination, and with acquisition time of 0.5 s. SERS mapping image was obtained by analyzing the Raman vibration peak (1255 cm^{-1}) of alizarin red (AR) molecule.

4.6. SERS spectrum

SERS experiment was conducted in water. Typically, Fe_3O_4 NPs water suspension was mixed with probe molecules to obtain a final solution concentration, and the Fe_3O_4 NPs-probe molecules were kept for 5 h. Then, highly diluted Fe_3O_4 NPs-probe molecule solutions with different concentrations were dropped onto a clean Si platform and thoroughly rinsed with water several times to remove the unabsorbed probe molecules. Fe_3O_4 NPs-probe molecules were then subjected to SERS analysis. SERS signal were collected 90 min later as the water was completely volatile.

4.7. Processing of rabbit blood sample

Animal experiments were conducted with the approved protocol of institutional animal care and use committee (IACUC). Rabbit blood samples were obtained from the heart of healthy rabbit. Cancer cells were added into rabbit blood samples to simulate CTCs environment. 1 mL PBS was added to 2 mL rabbit blood samples to dilute the blood sample, followed by the addition of 2 mL lymphocyte separation solution. The blood sample was centrifuged at 1500 rpm for 20 min, and the obtained white layer was transferred to a centrifuge tube with 4 mL PBS (10 mmol). Then, the acquired sample was centrifuged at 1000 rpm for 5 min, removing the supernatant using a pipette and leaving bottom cells (~ 0.5

mL). 4 mL PBS solution (10 mmol) was added to the sample with 1000 rpm centrifugation (5 min), followed by removing 3 mL supernatant. Then, the SERS bioprobe (200 μL) was dropped into the sediment solution, which was incubated at 37 °C for 30 min. Subsequently, precipitation was collected by centrifugation at 1000 rpm for 5 min. Finally, the precipitate was evenly dispersed in the PBS solution (200 μl), and signal of SERS bioprobe targeted to cancer cell was acquired.

4.8. Cell culture

Human breast cancer cell lines MCF-7 and MDB-MA-231 were cultured in the DMEM medium supplemented with 10 wt% fetal bovine serum (FBS), 100 units mL^{-1} of penicillin, and 100 mg mL^{-1} of streptomycin. The cells were incubated at 37° C in a humidified atmosphere containing 5% of CO_2.

4.9. MRI in vitro and in vivo

MR imaging and relaxivity of the Fe_3O_4-based bioprobes were tested by MR analyzing system (MesoMR23-060H-I, Niumag, Shanghai) with the magnetic field of 0.47 T. Briefly, different concentrations (0.075, 0.15, 0.3, 0.6, 1.2 mM) of Fe_3O_4-based bioprobes in deionized water were used to measure their longitudinal relaxivity (r_1) and transverse relaxivity (r_2). T_1-weighted MRI was performed with spin echo sequence (TR = 600 ms, TE

= 18.2 ms). For MRI *in vivo*, MDB-MA-231 tumor bearing Balb/C nude mice (4-6 weeks) were purchased from Nanjing Cavins Biotechnology Co., Ltd (Nanjing, China). The mice were anesthetized by intraperitoneal injection of chloral hydrate solution (8 wt %), and then injected with 75 μL nanoprobe (1 mg/mL) through tail veins. The T_1-weighted images were acquired using a 3.0 T human clinical scanner (Siemens, Germany) of HwaMei Hospital, University of Chinese Academy of Sciences, China.

4.10. Immunofluorescence staining experiment

For LSCM, 2.0 mL of MCF-7 or MDB-MA-231 cells in growth medium were seeded into each glass bottom dish with the size of ø15 at a density of 5×10^4 cells/mL and allowed to adhere at 37 °C for 24 h. The growth medium was then replaced with a fresh one containing 0.15 mg/mL of Fe_3O_4-based bioprobes. After 4 h incubation, the cells were washed thrice with PBS. The cells were then fixed with 4 % formaldehyde for 30 min, permeabilized with 0.1 % triton for 5 min, blocked with 1.0 % BSA for 30 min and treated with the mixture of Hoechst (5 μg/mL) and FITC phalloidine (5 U/mL) for 30 min at room temperature. The samples were simultaneously excited at 405, 488, and 543 nm and the fluorescent images at emission wavelengths of 420-480, 500-540, and 600-660 nm were observed by a LSCM (Leica, Germany), respectively.

4.11. ICP-OES experiment

2.0 mL of MCF-7 or MDB-MA-231 cells in growth medium were seeded into each well at a density of 400000 cells/mL and allowed to adhere at 37 °C for 24 h. The growth medium was then replaced with a fresh one containing Fe_3O_4-based bioprobe (150μg/mL). After further 3 h incubation, the cells were washed thrice with PBS to remove unabsorbed bioprobe. Afterward, the cells were detached with trypsin and collected into a tube. The collected cells were digested with aqua regia and the content of iron ion was determined by ICP-OES.

4.12. In vivo toxicity experiments

For in vivo toxicity, female Balb/C nude mice (4-6 weeks) purchased from Nanjing Cavins Biotechnology Co., Ltd (Nanjing, China) were divided into four groups (3 each group), and were intravenously injected with different concentration of bioprobes (1, 12.5, 25mg/kg) and PBS as control. After 14 days, the mice were sacrificed and their major organs were subjected to the Haematoxylin and Eosin (H&E) staining and histopathological assessment.

4.13. Simulation methods

The spin-polarized density functional theory (DFT) computations were carried out using the Vienna ab initio simulation package (VASP v.5.4.1).

During all calculations, the generalized gradient approximation (GGA) and the projector augments wave (PAW) pseudopotentials with the exchange and correlation in the Perdew-Burke-Ernzerhof (PBE) were employed to describe the ion-electro interaction. A kinetic-energy cut-off of 550 eV was used for the plane wave basis set. The convergence threshold was set as 10^{-5} eV in energy and 0.02 eV/Å in force, respectively. The DFT+U technique was applied to the Fe atoms to depict the strong on-site coulomb repulsion between the Fe d-shell electrons, where the *U-J* parameters was set to 4.3 eV. In this work, the bulk α-Fe_2O_3 crystallizes in the hexagonal structure with antiferromagnetic order and the bulk Fe_3O_4 crystallizes in the inverted cubic spinel structure with the magnetic moments of the Fe ions exist on tetrahedral sites antiparallel to the Fe ions exist on octahedral sites were constructed, respectively. For bulk α-Fe_2O_3, the Monkhorst-Pack Gamma-centered grids with a 5 × 5 × 2 mesh for relaxations and a 15 × 15 × 6 mesh for the calculation of DOS were used. For bulk Fe_3O_4, the Monkhorst-Pack Gamma-centered grids with a 3 × 3 × 3 mesh for relaxations and a 9 × 9 × 9 mesh for the calculation of DOS were used. To model the interaction between Fe_3O_4 (α-Fe_2O_3) and adsorbed molecule, the (001) surface of Fe_3O_4 (α-Fe_2O_3) was constructed using the 1 × 1 (2 × 2) slab models with 20 Å thick vacuum layer added along the z direction. For all slab models, the Monkhorst-Pack Gamma-centered grids with a 3 × 3 × 1 mesh for structure optimizations and a 6 × 6 × 1 mesh for the calculation of charge

distributions were used. All structures were visualized using the program VESTA.

Declaration of Competing Interest

The authors declare that they have no known competing financial interests or personal relationships that could have appeared to influence the work reported in this paper.

Acknowledgements

This work was supported by the funding from National Natural Science Foundation of China (52002380, 32025021, 31971292, 51902012, 51873225), National Key R&D Program of China (2019YFA0405603), Zhejiang Province Key Research Project (2020C03110, 2019C03058), Strategic Priority Research Program of Chinese Academy of Sciences (XDB36000000), Zhejiang Provincial Natural Science Foundation of China (LQ20E020003, LQ19H180002), Key Scientific and Technological Special Project of Ningbo City (2020Z094, 2017C110022), Ningbo 3315 Innovative Teams Program (2019A-14-C), Key Laboratory of Diagnosis and Treatment of Digestive System Tumors of Zhejiang Province (2019E10020).

Appendix A. Supporting information

Supplementary material associated with this article can be found, in the online version, at doi:10.1016/j.fmre.2021.XX.XXX.

References

[1] H. Sung, J. Ferlay, R.L. Siegel, M. Laversanne, I. Soerjomataram, A. Jemal, F. Bray, Global cancer statistics 2020: GLOBOCAN estimates of incidence and mortality worldwide for 36 cancers in 185 countries, CA Cancer J. Clin. 71 (2021) 1-41.

[2] Z. Shen, A. Wu, X. Chen, Current detection technologies for circulating tumor cells, Chem. Soc. Rev. 46 (2017) 2038-2056.

[3] R.L. Siegel, K.D. Miller, A. Jemal, Cancer statistics, CA Cancer J. Clin. 68 (2018) 7-30.

[4] J. Kim, Y. Piao, T. Hyeon, Multifunctional nanostructured materials for multimodal imaging, and simultaneous imaging and therapy, Chem. Soc. Rev. 38 (2009) 372.

[5] Z.J. Zhou, R.L. Bai, J. Munasinghe, Z.Y. Shen, L.M. Nie, X.Y. Chen, T_1-T_2 Dual-Modal Magnetic Resonance Imaging: From Molecular Basis to Contrast Agents, ACS Nano 11 (2017) 5227-5232.

[6] J. Mike, M.V. Halbert, Z.R. Stephen, M.Q. Zhang, Iron Oxide Nanoparticles as T_1 Contrast Agents for Magnetic Resonance Imaging: Fundamentals, Challenges, Applications, and Prospectives, Adv. Mater. 33 (2021,): 1906539.

[7] S. Kunjachan, J. Ehling, G. Storm, F. Kiessling, T. Lammers, Noninvasive Imaging of Nanomedicines and Nanotheranostics: Principles, Progress, and Prospects, Chem. Rev. 115 (2015) 10907-10937.

[8] R. Langer, R. Weissleder, Jama, Nanotechnology 313 (2015) 135-136.

[9] W. He, O.T. Bruns, M.G. Kaul, E.C. Hansen, M. Barch, A. Wiśniowska, O. Chen, Y. Chen, N. Li, S. Okada, J.M. Cordero, M. Heine, C.T. Farrar, D.M. Montana, G. Adam, H. Ittrich, A. Jasanoff, P. Nielsen, M.G. Bawendi, Exceedingly small iron oxide nanoparticles as positive MRI contrast agents, Proc Natl Acad Sci USA 114 (2017) 2325-2330.

[10] J. Lin, W. Ren, A. Li, C. Yao, T. Chen, X. Ma, X. Wang, A. Wu, ACS Appl. Mater. Inter., Crystal-Amorphous Core-Shell Structure Synergistically Enabling TiO_2 Nanoparticles' Remarkable SERS Sensitivity for Cancer Cell Imaging 12 (2020) 4204-4211.

[11] Q.L. Wei, H. Arami, H.A. Santos, H.B. Zhang, Y.Y. Li, J. He, D.N. Zhong, D.S. Ling, M. Zhou, Intraoperative Assessment and Photothermal Ablation of the Tumor Margins Using Gold Nanoparticles, Adv. Sci. 8 (2021) 2002788.

[12] S. Pal, A. Ray, C. Andreou, Y. Zhou, T. Rakshit, M. Wlodarczyk, M. Maeda, R. Toledo-C., N. Berisha, J. Yang, H.-T. Hsu, A. Oseledchyk, J. Mondal, S. Zou, M.F. Kircher, DNA-enabled rational design of fluorescence-Raman bimodal nanoprobes for cancer imaging and therapy, Nat. Commun. 10 (2019) 1926.

[13] J. Wang, K. M. Koo, Y. L. Wang, M. Trau, Engineering State-of-the-Art Plasmonic Nanomaterials for SERS-Based Clinical Liquid Biopsy Applications, Adv. Sci. 6 (2019) 1900730.

[14] H. Rupa, V. Krishnan, B. Tan, Non plasmonic semiconductor quantum SERS probe as a pathway for in vitro cancer detection, Nat. Commun. 9 (2018) 3065.

[15] S. Ganesh, K. Venkatakrishnan, B. Tan, Quantum scale organic semiconductors for SERS detection of DNA methylation and gene expression, Nat. Commun. 11 (2020) 1135.
[16] E.D. Feng, T.T. Zheng, X.X. He, J.J. Chen, Y. Tian, A novel ternary heterostructure with dramatic SERS activity for evaluation of PD-L1 expression at the single-cell level. Sci. Adv. 4 (2018) eaau3494.
[17] S. Nie, S.R. Emory, Probing Single Molecules and Single Nanoparticles by Surface-Enhanced Raman Scattering, Science 275 (1997) 1102-1106.
[18] J.F. Li, Y.F. Huang, Y. Ding, Z.L. Yang, S.B. Li, X.S. Zhou, F.R. Fan, W. Zhang, Z.Y. Zhou, D.Y. Wu, B. Ren, Z.L. Wang, Z.Q. Tian, Shell-isolated nanoparticle-enhanced Raman spectroscopy, Nature 464 (2010) 392-395.
[19] M. Yilmaz, E. Babur, M. Ozdemir, R.L. Gieseking, Y. Dede, U. Tamer, G.C. Schatz, A. Facchetti, H. Usta, G. Demirel, Nanostructured organic semiconductor films for molecular detection with surface-enhanced Raman spectroscopy, Nat. Mater. 16 (2017) 918-924.
[20] I. Alessandri, J.R. Lombardi, Enhanced Raman Scattering with Dielectrics, Chem. Rev. 116 (2016) 14921-14981.
[21] S.M. Azarin, Y. Ji, R.M. Gower, B.A. Aguado, M.E. Sullivan, A.G. Goodman, E.J. Jiang, S.S. Rao, Y. Ren, S.L. Tucker, In vivo capture and label-free detection of early metastatic cells, Nat. Commun. 6 (2015) 8094-8094.
[22] M.P. Pancorbo, K. Thummavichai, L. Clark, T.A. Tabish, J. Mansfield, B. Gardner, H. Chang, N. Stone, Y.Q. Zhu, Novel Au-SiO_2-WO_3 Core-Shell Composite Nanoparticles for Surface-Enhanced Raman Spectroscopy with Potential Application in Cancer Cell Imaging, Adv. Funct. Mater. 29 (2019) 1903549.
[23] W.K. Wang, F. Zhao, M.Z. Li, C.P. Zhang, Y.H. Shao, Y. Tian, A SERS Optophysiological Probe for the Real-Time Mapping and Simultaneous Determination of the Carbonate Concentration and PH Value in a Live Mouse Brain, Angew. Chem. Int. Edit. 58 (2019) 5256-5260.
[24] A. Kapara, V. Brunton, D. Graham, K. Faulds, Investigation of cellular uptake mechanism of functionalised gold nanoparticles into breast cancer using SERS, Chem. Sci. 11 (2020) 5819-5829.
[25] S. Laing, L.E. Jamieson, K. Faulds, D. Graham, Surface-enhanced Raman spectroscopy for in vivo biosensing, Nat. Rev. Chem. 1 (2017) 0060.
[26] Y. Fang, N.H. Seong, D.D. Dlott, Measurement of the distribution of site enhancements in surface-enhanced Raman scattering, Science 321 (2008) 388-392.
[27] X.Y. Chu, X. Hong, P. Zou, J. Men, Y.C. Liu, Ultrasensitive protein detection in terms of multiphonon resonance Raman scattering in ZnS nanocrystals, Appl. Phys. Lett. 98 (2011) 065102.
[28] X.T. Wang, L. Guo, SERS Activity of Semiconductors: Crystalline and Amorphous Nanomaterials, Angew. Chem. Int.Edit. 59 (2020) 4231-4239.
[29] A. Chakraborty, A. Ghosh, A. Barui, Advances in surface-enhanced Raman spectroscopy for cancer diagnosis and staging, J. Raman Spectrosc. 51 (2020) 7-36.
[30] A.R. Li, J. Yu, J. Lin, M. Chen, X. Wang, L. Guo, Increased O 2p State Density Enabling Significant Photoinduced Charge Transfer for Surface-Enhanced Raman Scattering of Amorphous $Zn(OH)_2$, J Phys Chem Lett. 11 (2020) 1859-1866.
[31] A. Li, J. Lin, Z. Huang, X. Wang, L. Guo, Surface-Enhanced Raman Spectroscopy on Amorphous Semiconducting Rhodium Sulfide Microbowl Substrates, iScience 10 (2018) 1-10.
[32] S. Cong, Y.Y. Yuan, Z.G. Chen, J.Y. Hou, M. Yang, Y.L. Su, Y.Y. Zhang, L. Li, Q.W. Li, F.X. Geng, Z.G. Zhao, Noble metal-comparable SERS enhancement from semiconducting metal oxides by making oxygen vacancies, Nat. Commun. 6 (2015) 7800.

[33] J. Lin, Y. Shang, X.X. Li, J. Yu, X.T. Wang, L. Guo, Ultrasensitive SERS Detection by Defect Engineering on Single Cu_2O Superstructure Particle, Adv. Mater. 29 (2017) 1604797.
[34] X.T. Wang, W.X. Shi, Z. Jin, W.F. Huang, J. Lin, G.S. Ma, S.Z. Li, L. Guo, Remarkable SERS Activity Observed from Amorphous ZnO Nanocages, Angew. Chem. Int. Edit. 129 (2017) 9983-9987.
[35] J.R. Lombardi, R.L. Birke, Theory of Surface-Enhanced Raman Scattering in Semiconductors, J Phys. Chem. C 118 (2014) 11120-11130.
[36] X.X. Xue, W.D. Ruan, L.B. Yang, W. Ji, Y.F. Xie, L. Chen, W. Song, B. Zhao, J.R. Lombardi, Surface-enhanced Raman scattering of molecules adsorbed on Co-doped ZnO nanoparticles, J. Raman Spectrosc. 43 (2012) 61-64.
[37] J. Lin, W. Hao, Y. Shang, X. Wang, D. Qiu, G. Ma, C. Chen, S. Li, L. Guo, Direct Experimental Observation of Facet-Dependent SERS of Cu_2O Polyhedra, Small 14 (2018) 1703274.
[38] X. Wang, W. Shi, S. Wang, H. Zhao, J. Lin, Z. Yang, M. Chen, L. Guo, Two-dimensional Amorphous TiO_2 Nanosheets Enabling High-efficiency Photo-induced Charge Transfer for Excellent SERS Activity, J. Am. Chem. Soc. 141 (2019) 5856-5862.
[39] J.J. Li, C. Wu, P.F. Hou, M. Zhang, K. Xu, One-pot preparation of hydrophilic manganese oxide nanoparticles as T1 nano-contrast agent for molecular magnetic resonance imaging of renal carcinoma in vitro and in vivo, Biosens. and Bioelectron. 102 (2018) 1-8.
[40] Z.Y. Shen, W.P. Fan, Z. Yang, Y.J. Liu, V.I. Bregadze, S.K. Mandal, B.C. Yung, L. Lin, T. Liu, W. Tang, L.L. Shan, Y. Liu, S.J. Zhu, S. Wang, W.J. Yang, L.H. Bryant, D.T. Nguyen, A.G. Wu, X.Y. Chen, Exceedingly Small Gadolinium Oxide Nanoparticles with Remarkable Relaxivities for Magnetic Resonance Imaging of Tumors, Small 15 (2019) 1903422.
[41] Y. Han, S. Lei, J. Lu, Y. He, Z. Chen, L. Ren, X. Zhou, Potential use of SERS-assisted theranostic strategy based on Fe_3O_4/Au cluster/shell nanocomposites for bio-detection, MRI, and magnetic hyperthermia, Mat. Sci. Eng. C 64 (2016) 199-207.
[42] X. Zhao, L. Zeng, N. Hosmane, Y. Gao, A. Wu, Cancer cell detection and imaging: MRI-SERS bimodal splat-shaped Fe_3O_4/Au nanocomposites, Chinese Chem. Lett. 30 (2019) 87-89.
[43] Z. Wang, J. Zhang, H. Wang, J. Hai, B. Wang, Se atom-induced synthesis of concave spherical Fe3O4@Cu2O nanocrystals for highly efficient MRI-SERS imaging-guided NIR photothermal therapy, Part. Part. Syst. Charact. 35 (2018) 1800197.
[44] Z.Y. Shen, T.X. Chen, X.H. Ma, W.Z. Ren, Z.J. Zhou, G.Z. Zhu, A. Zhang, Y.J. Liu, J.B. Song, Z.H. Li, H.M. Ruan, W.P. Fan, L.S. Lin, J. Munasinghe, X.Y. Chen, A.G. Wu, Multifunctional Theranostic Nanoparticles Based on Exceedingly Small Magnetic Iron Oxide Nanoparticles for T_1-Weighted Magnetic Resonance Imaging and Chemotherapy, ACS Nano 11 (2017) 10992-11004.
[45] H. Zhang, G.Q. Zhu, One-step hydrothermal synthesis of magnetic Fe_3O_4 nanoparticles immobilized on polyamide fabric, Appl. Surf. Sci. 258 (2012) 4952-4959.
[46] S.H. Sun, H. Zeng, D.B. Robinson, S. Raoux, P.M. Rice, S.X. Wang, G.X. Li, Monodisperse MFe_2O_4 (M = Fe, Co, Mn) Nanoparticles, J. Am. Chem. Soc. 126 (2004) 273-279.
[47] O. Alduhaish, M. Ubaidullah, A.M. Al-Enizi, N. Alhokbany, S.M. Alshehri, J. Ahmed, Facile Synthesis of Mesoporous α-Fe_2O_3@g-C_3N_4-NCs for Efficient Bifunctional Electro-catalytic Activity (OER/ORR), Sci. Rep. 9 (2019) 14139.
[48] W. Wu, S.L. Yang, J. Pan, L.L. Sun, J.H. Zhou, Z.G. Dai, X.H. Xiao, H.B. Zhang, C.Z. Jiang, Metal ion-mediated synthesis and shape-dependent magnetic properties of single-crystalline α-Fe_2O_3 nanoparticles, CrystEngComm 16 (2014) 5566-5572.
[49] J. Lin, J. Yu, O.U. Akakuru, X.T. Wang, B. Yuan, T.X. Chen, L. Guo, A.G. Wu, Low temperature-

boosted high efficiency photo-induced charge transfer for remarkable SERS activity of ZnO nanosheets, Chem. Sci. 11 (2020) 9414-9420.
[50] C. J. Orendorff, A. Gole, T. K. Sau, C. J. Murphy, Surface-Enhanced Raman Spectroscopy of Self-Assembled Monolayers: Sandwich Architecture and Nanoparticle Shape Dependence. Anal. Chem. 77 (2005), 3261-3266.
[51] J.R. Lombardi, R.L. Birke, T.H. Lu, J. Xu, Charge-transfer theory of surface enhanced Raman spectroscopy: Herzberg-Teller contributions, J. Chem. Phys. 84 (1986) 4174-4180.
[42] C. Boxall, G. Kelsall, Z. Zhang, Photoelectrophoresis of colloidal iron oxides. Part 2.-Magnetite (Fe_3O_4), J. Chem. Soc., Faraday T. 92 (1996) 791-802.
[43] M.E. Sadat, M.K. Baghbador, A.W. Dunn, H.P. Wagner, R.C. Ewing, J.M. Zhang, H. Xu, G.M. Pauletti, D.B. Mast, D.L. Shi, Photoluminescence and photothermal effect of Fe_3O_4 nanoparticles for medical imaging and therapy, Appl. Phys. Lett. 105 (2014) 091903.
[54] I. Leonov, A.N. Yaresko, V.N. Antonov, V.I. Anisimov, Electronic structure of charge-ordered Fe_3O_4 from calculated optical, megneto-optical Kerr effect, and O K-edge x-ray absorption spectra, Phys. Rev. B 74 (2006) 165117.
[55] E. Klevak, J.J. Kas, J.J. Rehr, Charge transfer satellites in x-ray spectra of transition metal oxides, Phys. Rev. B 89 (2014) 085123.
[56] X. Wu, L. Luo, S. Yang, X. Ma, Y. Li, C. Dong, Y. Tian, L. Zhang, Z. Shen, A. Wu, Improved SERS Nanoparticles for Direct Detection of Circulating Tumor Cells in the Blood, ACS Appl. Mater. Inter. 7 (2015) 9965-9971.
[57] T. Rajh, L.X. Chen, K. Lukas, T. Liu, M.C. Thurnauer, D.M. Tiede, Surface Restructuring of Nanoparticles: An Efficient R eliminatee for LigandMetal Oxide Crosstalk, J. Phys. Chem.B 106 (2002) 10543-10552.
[58] S. Wang, W. Ren, W.J. Hua, Z. Jiang, S. Madiha, L. Zhang, A. Li, A. Wu, Black TiO_2 based nanoprobes for T_1-weighted MRI guided photothermal therapy in CD133 high expressed pancreatic cancer stem-like cells. Biomater. Sci 6 (2018) 2209-2218.
[59] A.M. Brinkman, G.J. Chen, Y.D. Wang, C.J. Hedman, N.M. Sherer, T.C. Havighurst, S.Q. Gong, W. Xu, Aminoflavone-loaded EGFR-targeted unimolecular micelle nanoparticles exhibit anti-cancer effects in triple negative breast cancer, Biomaterials 101 (2016) 20-31.
[60] L. Guerrini, R.A. Alvarez-Puebla, Multiplex SERS Chemosensing of Metal Ions via DNA-Mediated Recognition. Anal. Chem. 91 (2019) 11778-11784.
[61] V. Ana, A. Miguel, M.R. Laura, R.A. Carlos, R. José, L.L. Rafael, C. Clotilde, L.J. Weon, EGFR-Based Immunoisolation as a Recovery Target for Low-EpCAM CTC Subpopulation, PLOS ONE 11 (2016) 0163705.
[62] K. Subik, J.F. Lee, L. Baxter, T. Strzepek, D. Costello, P. Crowley, L.P. Xing, M. Hung, T. Bonfiglio, D.G. Hicks, P. Tang, Expression Patterns ER, PR, HER2, CK5/6, EGFR, Ki-67 AR by Immunohis, Breast Canc. Basic Clin. Res. 4 (2010) 35-41.

Supporting Information

Multiple Valence States of Fe Boosting SERS Activity of Fe_3O_4 Nanoparticles and Enabling Effective SERS-MRI Bimodal Cancer Imaging

Jie Lin [a,*,1], Xuehua Ma [a,c,1], Anran Li [b,1], Ozioma Udochukwu Akakuru[a], Chunshu Pan[a], Meng He[a], Chenyang Yao[a], Wenzhi Ren[a], Yanying Li[a], Dinghu Zhang[a], Yi Cao[a], Tianxiang Chen [a,*] and Aiguo Wu [a,*]

[a]Cixi Institute of Biomedical Engineering, International Cooperation Base of Biomedical Materials Technology and Application, Chinese Academy of Science (CAS) Key Laboratory of Magnetic Materials and Devices, Zhejiang Engineering Research Center for Biomedical Materials, Ningbo Institute of Materials Technology and Engineering, CAS, 1219 Zhongguan West Road, Ningbo 315201, P. R. China. Advanced Energy Science and Technology Guangdong Laboratory, Huizhou 516000, P.R. China

[b]School of Engineering Medicine, Beihang University, Beijing, 100191, China; and Key Laboratory of Big Data-Based Precision Medicine (Beihang University), Ministry of Industry and Information Technology.

[c]University of Chinese Academy of Sciences, Beijing 100049, P. R. China.

*Corresponding authors.

E-mail addresses: linjie@nimte.ac.cn (J. Lin), chentx@nimte.ac.cn (T. Chen) aiguo@nimte.ac.cn (A. Wu)

[1]These authors contributed equally to this work.

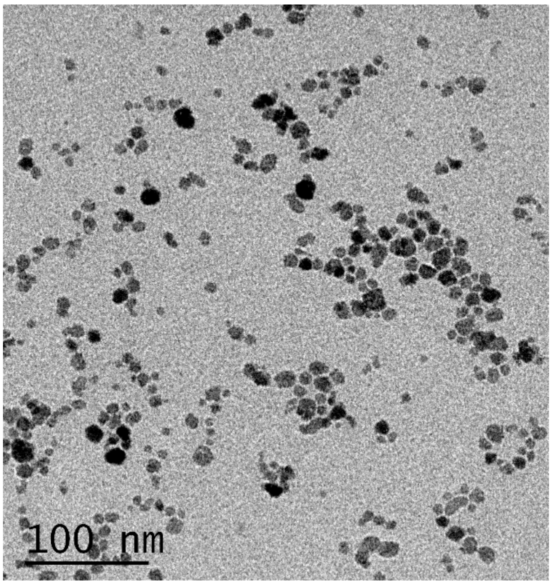

Figure S1. TEM image of ultrasmall Fe_3O_4 NPs.

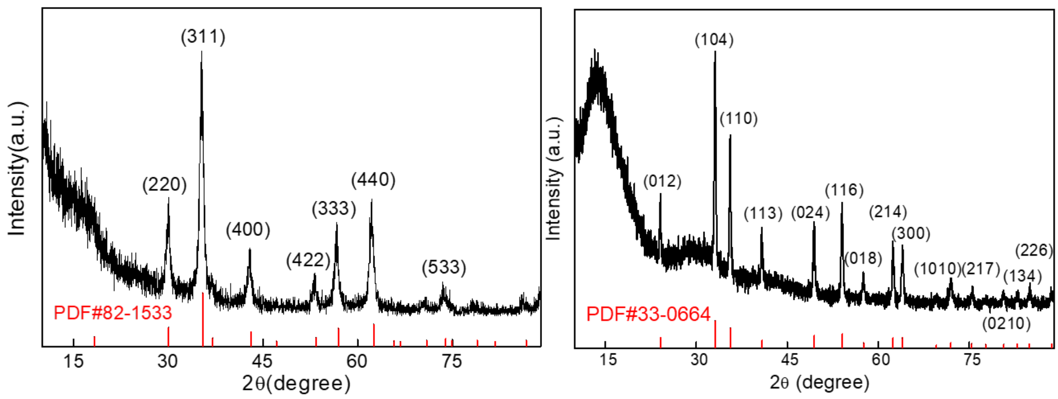

Figure S2. XRD characterizations of ultrasmall Fe_3O_4 and α-Fe_2O_3 NPs.

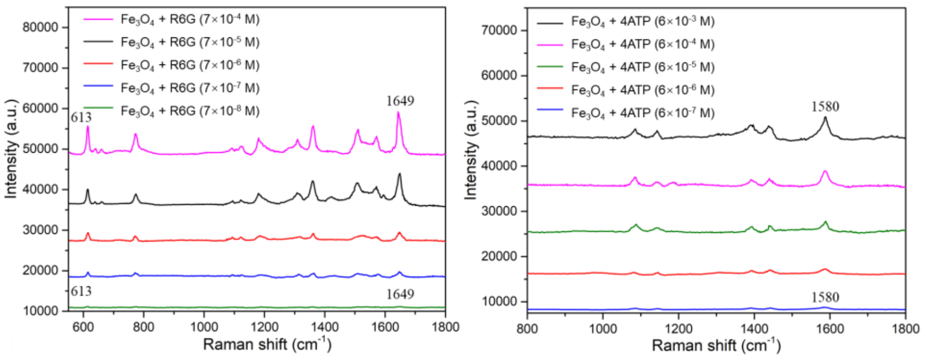

Figure S3. SERS spectra of different concentrations of R6G and 4ATP probe molecules adsorbed on Fe_3O_4 NPs. Laser wavelength: 532 nm; laser power: 0.5 mW; lens: 50 × objective; and acquisition time: 1 s.

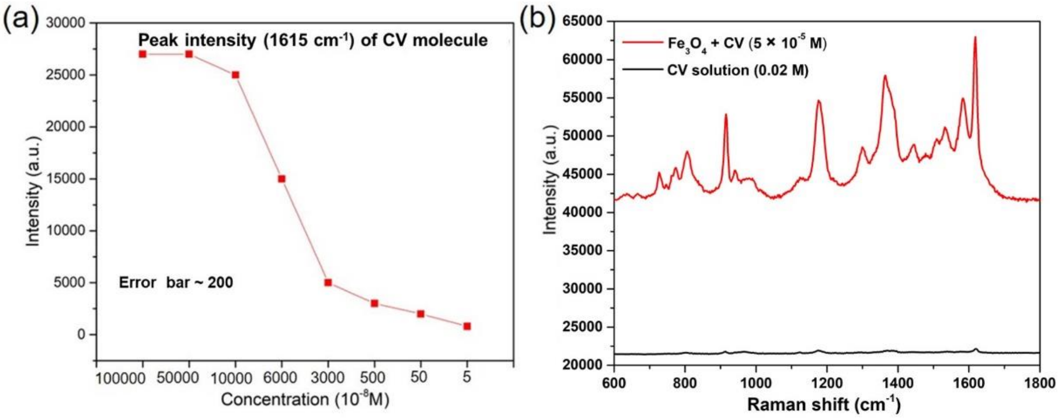

Figure S4. (a) SERS peak intensity (1615 cm^{-1}) of CV molecule adsorbed on Fe_3O_4 NPs at different concentrations. (b) The vibration peak intensity comparison between SERS spectra (Integration time: 1 s) and non-SERS solution spectra (Integration time: 2 s) of CV molecule.

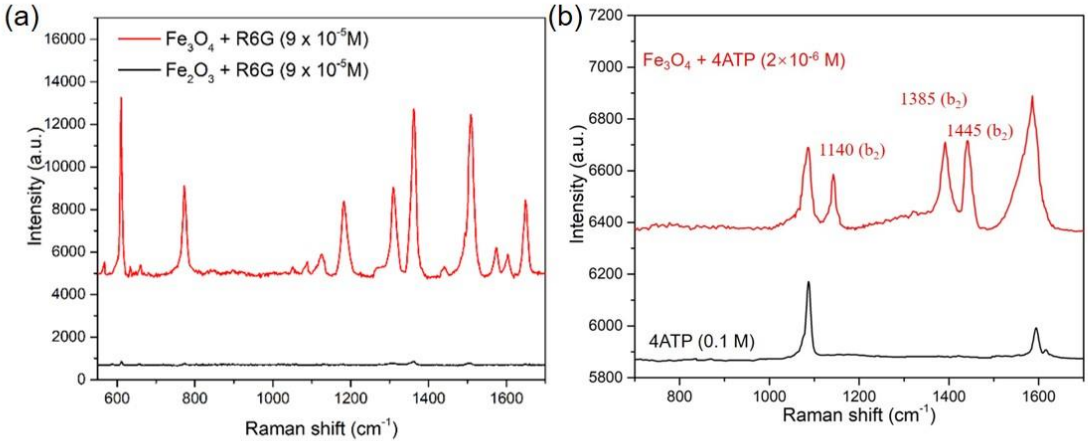

Figure S5. (a). SERS spectra comparison of R6G molecules adsorbed on Fe_3O_4 and Fe_2O_3 NPs, respectively. (b) SERS spectra of 4ATP molecules adsorbed on Fe_3O_4 NPs, and Raman spectra of pure 4ATP molecules. Laser wavelength: 532 nm; laser power: 0.5 mW; lens: 50 × objective; and acquisition time: 2 s.

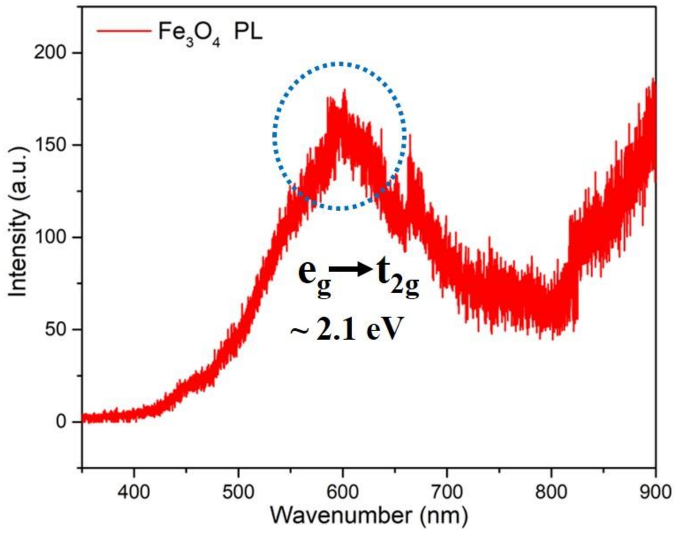

Figure S6. Photoluminescence (PL) spectroscopy of Fe_3O_4 NPs, ~ 590 nm peak is produced by radiative recombination of electrons transferred from e_g to t_{2g} crystal field band of octahedral site (Fe^{3+} and Fe^{2+}).

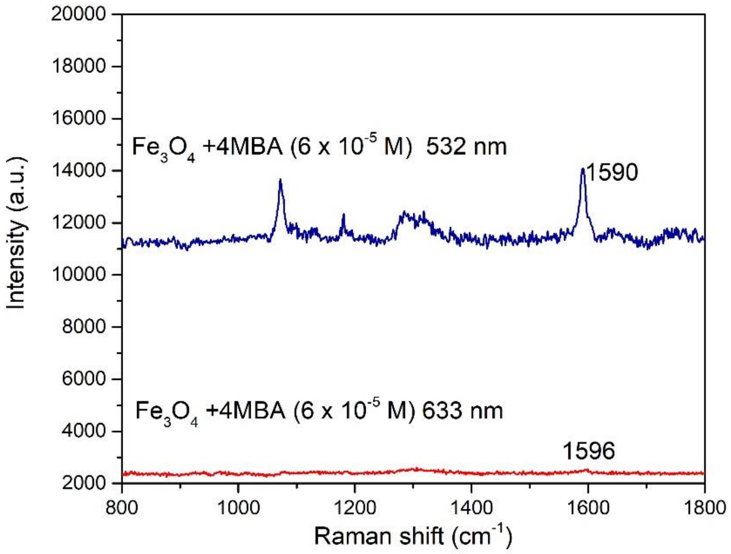

Figure S7. SERS spectra of 4MBA molecule (6×10^{-5} M) absorbed on Fe_3O_4 SERS substrate under different laser illuminations. Laser wavelength: 532 nm, 633nm; laser power: 0.5 mW; lens: 50 × objective; and acquisition time: 1 s.

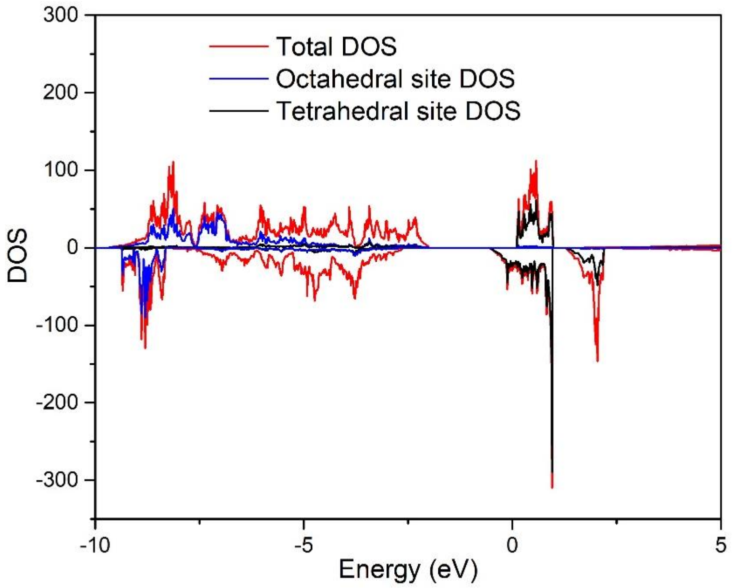

Figure S8. Total DOS, Fe ions exist on the octahedral, and tetrahedral sites DOS of ultrasmall Fe_3O_4 NPs.

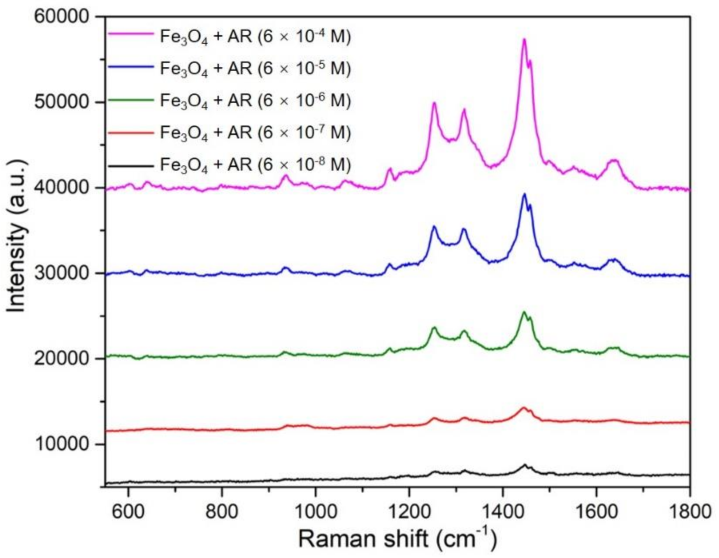

Figure S9. SERS spectra of AR molecule adsorbed on Fe_3O_4 NPs at different concentrations. Laser wavelength: 532 nm; laser power: 0.5 mW; lens: 50 × objective; and acquisition time: 1 s.

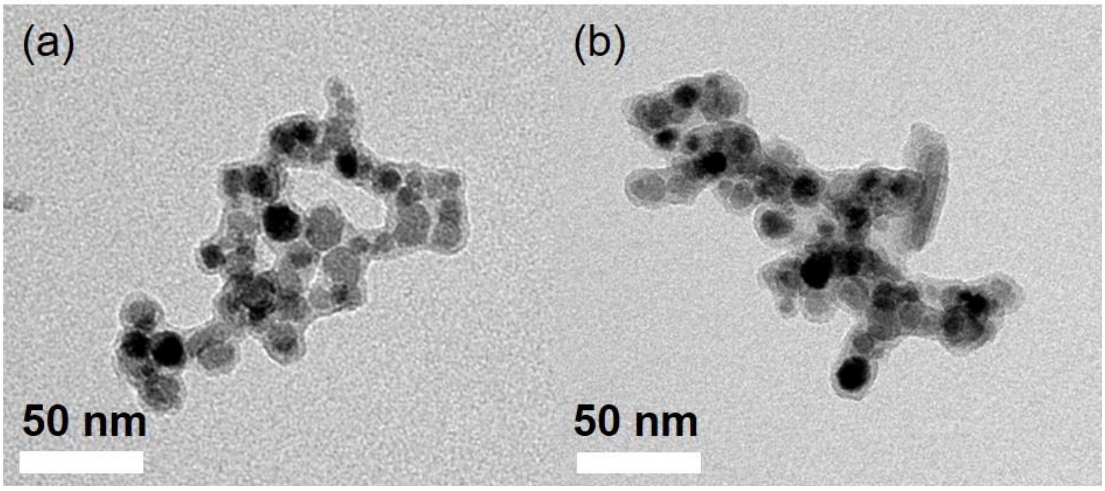

Figure S10. TEM images of Fe_3O_4-AR-PDA SERS bioprobes.

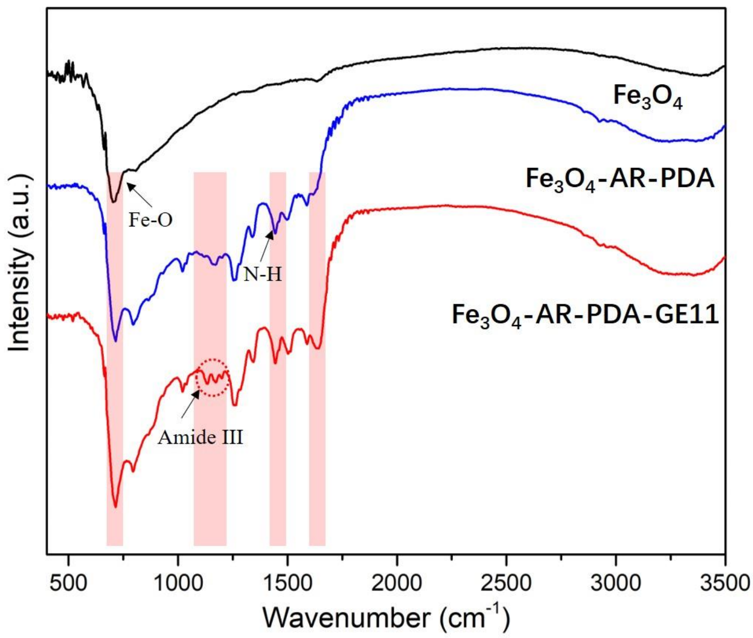

Figure S11. Fourier transform infrared spectra of Fe_3O_4, Fe_3O_4-AR-PDA, and Fe_3O_4-AR-PDA -GE11 SERS bioprobe.

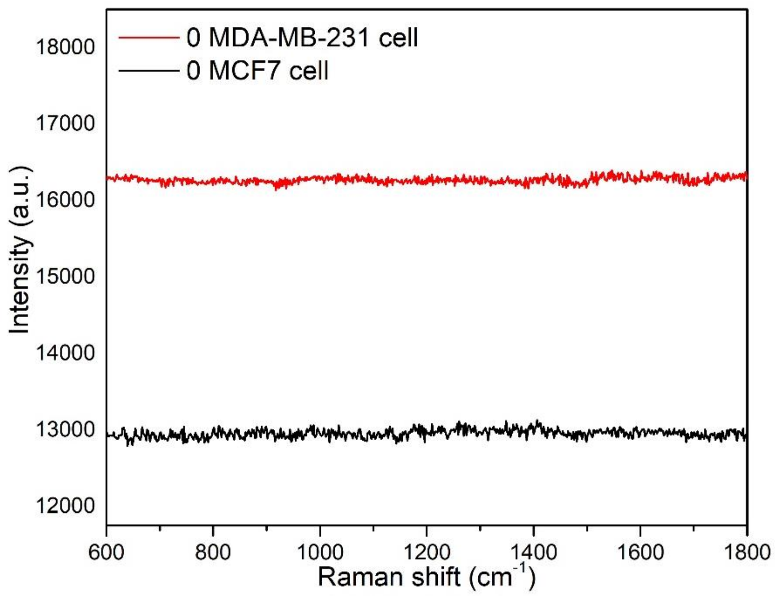

Figure S12. SERS signals of Fe_3O_4-AR-PDA-GE11 bioprobes without MCF7 and MDA-MB-231 cancer cell in rabbit blood. Laser wavelength: 532 nm; laser power: 0.2 mW; lens: 50 × objective; and acquisition time: 1 s.

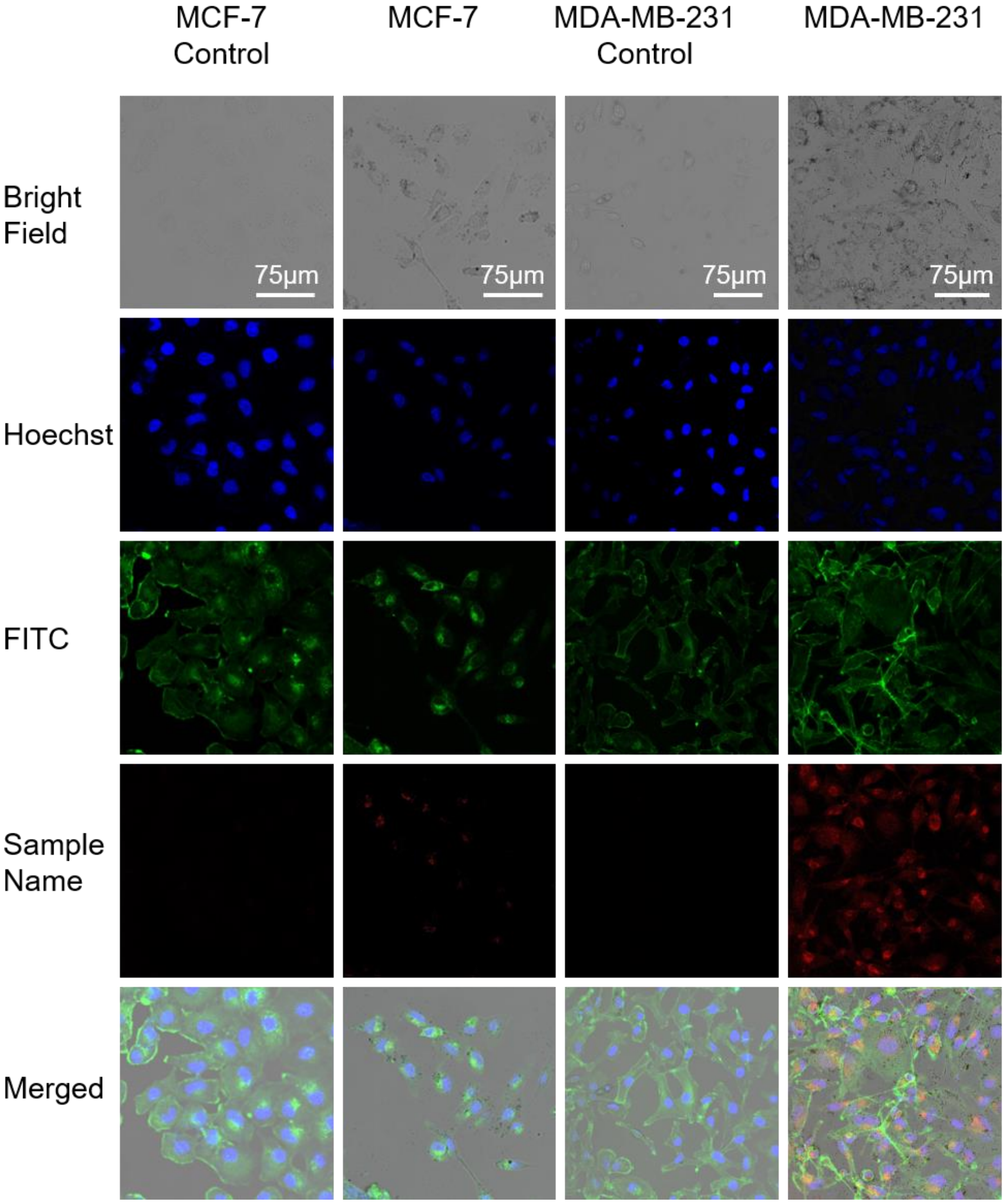

Figure S13. Laser scanning confocal microscope (LSCM) images of MCF-7 and MDA-MB-231 cancer cells co-incubated with Fe3O4-AR-PDA-GE11 SERS bioprobe for 4 h at 37 °C. The samples were simultaneously excited at 405, 488, and 552 nm. The cytoskeletons stained with FITC phalloidin are green (EM 600-680 nm) at an excitation of 552 nm, and the nuclei stained with Hoechst (EM 415-485 nm) are blue at an excitation of 405 nm. The AR are red (EM 506-576 nm) at an excitation of 488nm.

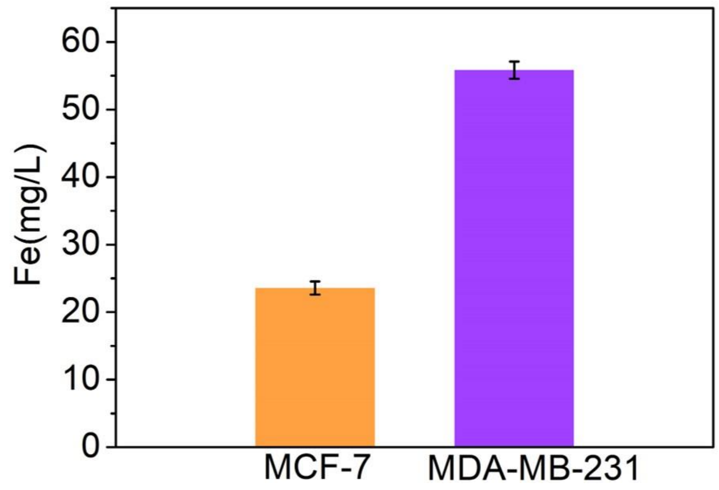

Figure S14. ICP-OES measurements of 1 mL Fe_3O_4-AR-PDA-GE11 SERS bioprobes (150 μg/mL) incubated with 400000 MCF-7, and MDA-MB-231 cancerous cells for 3 h.

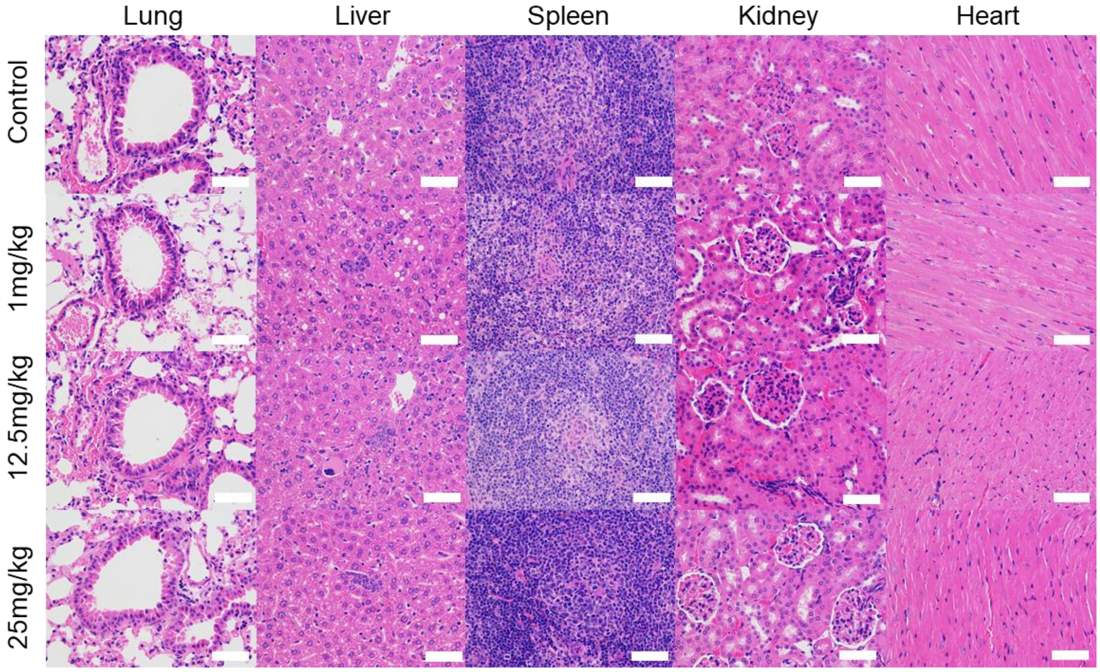

Figure S15. H&E staining of major organs collected from MDA-MB-231 TNB tumor-bearing mice after bioprobes intravenously injection for 14 days. (Scale bar = 50 μm)
